# G-quadruplex structures affect the immune response to bacterial infection in mouse macrophages

**DOI:** 10.1038/s41467-026-78610-w

**Published:** 2026-10-20

**Authors:** Philipp Schult, Melanie Kastl, Jennifer Szlapa, Konstantinos Symeonidis, Salie Maasewerd, Bernardo Franklin, Alea Radcke, Stefan A. Juranek, Zeinab Abdullah, Katrin Paeschke

**Affiliations:** 1 Department of Oncology, Hematology and Rheumatology, University Hospital Bonn, Bonn, Germany; 2 Institute of Clinical Chemistry and Clinical Pharmacology, University Hospital Bonn, Bonn, Germany; 3 Institute of Molecular Medicine and Experimental Immunology, University Hospital Bonn, Bonn, Germany; 4 Institute of Innate Immunity, University Hospital Bonn, Bonn, Germany

**Keywords:** Biochemistry, Cell biology

## Abstract

Macrophages orchestrate immunity via transcriptional reprogramming, largely controlled by the master regulator NF-κB. However, mechanisms of promoter selection remain enigmatic. Here, we reveal that DNA secondary structures called G-quadruplexes (G4s) modulate the macrophage transcriptome during inflammation. By investigating the G4 ligand pyridostatin (PDS) in lipopolysaccharide-stimulated macrophages, we establish that G4 stabilization significantly decreases the inflammatory response without affecting mitochondrial metabolism or pathogen clearance. Instead, NF-κB-dependent transcription of activation markers and proinflammatory cytokines is selectively repressed. Using chromatin accessibility profiling, NF-κB ChIP, and in silico predictions, we demonstrate that G4 stabilization sterically hinders NF-κB promoter occupancy. Furthermore, PDS stabilizes transient, transcription-induced G4s, prematurely shutting down transcription and reverting local chromatin to a dormant state. Thus, G4 formation represents a potential strategy to modulate dysregulated macrophage activation.

## Abstract (web summary)

G-quadruplexes (G4) are dynamic nucleic acid structures regulating various cellular functions. Here, the authors show that G4 formation sterically inhibits NF-κB-driven activation of pro-inflammatory genes, such as Il1b, acting as structural checkpoint to limit macrophage inflammatory responses.

## Introduction

Macrophages are central components of innate immunity, serving as the first line of defense against pathogens and neoplastic host cells. Through pattern recognition receptors (PRRs), they sense microbial and damage-associated molecular patterns (MAMPs/DAMPs) and orchestrate the inflammatory response via cytokine secretion, phagocytosis, and activation of the adaptive immune system^1^. Precise and dynamic regulation of these processes is critical, as dysregulated macrophage activation contributes to severe chronic inflammation, cancer, and infectious disease^2–4^. Among the key transcriptional regulators driving these programs, NF-κB (Nuclear factor kappa-light-chain-enhancer of activated B cells) functions as a central signaling hub coordinating cytokine expression and immune gene networks^5^. However, the multifaceted mechanisms of promoter selection by NF-κB under the volatile conditions of infection and immunity are still enigmatic^6^. The understanding of differential gene activation by NF-κB is clinically relevant, as it can tip the scale for immune switches like macrophage polarization^7^, leading to substantially altered disease outcomes. While global chromatin architecture clearly affects NF-κB occupancy^8^, local structures, such as G-quadruplexes (G4s) have not yet been studied in this context.

G4s are unique nucleic acid structures that form in guanine-rich regions of DNA and RNA^9^ harboring a consensus motif containing at least four closely spaced G-tracts^10^. More than 700,000 sequences in human and mouse genomes are proposed to fold into G4s, and many of these sites are located in functional genomic regions, where they play a crucial role in diverse gene regulatory networks. The general impact of G4-ligands, such as pyridostatin (PDS), has been extensively documented for central oncogenes like *c-MYC*^11–15^. However, it is now understood that the observed transcriptional perturbations by G4 ligands are highly complex, arising from a combination of direct G4 stabilization and broad, indirect cellular effects. In order to dissect these intricate regulatory networks, recent methodological advances have rapidly evolved, ranging from single-molecule biophysical platforms to novel locus-specific G4-targeting strategies^16–18^. Beyond single loci, global alterations in gene expression (positive and negative) have been observed following G4 stabilization, mediated by effects on polymerase progression^19–24^ or TF binding^20,25,26^. Besides their role in gene expression, folded G4 DNA structures can also impact telomere maintenance, DNA repair and DNA replication^27–33^.

While G4s have been shown to influence innate immune pathways in a context-dependent manner^34–38^, their contribution to specifically macrophage-dependent immune signaling is only beginning to emerge^39^. Indirect effects of G4s on macrophage behavior were shown, as G4 stabilization in tumor cells has been linked to type I interferon signaling and immunologic bias of the tumor microenvironment^34,40^.

Here, we report a novel role of G4 structures in modulating the transcriptomic landscape during macrophage activation. Using the G4-stabilizing ligand pyridostatin (PDS) in combination with bacterial lipopolysaccharide (LPS), we demonstrate that G4 formation exerts a marked suppressive effect on the macrophage inflammatory response. While core cellular functions such as phagocytosis, bacterial killing and mitochondrial activity remain intact, G4 stabilization selectively attenuates NF-κB-driven transcription and cytokine secretion. Mechanistically, by combining global NF-κB ChIP-seq with transcriptomic data, we demonstrate that G4 stabilization reduces NF-κB binding at a subset of inflammatory gene promoters, including *Il1b*. Subsequent in vitro binding assays support a model where G4 structures physically hinder NF-κB dimer binding to DNA. Thus, we suggest G4 folding as an important layer of gene regulation in macrophages and identify G4 stabilization as a potential means to control excessive inflammatory responses.

## Results

### G4 stabilization impairs activation of iBMDMs

A central aspect of macrophage function is the dynamic plasticity of their gene expression in response to external stimuli. This enables them to adapt to diverse environments, resulting either in the production of immunosuppressive cytokines (e.g., IL10) that maintain tissue homeostasis, or in the activation of strongly immunostimulatory programs (e.g., IL1B, IL18) that combat pathogens and cancer^41^. Given their enrichment in promoter regions and their established transcription factor binding properties^25,42^, G4s represent ideal tools for mediating rapid and reversible global genetic reprogramming. Furthermore, G4 formation is highly dynamic, demonstrating significant alterations in response to external stimuli or cell cycle phase. However, their formation and function in regulating macrophage activity remain poorly understood.

**Fig. 1:**
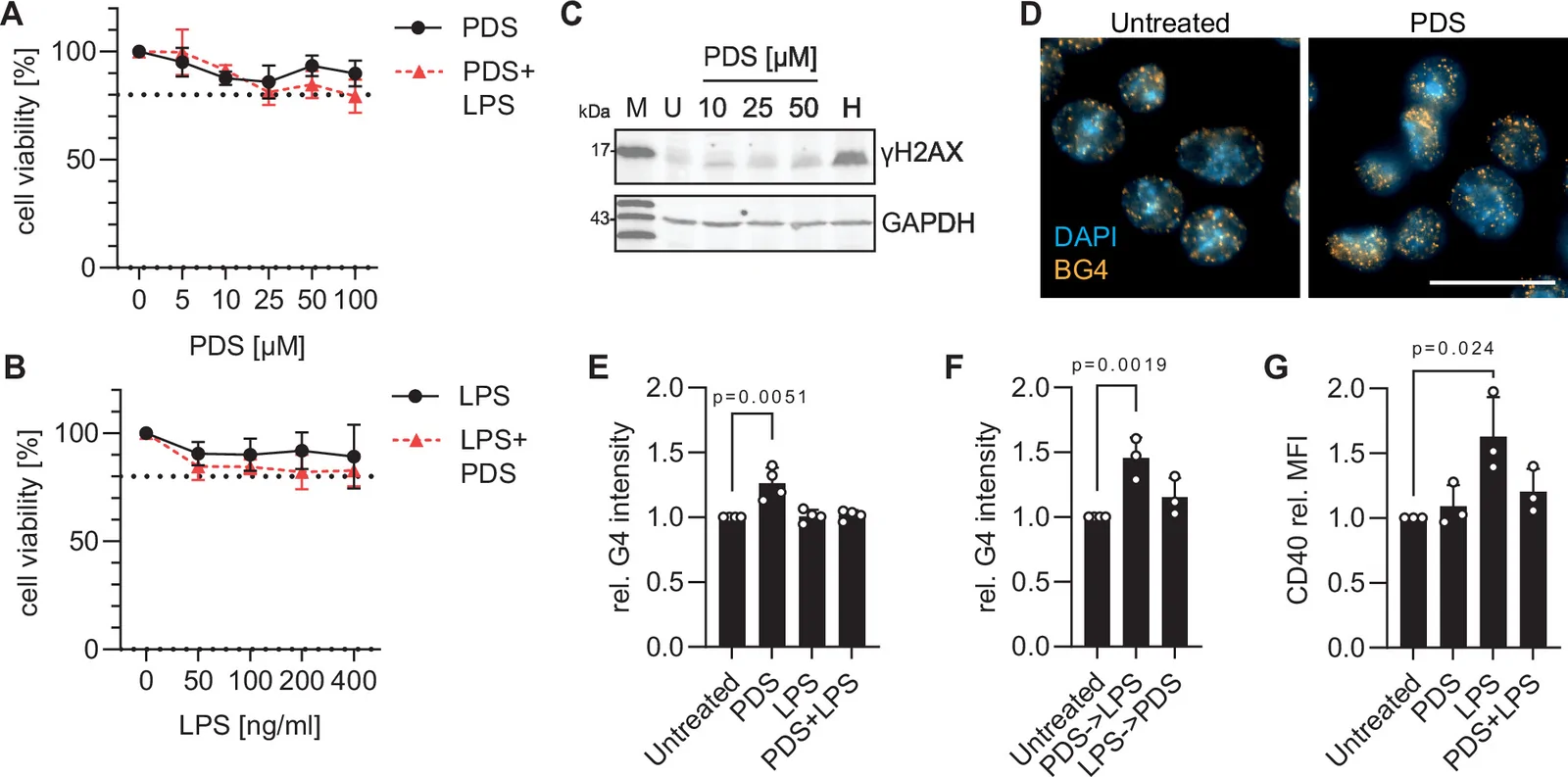
Impact of PDS and LPS on macrophages. **A**, **B** Viability assays using AlamarBlue after titration of PDS (**A**), LPS (**B**) in the presence or absence of the complementary compound. **C** Examination of genome instability upon PDS treatment by γH2A.X western blot. U: untreated, H: 1 mM H_2_O_2_ (positive control). **D** Representative immunofluorescence image of untreated and PDS-treated iBMDMs, stained with BG4. Scale bar represents 20 µm. Experiments were performed three times with similar results. **E** Quantification of nuclear G4 intensity from IF images after single and double treatment with LPS and PDS. **F** As in E but with iterative treatments, either 4 h PDS followed by 4 h LPS or vice versa. **G** FACS analysis of surface marker CD40 is indicative of macrophage activation under different conditions. Data are represented as mean ± S.D. All experiments were performed in three independent biological replicates. Statistical significance (two-tailed Student’s *t*-test) is represented by the *p*-value. Source data are provided as a Source Data file.

Therefore, we aimed to investigate whether G4 induction can modulate the early phases of macrophage activation (up to 4 h). To this end, we employed immortalized mouse bone marrow-derived macrophages (iBMDMs)^43^ and treatment with two well-established compounds: the G4 ligand PDS^44^, to promote G4 formation, and LPS, as a mimic of bacterial infection. To determine non-toxic concentrations of PDS and LPS, we first assessed cell viability using AlamarBlue after single or combined treatments at various concentrations over 4 h. We found that cell viability remained unaffected up to 100 µM PDS when applied alone. However, in combination with 200 ng/ml LPS, concentrations above 25 µM PDS resulted in cytotoxic effects (Fig. 1A). In contrast, LPS treatment maintained high cell viability with minimal dose dependency across the tested range of 50–400 ng/ml, in presence or absence of 25 µM PDS (Fig. 1B). Since high doses or prolonged exposure to PDS have been reported to induce DNA damage^34^, which could markedly distort the transcriptomic landscape, we examined phosphorylated H2A.X (γH2A.X) levels as a marker of DNA double-strand breaks by western blotting. PDS treatment up to 50 µM for 4 h did not induce detectable DNA damage, whereas H₂O₂ treatment, used as a positive control, led to a clear accumulation of γH2A.X (Fig. 1C). Based on these results, all subsequent experiments were conducted with 25 µM PDS and 200 ng/ml LPS.

Next, we tested whether naïve iBMDMs contain G4 structures and whether PDS, LPS, or their combination affects G4 formation using the G4 antibody BG4^45^ in immunofluorescence microscopy (Fig. 1D) and flow cytometry (BG-Flow, Supplementary Fig. 1A, B). As expected, PDS treatment led to a consistent increase in G4 signal, whereas LPS alone had no detectable effect. Intriguingly, the combination of both compounds abolished the G4 accumulation observed with PDS alone (Fig. 1E and Supplementary Fig. 1B).

To determine whether this effect resulted from impaired formation or enhanced removal of G4s, we performed pretreatment experiments with either compound for 4 h before adding the other. We found that once G4s were formed and stabilized by PDS, subsequent LPS treatment did not reverse this effect (Fig. 1F). In contrast, LPS pretreatment effectively prevented G4 stabilization by PDS, suggesting a loss of G4 formation due to LPS treatment. To assess whether these opposing molecular effects translate into functional consequences, we analyzed the expression of common LPS-dependent macrophage activation markers (CD40, CD80) by flow cytometry. Already after 4 h, LPS treatment increased CD40 and CD80 surface expression; however, this effect was absent upon PDS treatment alone or co-treatment with PDS and LPS (Fig. 1G and Supplementary Fig. 1C–E). To exclude that this phenotype was caused by indirect effects of PDS on macrophage functionality, we interrogated several integral functions: Primarily, mitochondrial metabolism is known to critically influence macrophage function, as changes in biogenesis, membrane potential, and ROS generation accompany transitions between pro- and anti-inflammatory states^44–46^. Thus, we examined whether PDS affects mitochondrial function using mitochondrial mass (MitoTracker Green), membrane potential (MitoTracker Red), and superoxide production (MTSOX), a key contributor to inflammatory signaling and bacterial killing. Across all treatments, none of the tested parameters were negatively affected, suggesting that core mitochondrial functions required for macrophage activation and inflammatory responses remain intact (Supplementary Fig. 1F–H). Likewise, other key macrophage functions upstream of transcriptional reprogramming, such as uptake and phagocytic killing, were unaffected, as shown by a FACS-based uptake assay using mCherry-labeled *Salmonella* (Supplementary Fig. 1I) and colony-forming unit (CFU) assay after co-culture with *Listeria* (Supplementary Fig. 1J).

Overall, our data confirmed that macrophages harbor endogenous G4 structures that can be chemically stabilized with non-cytotoxic concentrations of PDS. Moreover, we demonstrate that PDS and LPS functionally counteract each other, resulting in impaired LPS-dependent macrophage activation upon PDS-mediated chemical G4 stabilization. Importantly, mitochondrial function and phagocytosis remain unchanged, suggesting that PDS-dependent effects occur downstream of these central metabolic pathways at the transcriptional level.

### PDS impairs NF-κB-dependent transcription activation

Given the pronounced suppression of macrophage activation markers upon G4 stabilization, we next sought to outline the transcriptional mechanisms underlying this phenotype. Because G4s are known to modulate transcription factor (TF) recruitment and polymerase processivity, we postulated that G4 stabilization may interfere at the stage of transcriptional reprogramming during inflammatory activation.

**Fig. 2:**
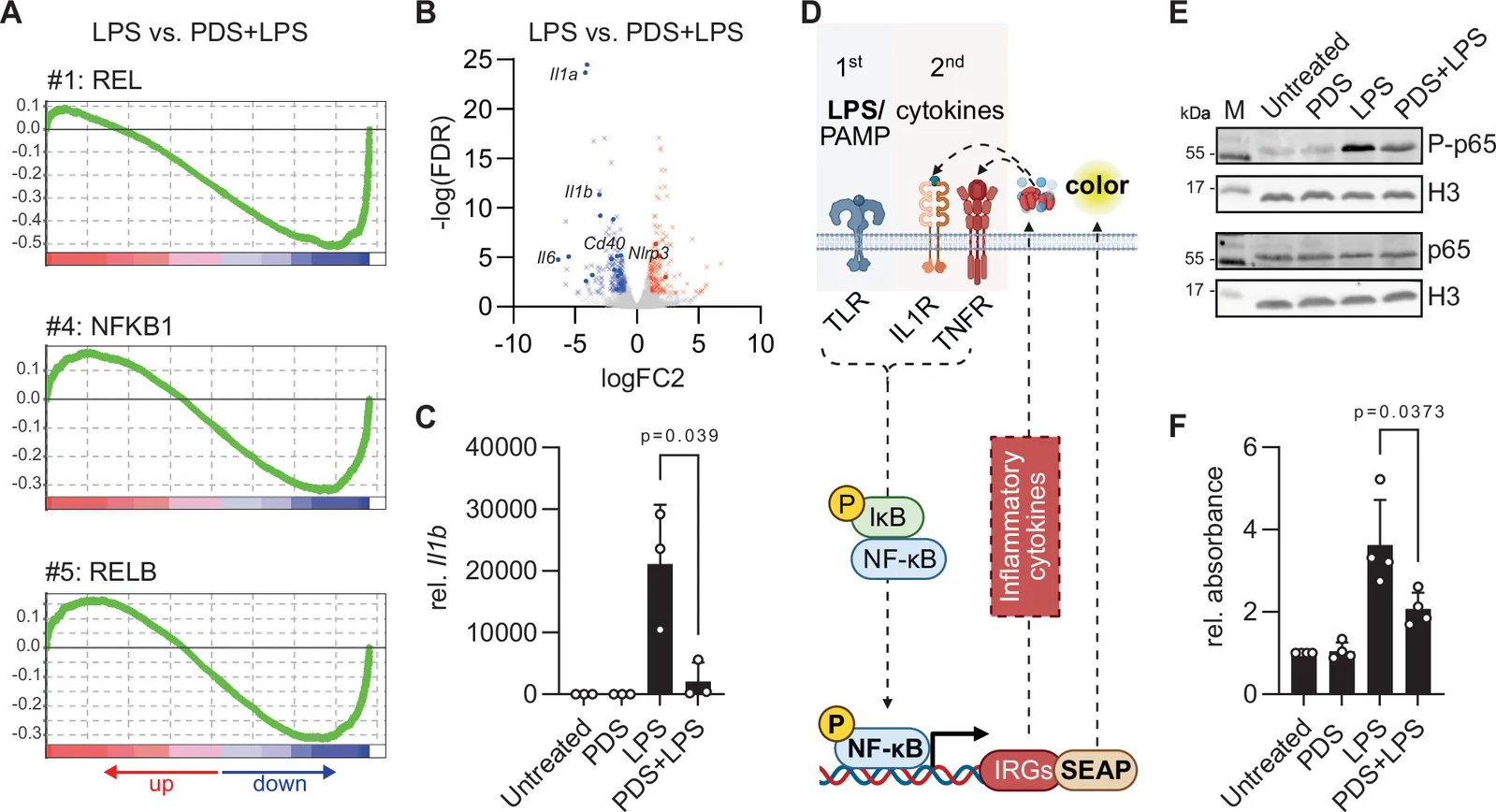
Link between G4 stabilization and NF-κB-driven transcriptional activity. **A** Ridge plots from gene set enrichment analysis, comparing gene expression in response to LPS versus PDS + LPS treatment. Shown are top 1, 4 and 5 negatively enriched TF pathways, all associated with NF-κB signaling. **B** Volcano plot of RNASeq analysis used in A. Larger dots are significantly regulated, high-confidence NF-κB targets. Selected important proteins are labeled separately. **C** Single target validation of RNASeq data via qPCR for all tested conditions, using Il1b as example (*n* = 3). **D** Overview of the pathways leading to NF-κB activation and inflammatory response genes (IRGs), as well as the principle of SEAP-based quantification. Created in BioRender. Paeschke, K. (2026) https://BioRender.com/lsckkv2. **E** Western blot analysis of NF-κB p65 phosphorylation (S536) and protein expression. **F** SEAP-based quantification of NF-κB activity with the QuantiBlue assay kit (*n* = 4). Data are represented as mean ± S.D. All experiments were performed in at least three independent biological replicates. Statistical significance (two-tailed Student’s *t*-test) is represented by the *p*-value. Source data are provided as a Source Data file.

To test this hypothesis, we performed 3′ RNA-Seq on iBMDMs stimulated with LPS in the presence or absence of PDS. Gene set enrichment analysis (GSEA) of differentially expressed genes revealed that, besides MLLT3 and CRELD1, three of the five most significantly downregulated pathways corresponded to the NF-κB family (REL, NF-κB1, and RELB), suggesting that G4 stabilization specifically targets NF-κB signaling (Fig. 2A). These data were corroborated by matching a curated list of high-confidence NF-κB targets (https://esbl.nhlbi.nih.gov/Signaling-Pathways/NF-κB-Targets/) against the significantly regulated genes from the RNAseq data set. Indeed, in agreement with the GSEA, we observed a total of 27 genes that were significantly dysregulated; of these, 23 exhibited a PDS-dependent decrease in expression. Among them, several important pro-inflammatory cytokines (e.g., *Il1a*, *Il1b*, *Il6*) and the maturation marker CD40, in accordance with previous observations (Figs. 2B and 1G). To validate these findings, we examined *Il1b* expression as a representative NF-κB target using qPCR-based quantification. In accordance with the RNASeq data, LPS stimulation robustly induced *Il1b* mRNA levels, whereas co-treatment with PDS nearly abolished this induction, reverting expression to basal amounts (Fig. 2C).

To test the hypothesis that G4 stabilization by PDS does not merely induce general transcriptional silencing but selectively abrogates the pro-inflammatory activation program while leaving counter-regulatory feedback loops intact, we performed enrichment analyses using Metascape^46^. To this end, we selected a subset of genes, which were at least 2-fold upregulated by LPS (log_2_FC > 1) compared to untreated, but not negatively affected by PDS cotreatment (ΔLog_2_FC > −0.3, Supplementary Data 1). Several immune-relevant pathways were among the significantly enriched GO terms, most notably, the ‘negative regulation of cytokine production’ (GO:0001818, Supplementary Fig. 2A). Moreover, PaGenBase^47^ analysis identified signature genes of unstimulated macrophages (PGB:00277) as the only significant hit from these genes.

Next, we sought to determine at which step of LPS-dependent signaling the function of NF-κB was impaired. The primary activation of NF-κB is induced by the binding of LPS to TLR4, which results in NF-κB phosphorylation via IκB, dimerization, and translocation to the nucleus. The production of cytokines instigates a positive feedback loop, which in turn leads to an additional increase in NF-κB phosphorylation (Fig. 2D). Western blot analysis of phospho-p65 (Ser536) and phospho-RELB (Ser552) revealed that phosphorylation still occurred under PDS + LPS co-treatment, albeit at reduced levels, suggesting that primary upstream signaling is initiated but quantitatively dampened (Fig. 2E). A similar phenotype was observed for RELB, which was also detected in the PDS-dependent GSEA signature (Supplementary Fig. 2B, C). In contrast, neither LPS nor PDS had a significant impact on phosphorylation status (Ser337) or expression of NF-κB1 p50 (Supplementary Fig. 2B, C). Moreover, the transcriptional output closely resembled the effects on *Il1b* transcription, as confirmed by an NF-κB-responsive SEAP reporter assay in RAW cells, which showed a significant loss of reporter activity in response to PDS + LPS compared to LPS alone (Fig. 2F).

Together, these findings demonstrate that G4 stabilization likely does not affect the TLR4-mediated NF-κB activation axis but instead impairs the transcriptional activity of NF-κB-dependent gene expression. This points towards a model in which G4 formation interferes with promoter accessibility or TF binding rather than upstream signaling.

### G4 formation compromises the functional inflammatory response

After establishing that G4 stabilization disrupts NF-κB-driven transcription, we next examined whether these transcriptional defects translate into impaired inflammatory signaling at the protein level. Cytokine secretion represents a key effector function of activated macrophages and depends on the coordinated transcription of NF-κB-regulated genes^48^.

**Fig. 3:**
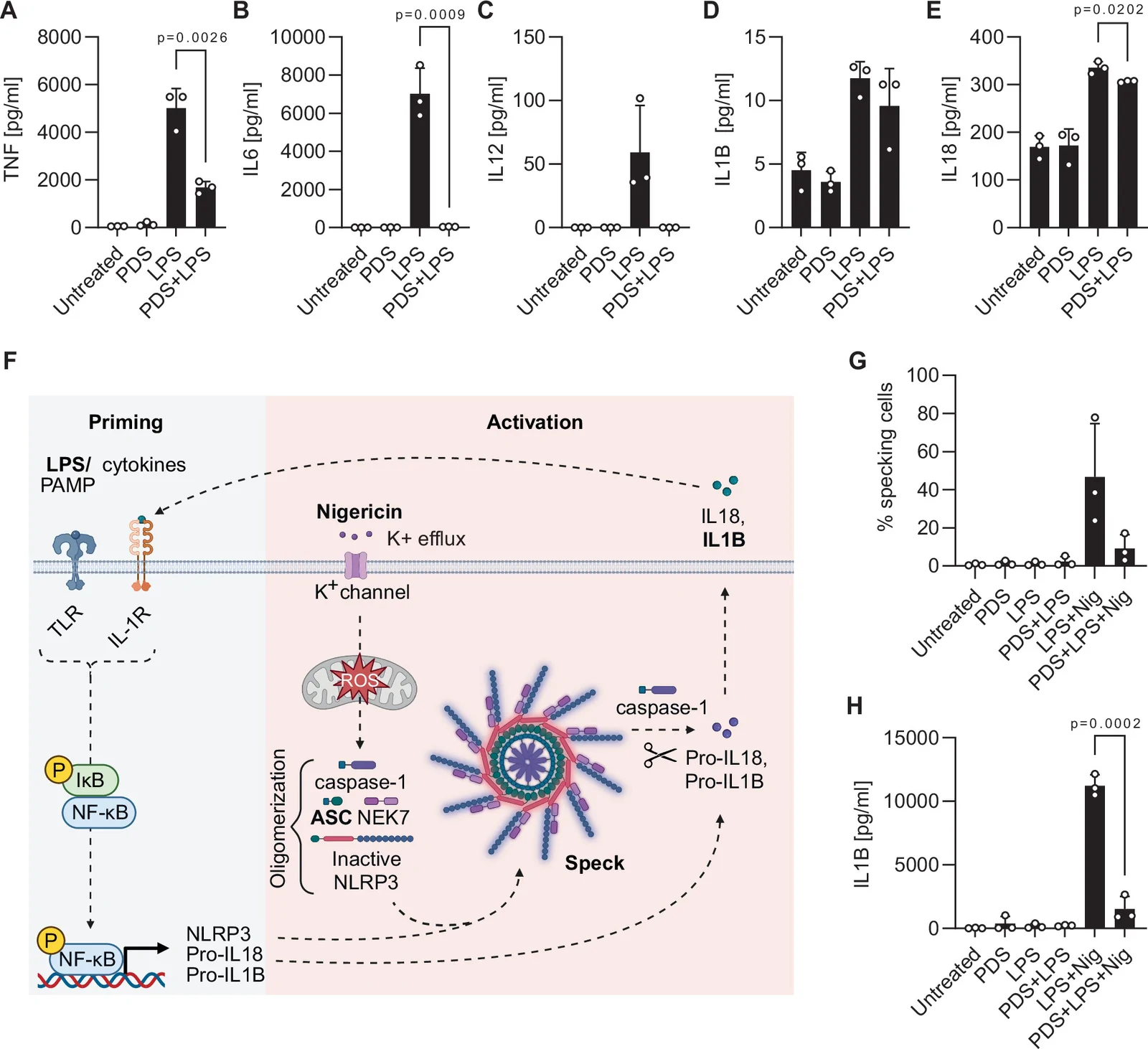
Downstream effects of the PDS-dependent transcriptional block of NF-κB. **A**–**E** Secretion of important inflammatory cytokines, determined by ProcartaPlex measurement. **F** Scheme of NF-κB-dependent inflammasome activation and positive feedback loop via cytokine secretion. Created in BioRender. Paeschke, K. (2026) https://BioRender.com/s1kv2yn. **G** Measurement of ASC aggregation as a marker for inflammasome activation. **H** ProcartaPlex measurement of IL1B secretion at 8 h post stimulation as an indicator of downstream effects, e.g., caspase 1 activity of the assembled NLRP3 inflammasome. Data are represented as mean ± S.D. All experiments were performed in three independent biological replicates. Statistical significance (two-tailed Student’s *t*-test) is represented by the *p*-value. Source data are provided as a Source Data file.

To quantify the impact of G4 stabilization on cytokine release, we measured the extracellular levels of major inflammatory mediators after treatment with PDS, LPS, or both using a ProcartaPlex assay. Consistent with the transcriptomic data, PDS alone did not induce detectable cytokine secretion, whereas LPS stimulation strongly induced TNF, IL6, IL12, IFNB, IL10, and CXCL10. Remarkably, co-treatment with PDS almost completely reverted this induction across most tested cytokines (Fig. 3A–C and Supplementary Fig. 3A–D). These findings confirm that chemical G4 stabilization blocks the functional output of NF-κB signaling and interrupts the autocrine feedback loop that sustains macrophage activation.

Of note, IL1B and IL18 displayed comparatively low induction upon LPS stimulation and only minor reductions upon co-treatment with PDS (Fig. 3A–D, E). This discrepancy reflects their specific mode of regulation: both cytokines are produced as inactive cytosolic precursors (pro-IL1B and pro-IL18) that require proteolytic processing by caspase-1, which is activated by inflammasome assembly. In macrophages, this process is functionally divided into two stages (Fig. 3F). During the **“**priming**”** phase, LPS engagement of TLR4 triggers NF-κB-dependent transcription of cytokines but also effectors such as *Nlrp3*, the central component of inflammasome assembly. A subsequent **“**activation**”** signal, such as ATP depletion, pore-forming toxins, or the bacterial antibiotic nigericin, induces potassium efflux and mitochondrial stress, promoting the oligomerization of NLRP3 and the adaptor ASC (apoptosis-associated speck-like protein containing a CARD).

To experimentally reproduce this two-step mechanism, we first primed macrophages with LPS and then activated the full inflammasome response using nigericin. To directly visualize inflammasome assembly, we employed immortalized mouse macrophages expressing an ASC–mCerulean fusion protein. Upon inflammasome activation, ASC forms microscopically visible aggregates known as ASC specks, which were used as a quantitative readout of inflammasome assembly in live cells.

Expectedly, combined LPS + nigericin treatment strongly induced ASC speck formation, confirming robust inflammasome activation, while the addition of PDS during priming markedly reduced the number of ASC aggregates (Fig. 3G). This observation corroborated the reduced transcription observed in the RNA-Seq data and translation (Supplementary Fig. 3F) of pro-inflammatory factors and indicates that the deficient priming is responsible for impaired inflammasome assembly. In contrast, the addition of PDS only during nigericin treatment did not affect the functionality of the inflammasome, as Caspase-1 and IL1B maturation and secretion remained unaffected (Supplementary Fig. 3F).

To confirm the functional consequences of this defect, we measured secretion of mature IL1B at 4 h and 8 h post-stimulation (Fig. 3H, Supplementary Fig. 3E). As expected, 8 h LPS + nigericin treatment induced a > 1000-fold increase in IL1B secretion relative to LPS alone (Fig. 3H). In contrast, co-treatment with PDS substantially reduced IL1B release at both time points, with a particularly strong effect after 8 h (Fig. 3H and Supplementary Fig. 3E).

In sum, these results demonstrate that G4 stabilization suppresses NF-κB-dependent expression of pro-inflammatory cytokines, which results in a loss of the subsequent inflammasome activation cascade, effectively dampening the inflammatory response. By interfering with the priming step and limiting *Nlrp3* expression, PDS-induced G4 formation also constrains inflammasome-mediated caspase-1 activation and cytokine maturation. Thus, induction of G4s in promoter regions is a critical checkpoint that can restrict the initiation and escalation of macrophage-mediated inflammation.

### G4s overlapping NF-κB binding sites directly inhibit p65 DNA association

**Fig. 4:**
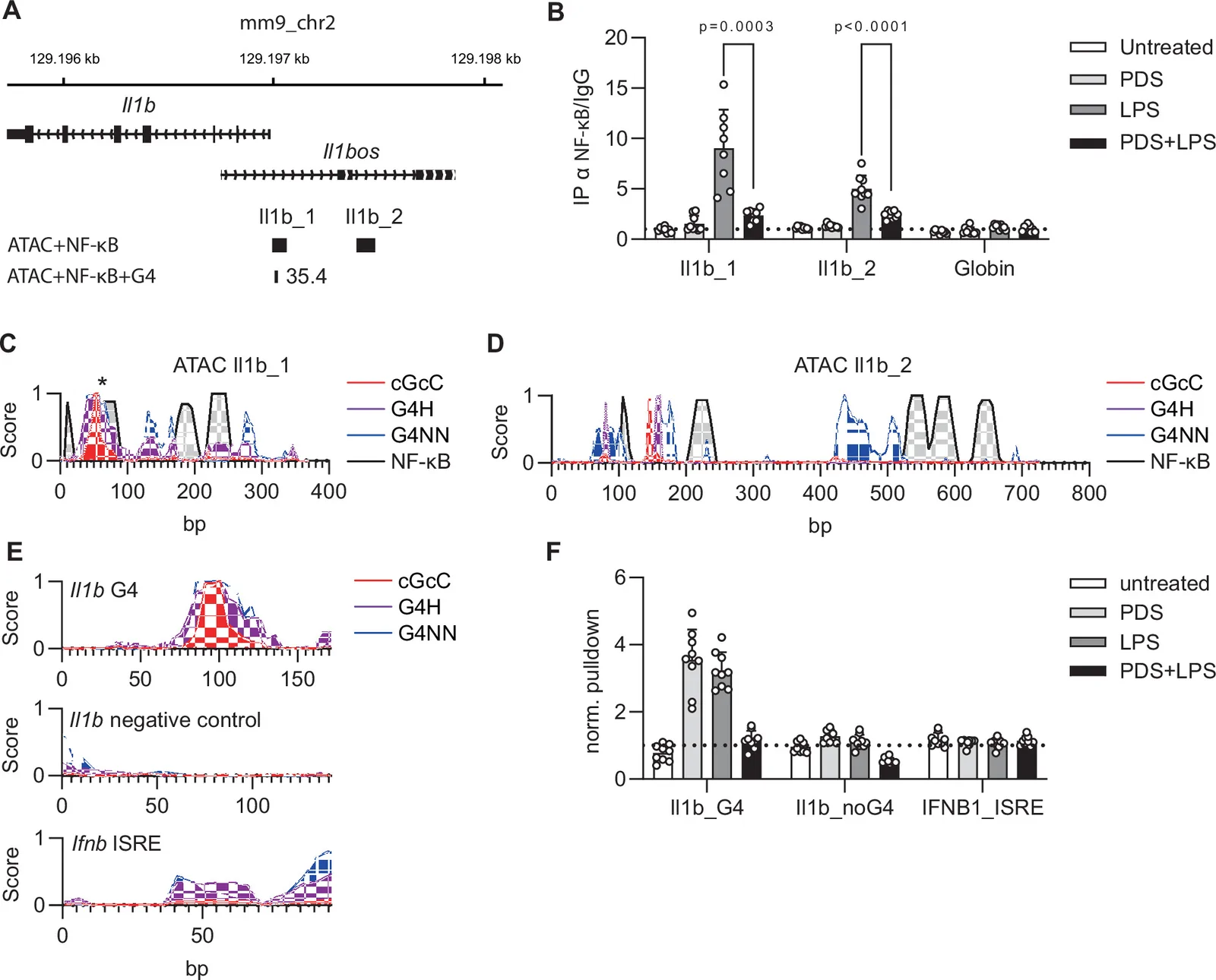
G4-dependence of NF-κB binding to DNA. **A** Genomic locus of Il1b. ATAC + NF-κB denotes regions of accessible chromatin, overlapping known NF-κB binding sites after LPS treatment^49^. ATAC + NF-κB + G4 indicates G4s detected via previous G4seq analyses^50^. **B** ChIP-qPCR assessment of NF-ΚB association to the selected genomic loci under different treatment conditions. **C**, **D** Predicted overlaps of G4-forming regions (G4RNA Screener) and NF-κB binding motifs (HOCOMOCO/TFBIND) in the Il1b_1 (**C**) and Il1b_2 (**D**) site. Data are normalized to an IgG control ChIP performed in parallel. The coding region of the globin gene was used as a background control. Data are represented as mean ± S.D. ChIP-qPCR experiments were performed in biological and technical triplicate. All replicate means are shown as symbols. **E** Prediction of G4-forming regions within the selected targets for G4DP qPCR. For direct comparison, all scores are normalized to the maximal value of the Il1b G4 sequence. **F** G4DP qPCR results for the selected targets, showing the normalized pulldown efficiencies relative to input and the globin CDS. Statistical significance (two-sided two-tailed Student’s *t*-test) is represented by the *p*-value. Source data are provided as a Source Data file.

After examination of the functional impact of chemical G4 stabilization on the inflammatory response, we explored the molecular mechanism underlying this phenomenon. We postulated that G4s might interfere directly with NF-κB binding sites due to the presence of a G triplet within the consensus motif (GGGRNYYYCC), which might be engaged in a G4, depending on the genomic context. To this end, we performed ATAC seq (Assay for Transposase-Accessible Chromatin using sequencing) to identify sites of transcriptional activity upon LPS treatment. Additionally, previously published data from Peterson et al.^49^ were utilized to identify LPS-dependent NF-κB binding regions in macrophages (ATAC + NF-κB, ~20%, Supplementary Fig. 4A). As anticipated, two of these sites (Il1b_1 and Il1b_2) were identified within approximately 1000 bp upstream of the *Il1b* transcription start site (Fig. 4A). Furthermore, these regions were intersected with G4seq data from Marsico et al.^50^ to identify candidate G4s adjacent to NF-κB target sequences (ATAC + NF-κB + G4, ~20%, Supplementary Fig. 4B). The ratio of G4 containing ATAC site with NF-κB target sequences was lower, compared to all ATAC sites ( ~ 50%, Supplementary Fig. 4C). Intriguingly, a strong G4 (“35.4” as defined by Marsico et al.^50^) was identified in the Il1b_1 region, directly adjacent to the *Il1b* transcription start site (TSS, Fig. 4A). To test the hypothesis that stabilized G4s actively deter NF-κB dimers from their binding sites, we performed ChIP-qPCR targeting the identified Il1b_1 and Il1b_2 regions. Upon treatment with LPS, a distinct association with these DNA segments was identified for NF-κB, which was substantially diminished upon the addition of PDS (Fig. 4B). Of note, the observed quantitative difference in NF-κB pull-down efficacy between the two sites mirrors the occupancy found by Peterson et al.^49^. PDS alone did not alter NF-κB association with the DNA. No detectable DNA occupancy was observed for primers targeting the coding sequence (CDS) of globin in any condition (Fig. 4B). To assess in more detail if these regions encompass potential sites where G4-formation and NF-κB binding might come in conflict, in silico G4 predictions were performed using G4RNA screener (combining the algorithms cGcG, G4Hunter and G4NN), as well as NF-κB binding site predictions via the HOCOMOCO database and TFBIND. A most intriguing discovery of this analysis was the clear overlap between the Marsico 35.4 G4 and a highly scored NF-κB motif (Fig. 4C). Within the Il1b_2 region, partial overlaps were also detected (Fig. 4D). These results are in good agreement with the observed loss of NF-κB occupancy upon PDS treatment. To explore the dynamic relationship between G4 formation and transcription factor occupancy at the *Il1b* locus, we performed a chemical G4 pull-down assay using a modified version of the G4-DNA precipitation (G4DP) protocol established by Feng et al.^51^, as classical BG4-ChIP approaches can be limited by steric competition between the antibody and small-molecule ligands. The specificity of the assay was confirmed by a robust, PDS-dependent enrichment of the G4-containing region within the *Il1b* promoter, whereas no increase was observed at non-G4 control regions within the *Il1b* CDS or the *Ifnb* promoter (Fig. 4E, F). Interestingly, LPS stimulation alone also significantly increased the G4 pull-down efficiency (Fig. 4F). This is consistent with the assumption that under basal conditions, the *Il1b* promoter remains transcriptionally dormant, thus rarely adopting a single-stranded conformation. Upon LPS activation, the massive transcriptional induction dynamically generates transient single-stranded DNA intermediates within the transcription bubble, substantially increasing the kinetic frequency of G4 formation. Finally, co-treatment with PDS and LPS reduced the pull-down efficiency back to basal levels (Fig. 4F). This finding aligns well with our immunofluorescence and FACS-based observations that global G4-induction is lost in the PDS + LPS conditions (Fig. 1E and Supplementary Fig. 1A).

In summary, we demonstrate that chemically stabilized G4s can actively impede NF-κB binding to target sequences, thereby providing a strong molecular basis for the observed transcription defects. Additionally, the G4DP data points toward a transcriptional feedback mechanism: due to the early blockade of NF-κB by PDS, the recruitment of RNA polymerases is inhibited, leading to the collapse of the transcription bubble and reversion to the dormant state.

### Steric hindrance by G4 formation inhibits the binding of NF-κB dimers

**Fig. 5:**
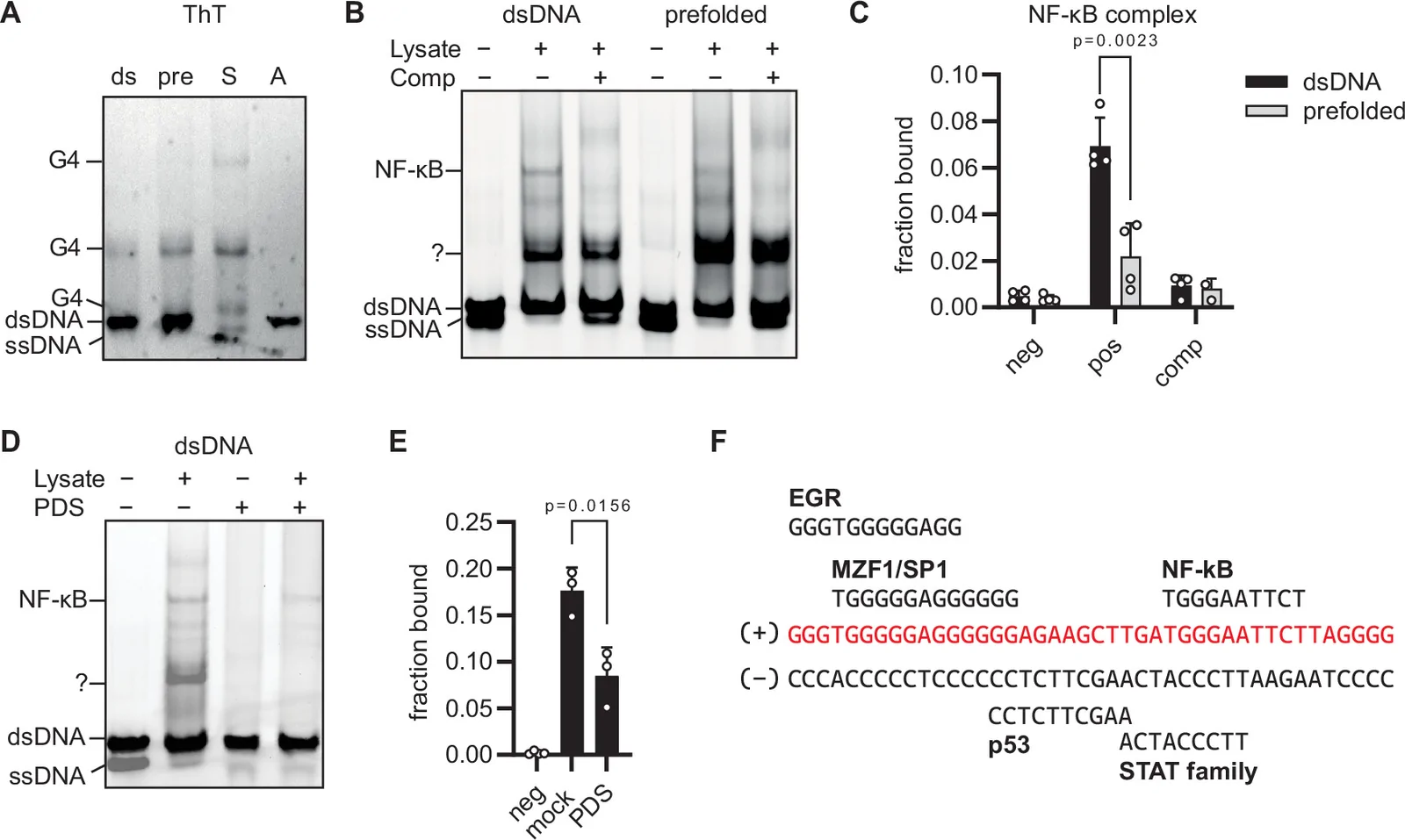
Molecular mechanism of G4-dependent NF-κB inhibition. **A** ThT analysis of G4-formation of different oligo compositions. ds: double-stranded, pre: prefolded, S: sense, A: antisense. **B** Gel shift assay using the G4 sequence, including short flanking regions, either folded as a full duplex (dsDNA), or separately (prefolded), to allow folding of the G4, prior to annealing with the negative strand. A visual overview is given in Supplementary Fig. 5A. **C** Quantification of NF-κB complex formation in (**B**) (*n* = 4). Band intensities were normalized to total lane intensity. **D** Gel shift assay using the *Il1b* G4 sequence, either annealed in the presence or absence of 25 µM PDS to favor G4-folding. All gel shift experiments were performed in three independent biological replicates. A representative image is shown. **E** Quantification of NF-κB complex formation in D (*n* = 3). Band intensities were normalized to the total intensity of dsDNA (bands with Cy3 and Cy5 signal). **F** In silico predicted TF binding sites in the G4-forming region. All gel shift experiments were performed in at least three independent biological replicates. A representative image is shown. Quantification data are represented as mean ± S.D. Statistical significance (two-tailed Student’s *t*-test) is represented by the *p*-value. Source data are provided as a Source Data file.

After confirming that G4-stabilization physically reduces NF-κB occupancy, we investigated the molecular mechanism in vitro using electrophoretic mobility shift assays (EMSA). Under normal conditions, the binding cleft of the NF-κB dimer tightly surrounds the double-stranded DNA (dsDNA) target. Consequently, we hypothesized that distortion of the double helix by a G4 may introduce a steric block within the binding site, thereby restricting the recruitment of the active TF. To determine whether such a mechanism may be responsible for the observed effects of PDS in blunting the immune response, in vitro binding assays were performed using 5′-Cy3/5-labeled oligos containing the G4 and the reverse complement sequence. To mimic either the canonical double helix or the partially single-stranded transcription bubble, the oligos were either annealed directly together to favor a double-stranded conformation (dsDNA) or the G-rich strand was pre-folded separately and combined at room temperature to preserve a G4-containing fold (prefolded, Supplementary Fig. 5A). To verify if the folding strategies yielded the desired conformers, we tested G4-formation by in-gel Thioflavin T (ThT) staining (Fig. 5A). This analysis revealed the presence of a single major band for the concomitantly folded oligos. This is likely attributable to background staining by ThT, as even the negative strand without any G-tracts showed a clear signal (Fig. 5A). The positive strand containing the G4 exhibited a robust signal split between four distinct bands, likely representing the linear form and three different G4-containing conformers/oligomers. The pre-folded oligos exhibited an intermediate pattern, forming both conformations but with reduced intensity. As anticipated, the antisense strand exhibited no specific G4 signal in isolation (Fig. 5A). Next, we used competition assays to assess NF-κB binding to these oligos. As a positive control a non-G4-forming dsDNA fragment harboring a consensus NF-κB target oligo was used, showing a strong, competitor-sensitive shift (Supplementary Fig. 5B, C). Expectedly, no clear, NF-κB-specific binding was observed with the single-stranded oligos (Supplementary Fig. 5D). In line with our hypothesis, the EMSA data demonstrated that the fully annealed dsDNA motif exhibited robust NF-κB recruitment, as shown by the appearance of a distinct band sensitive to competitor treatment. In contrast, the prefolded G4-containing sequences only showed a weaker band at the corresponding size (Fig. 5B, C). Importantly, when denatured target oligos were annealed in the presence of PDS, favoring G4-containing conformations over dsDNA, the NF-κB-dependent shift was markedly reduced (Fig. 5D, E and Supplementary Fig. 5A).

We also found that there is at least one other complex that forms on the investigated sequence, which was resistant to NF-κB competitor treatment (Fig. 5B and Supplementary Fig. 5E). Moreover, this complex seems to be unaffected by G4 formation, as the normalized band intensity was unaltered between the dsDNA and pre-folded template, in contrast to the NF-κB band (Fig. 5B and Supplementary Fig. 5E). This argues for a potentially competitive recruitment of TFs, regulated by G4 formation. Thus, we investigated whether other predicted TF-binding sites are present within the core G4 motif. Indeed, we found that EGR, MZF1, SP1, p53 and members of the STAT family potentially bind to sequences contained in the G4 oligo (Fig. 5E). Interestingly, the G4-containing single strand seems to be sufficient to form this complex, whereas the negative strand is not (Supplementary Fig. 5C), making EGR, MZF1 and SP1 the most likely candidates for such a mechanism, SP1 being the top candidate as it was already described as avid G4 binder^20,52^. In line with previous observations that G4 ligands can actively compete with G4-binding proteins, we found that this complex is also highly sensitive to PDS treatment (Fig. 5D).

Overall, our study reveals that structural defects induced by G4-folding can physically hinder NF-κB association with its target sequences, potentially altering TF complexes at relevant immune genes and leading to severe defects in mounting an inflammatory response.

## Discussion

Given their pivotal role in orchestrating immune responses, macrophages are a clinically relevant cell type for studying fundamental regulatory mechanisms. Macrophage activation relies on highly dynamic transcriptional programs that enable rapid adaptation to diverse environmental stimuli^41,53^. Upon sensing DAMPs or PAMPs such as LPS, signaling cascades converge on key transcription factors, such as NF-κB, a master regulator orchestrating the expression of pro-inflammatory effectors, cytokines, and chemokines^6,54,55^. The exact transcriptional reprogramming determines whether macrophages perform pro-inflammatory, homeostatic, or immunosuppressive functions^56^. Consequently, precise modulation of promoter accessibility and TF occupancy is essential to balance effective host defense and prevention of excessive inflammation^6^.

Beyond TF phosphorylation and translocation to the nucleus, previous studies have revealed that dynamic DNA secondary structures such as G4s represent another important layer of gene regulation^19,20,25,57–60^. G4s are overrepresented in promoter regions and can either promote or inhibit transcription depending on their folding state, stability, and interaction with specific proteins^61–63^. For instance, G4 stabilization has been shown to affect transcription of oncogenes such as *MYC* or *KIT*^64,65^, while in other cases G4s serve as recruitment hubs for transcription factors that modulate cell-type specific gene expression^25,66^. Moreover, G4-dependent reprogramming has been implicated in tumor immunomodulation and inflammatory signaling^34,67^, yet its function within innate immune cells in these settings, particularly macrophages, has remained largely unexplored.

Our study identifies G4 formation as a direct and adjustable modulator of transcriptional activity in macrophages. By combining treatment with the G4-stabilizing ligand PDS and bacterial LPS stimulation, we demonstrate that G4 stabilization markedly controls the inflammatory response. Importantly, this suppression occurs without compromising essential cellular processes such as phagocytosis or mitochondrial metabolism, indicating that PDS treatment acts specifically at the transcriptional level (Fig. 1 and Supplementary Fig. 1).

Transcriptomic and functional analyses revealed that G4 stabilization preferentially interferes with pro-inflammatory NF-κB-dependent gene networks (Fig. 2). While canonical upstream signaling in response to LPS via TLR4 and IκB phosphorylation remained largely unaffected, transcription of NF-κB target genes, including *Il1a*, *Il1b*, *Il6*, and *Cd40*, was markedly reduced (Figs. 1 and 2). In contrast, several LPS-responsive genes, which were not affected by PDS cotreatment, were found to be involved in counter-regulatory pathways (Supplementary Fig. 2). Reporter assays confirmed that G4 formation only inhibits transcriptional activation, but not the initial phosphorylation of NF-κB p65 and RELB (Fig. 2 and Supplementary Fig. 2). This transcriptional blockade reduced the secretion of key cytokines and the autocrine feedback loops that normally amplify inflammatory signaling^68^ (Figs. 2 and 3). It is important to note that the mechanism reported here applies to early inflammatory phases in the absence of DNA damage. Under conditions of prolonged stress or elevated DNA damage, increased G4 abundance may engage additional pathways, such as cGAS-STING activation, which have been reported to enhance inflammatory processes^34,35,69^.

Mechanistically, chromatin analyses provide evidence that stabilized G4s hinder NF-κB binding at several target promoters. Interestingly, at least 20% of NF-κB targets within open chromatin regions after LPS treatment harbor potential G4s (Supplementary Fig. 4), including the central cytokine IL1B. A combination of ATAC-seq and ChIP-qPCR revealed reduced NF-κB occupancy at genomic loci harboring G4 motifs within the *Il1b* promoter region^50,68^ (Fig. 4). In vitro binding assays further confirmed that prefolded G4 structures prevent efficient NF-κB association with its cognate DNA sequence. Importantly, the inclusion of PDS during the folding process significantly reduced NF-κB-DNA complex formation, supporting a model in which G4 folding sterically competes with transcription factor binding. G4 pull-down assays revealed that LPS stimulation alone transiently increases G4 folding due to continuous DNA melting within the active transcription bubble, linking this molecular mechanism to the dynamic in vivo chromatin context. Stabilization of these transient structures by PDS creates a severe transcriptional roadblock, ultimately causing the local chromatin architecture to revert to a transcriptionally dormant state (Fig. 4). Future global G4 profiling will be critical to determine if this mechanism represents a genome-wide regulatory principle. Notably, other transcription factors such as EGR, SP1, and MZF1 are predicted to recognize the same G-rich regions, suggesting that presence or absence of a G4 could also dynamically restructure the composition of promoter-bound complexes, thereby tuning gene expression in a context-dependent manner (Fig. 5). Along these lines, the reported enhancement of the anti-inflammatory lipid-transporter ABCA1 by the TF NONO via a promoter G4 motif ^39^ exemplifies that G4 structures may mediate an immunosuppressive state in macrophages not only by blocking inflammatory gene expression, but also via TF recruitment to anti-inflammatory loci.

In clinical contexts, targeting G4s in cancer cells is discussed as promising approach for antitumor therapy^40,70^. Our data suggest additional potential for various diseases, as G4-mediated attenuation of NF-κB signaling could act as a checkpoint regulating subsets of inflammatory genes in immune cells, in contrast to general NF-κB inhibition^71^. These insights corroborate previous findings that synthetic G4-forming oligonucleotides can alleviate sepsis in mice by suppressing inflammatory cytokine release^72^, while G4-dependent transcriptional programs in tumor cells promote remodeling of the tumor microenvironment^73^.

Taken together, our data suggest a novel model in which G4 stabilization can serve as a localized structural checkpoint that limits NF-κB-driven transcription in macrophages. This is corroborated by the back-to-back submission of Maione et al., in which similar effects were reported using an in vivo model of sterile inflammation in mice, treated with the G4-stabilizer PhenDC3. Their work elegantly demonstrates that pharmacological changes to G4 stability can suppress excessive inflammatory responses by selectively targeting NF-κB-dependent signaling. Consequently, neutrophil and macrophage invasion to sites of inflammation is reduced, whereas the number of circulating immune cells remains unchanged. Hence, pharmaceutical exploitation of this mechanism may have the potential to reduce overshooting cytokine production in clinical manifestations of sterile inflammation, such as autoimmune diseases, while avoiding general immunosuppressive effects. By demonstrating that DNA structure can directly modulate the inflammatory transcriptome, this study expands the understanding of transcriptional regulation in immunity and identifies G4 formation as a pivotal determinant of macrophage plasticity.

## Methods

### Cell lines and cell culture

Bone marrow-derived mouse macrophages were immortalized using the J2 retrovirus, according to the protocol established by Roberson and Walker (1988)^74^. The growth conditions for iBMDMs (kindly provided by Zeinab Abdullah) were Dulbecco´s modified Eagle´s medium (DMEM) (Thermo Fisher Scientific, #10566016) supplemented with 10% (v/v) fetal bovine serum (FBS) (Thermo Fisher Scientific, #10500064) and 100 U/ml penicillin-streptomycin (Thermo Fisher Scientific, #15140122); the growth conditions for the RAW-Blue NF-κB SEAP reporter cell line (Invivogen, #raw-sp) were the same and in addition 100 µg/ml zeocin (InvivoGen, #ant-zn-05), and 100 µg/ml normocin (InvivoGen, #ant-nr-05).

If not indicated otherwise, the cells were treated with 25 µM PDS (Selleckchem, #S7444) and 200 ng/ml LPS (Sigma, #L7770) or H_2_O_2_ (30% (w/w) Sigma, #H1009) for 4 h or with 10 µM Nigericin (InvivoGen, #tlrl-nig-5) for 1 h at 37 °C with 5% CO_2_.

### AlamarBlue assay

The metabolic activity was determined using the alamarBlue Cell Viability Assay Reagent (VWR, #GENO786-921). 60,000 cells were seeded in a black bottom clear 96-well plate and 10 µl of 10% alamarBlue solution was added (final volume 100 µl per well) and incubated for 3 h at 37 °C. The emission at 590 nm was measured using a Fluorskan ^TM^ FL plate reader (Thermo Fisher Scientific).

### Western blot analysis

Cells were treated with PDS, LPS or both for 4 h. Nigericin was added for 1 h to provide the second signal for full inflammasome activation. For detection of secreted proteins, 1 ml of supernatant was precipitated with trichloroacetic acid, washed with acetone, and the pellet resuspended in 30 µl Lämmli buffer. The protein lysates were separated by 10% SDS-PAGEs and blotted on a Protran BA83 Nitrocellulose membrane (GE Healthcare). Blocking was performed with 5% (w/v) BSA (AppliChem, #A6588) in TBS-T and incubated with the primary antibodies overnight at 4 °C with agitation. The membranes were washed three times for 5 min with TBS-T, incubated with appropriate fluorescent-coupled secondary antibodies for 1 h at RT and followed by three washing steps. Reactions were detected using the Molecular Imager ChemiDOC XRS Imaging system (Bio-Rad) with automatically calculated detection times by the ChemiDOC software. ImageJ (Fiji^75^) was used to determine the protein intensities.

#### Used antibodies

Phospho p65 (1:1,000 Cell Signaling, #9936); Donkey anti-rabbit 800 CW (1:10,000 LI-COR, #926-32213); Donkey anti-mouse 800 CW (1:10,000 LI-COR, #926-32212); p65 (1:300 Santa Cruz, #sc8008); Phospho-histone H2A.X (1:1,000 Cell Signaling, #9718); GAPDH (1:1,000 Santa Cruz, #sc69778); histone H3 (1:1,000 Abcam, #Ab1791); IL-1β (1:1,000 Proteintech, #26048-1-AP); RelB (1:1,000 Proteintech, #25027-1-AP); Phospho-RelB (1:1,000 Cell Signaling, #5025 T); p105/p50 (1:1,000 Proteintech, #14220-1-AP); Phospho-p50 (1:200 Santa Cruz, #sc-271908); Caspase-1 (1:1,000 Adipogen, #AG-20B-0042-C100); NLRP3 (1:5,000 Proteintech, #30109-1-AP)

### Detection of G4 with BG4 antibody

Immunofluorescence staining of G-quadruplex (G4) structures was performed as previously described^76^. Briefly, immortalized bone marrow-derived macrophages (iBMDMs) were seeded onto glass coverslips in 24-well plates at a density of 7 × 10^4^ cells per well. Twenty-four hours post-seeding, cells were treated with PDS for 4 h. To preserve G4 structures, cells were pre-fixed using a 1:1 mixture of culture medium (DMEM) and methanol/acetic acid (3:1) for 5 min at room temperature. Cells were then incubated in methanol/acetic acid (3:1) for 10 min at room temperature. The fixed cells were permeabilized with 0.1% (v/v) Triton X-100 in PBS for 3 min. Samples were blocked by incubating the coverslips in blocking solution (2% (w/v) nonfat dry milk (Carl Roth, #T145) in PBS, pH 7.4) for 1 h at room temperature. For specific G4 detection, samples were incubated with 2 µg of the FLAG-tagged BG4 antibody per slide in blocking solution for 2 h at room temperature. The primary detection was performed using a rabbit anti-DYKDDDDK epitope antibody (1:800; Cell Signaling Technology, #2368) for 1 h, followed by incubation with a Cyanine 3-conjugated goat anti-rabbit IgG secondary antibody (1:1000; Life Technologies, #A10520) for 1 h at room temperature under gentle rocking. After each incubation step, the cells were washed three times for 10 min with 0.1% (v/v) Tween-20 (Carl Roth, #9127) in PBS. Coverslips were mounted onto glass slides using Fluoroshield mounting medium containing DAPI (Merck, #F6057) for nuclear counterstaining.

### BG-flow

BG-Flow was performed as described in DeMagis et al.^76^. After trypsinization, cells were harvested by centrifugation. Cell pellets were resuspended in 1 ml of a 1:1 mixture of DMEM and methanol/acetic acid (3:1) for 5 min at room temperature (RT). After centrifugation at 300 g for 5 min at RT, cells were fixed in 3:1 (v/v) methanol/acetic acid for 10 min at RT, and then for 15 min at RT. Following two PBS washes, cells were permeabilized with 0.1% (v/v) Triton X-100 in PBS for 5 min at RT and washed twice with PBS. Cells were blocked using 2% (w/v) nonfat dry milk in PBS for 45 min at RT. For target detection, cells were sequentially incubated at RT on a tube rotator (30 rpm) with 5 μg BG4 in blocking buffer for 2 h, followed by a rabbit anti-FLAG antibody (Cell Signaling, #2368; 1:250) for 1 h, and an Alexa Fluor 488 secondary antibody (Invitrogen, #A11008; 1:600) for 1 h. Between antibody incubations, cells were washed twice with 0.1% (v/v) Tween in PBS. After the final incubation, cells were washed once with 0.1% Tween in PBS and once with standard PBS. Where indicated, DNA was co-stained using 10 μg/ml DAPI (alternatively 50 μg/ml PI or 1.2 μg/ml Hoechst33258) and 50 μg/ml RNase A (Ambion, #AM2271) in PBS for 30 min at 37 °C. Finally, cells were resuspended in 1 ml PBS and analyzed on a FACSCanto II Cell Analyzer (BD Biosciences). Data was processed using FlowJo (BD Biosciences), gating populations by forward scatter (FSC) and side scatter (SSC). Samples omitting the BG4 primary antibody served as negative controls.

### CD40 and CD80 expression analysis and mitochondrial fitness

The iBMDMs were collected, washed and resuspended in 50 µl antibody mix 1) MTG + MTDR (Fc-block, 1:100, Invitrogen, #14-0161-86; MTG-FITC, 1:4,000, Invitrogen, #M7514; MTDR-APC, 1:4,000, Invitrogen, #M22426; F4/80 BV421, 1:200, #Biolegend 123132; L/D Near-IR, 1:800, Invitrogen, #L34976A); 2) MTG+MitoSox (Fc-block 1:100; MTG-FITC 1:4,000; MitoSox PE, 1:4,000, Invitrogen, #M36008, F4/80, 1:200; L/D, 1:800); 3) (Fc-block, 1:100; CD40 PE, 1:200, eBioScience, #12-0401-83; CD80 AF647, 1:200, Biolegend, #104718; F4/80, 1:200; L/D, 1:1,000) and proceeded for flow cytometry analysis using a FACS Canto I. Appropriate single stains and FMOs were included. FlowJo software was used for analysis.

### RNA isolation

The RNA was isolated using TRIzol (Thermo Fisher Scientific, #15596026) according to the manufacturer´s instructions.

### RNA sequencing

The 3´RNA sequencing was performed at the NGS core facility Bonn at HiSeq 2500 V4 (10 M raw reads; 1×150 bp). The analysis was performed using the Partek Flow software (Partek Inc.). Sequencing reads were aligned to mm10 using the STAR aligner.

### Standard quantitative PCR

For reverse transcription, the QuantiTect Reverse Transcription Kit (Qiagen, #205311) was used according to the manufacturer’s instructions. The quantification of cDNA was performed using the iQ SYBR Green Supermix (Bio-Rad, #170-8880) on a CFX96 Real Time System with a C1000 Touch Thermal Cycler (Bio-Rad) with the primers in Table 1.

**Table 1:** qPCR primers used for *Il1b* cDNA quantification

Name	Sequence
S_IL1B	TGGACCTTCCAGGATGAGGACA
A_IL1B	GTTCATCTCGGAGCCTGTAGTG
S_GAPDH	CATCACTGCCACCCAGAAGACTG
A_GAPDH	ATGCCAGTGAGCTTCCCGTTCAG

The expression changes were calculated based on the 2^(-ΔΔCt) method using GAPDH for normalization.

### Phagocytosis uptake assay with *Salmonella*

1×10^6^ iBMDMs were mixed with 50 µl/ml normal mouse serum and incubated for 15 min at 37 °C. 2×10^8^ CFU/ml mCherry-labeled heat-inactivated *Salmonella* (provided by the Abdullah lab) were incubated for 2 h at 37 °C under rotation. The cells were collected, washed with PBS and stained for L/D (1:800; Near-IR, Invitrogen, #L34976A), CD11b (1:400; FITC, Pharmingen, #553310), F4/80 (1:400; BV421, #Biolegend 123132), Fc Block (1:100, Invitrogen, #14-0161-86) and incubated for 30 min at 4 °C. The samples were washed with PBS and proceeded for flow cytometry analysis using a Fortessa II (BD Biosciences). Appropriate single stains and controls without bacteria were included. FlowJo software was used for analysis.

### Phagocytosis killing assay with *Listeria*

500,000 iBMDMs were seeded (DMEM without antibiotics), if indicated, treated for 20 h with 100 ng/ml recombinant mouse INFγ and infected with 50 µl *Listeria* (MOI 5). After 30 min the medium was replaced with medium containing gentamycin (100 µg/ml) and after 1 h (t = 0) and 4 h (t = 1) the cells were washed twice with PBS and lysed with 500 µl lysis buffer (0.01% (v/v) Triton X-100 in ddH_2_O). Serial dilutions (1:10 dilution was diluted 5 times) with the lysate were performed and plated on BHI plates (20 µl were plated). The next morning, the colonies were counted, and the CFU/ml was calculated.

### HTRF IL1B

The supernatants of triplicates were collected and the assay performed according to the manufacturer’s instructions (Cisbio, #62MIL1BPEG). In short: 8 µl sample, 8 µl medium, 4 µl antibody mix and 16 µl standard were used, and a SpectraMax M5e plate reader (Molecular Devices) was used (665 and 620 nm).

### QUANTI-Blue assay with RAW NF-κB cells (InvivoGen)

The assay was performed based on the manufacturer´s instructions. In brief, 100,000 cells per well were seeded in a 96-well plate. 190 µl QUANTI-Blue^TM^ Solution (InvivoGen, #rep-qbs) were incubated with 10 µl treated RAW-Blue cell supernatant and incubated at 37 °C for 6 h. The SEAP levels were measured using a SpectaMax M5e spectrophotometer at 630 nm.

### Procartaplex

The supernatants of the iBMDMs were collected in triplicate. The Procartaplex 9 Plex (Thermo Scientific, #D4445144) was used according to the manufacturer´s instructions. The concentrations were calculated using Thermo Scientific software based on standard curves and measuring the Mean Fluorescence Intensity (MFI).

### Inflammasome activation measurement via hASC-mCerulean

Immortalized mouse macrophages with hASC-mCerulean reporter were used for this assay. The well imaging plate (Greiner bio, #6055300) was coated with 0.01% Poly-L-Lysine solution (Sigma-Aldrich, #P8920-100ML) for 15 min at 37 °C and washed three times with PBS. 50,000 (*n *= 1) and 30,000 (*n* = 2, 3) cells per well were seeded. The cells were treated with PDS and/or LPS and in the last hour with 10 µM nigericin (Invitrogen, #N1495). In the last 30 min of incubation 50 µM VX-765 solution (Selleckchem, #S2228) was added to all wells. The fixation and nuclear strain were performed in 4% PFA and DRAQ5 (Thermo Fisher Scientific, #62251) (dilution of 1:5000) for 15 min at RT in the dark. The plate was centrifuged at 340 g for min, the supernatant was removed and replaced with 200 µl PBS. For analysis, the automated imaging on the Cell Discoverer 7 (Carl Zeiss Microscopy) was used (imaging settings: 10 images per well, 6 z-stack planes spanning 10 µm with intervals of 2 µm; Objectives *n* = 1: Plan-Apochromat 20x/0.95 with 1x Tubulens (20x magnification); *n* = 2,3: Plan-Apochromat 5x/0.35 with 2x Tubulens (10x magnification). The maximum intensity projections from all planes in Zen 2.6 (blue edition, Carl Zeiss Microscopy) software were generated, and the bright field channel was removed. The new file was used for automated quantification in the CellProfiler (3.1.9) Software.

### ATAC sequencing

#### Sample preparation and sequencing

For ATAC-Seq, 50,000 cells per condition were washed with PBS and lysed in 50 µl of lysis buffer (10 mM Tris-HCl, pH 7.4, 10 mM NaCl, 3 mM MgCl_2_, 0.1% (v/v) NP-40). Following centrifugation (500 g for 10 min at 4 °C), the nuclear pellet was resuspended in a mix comprising 25 µl of 2x TD buffer, 2.5 µl of Tn5 transposase (Nextera DNA Library Kit, Illumina, #FC-121-1030), and 22.5 µl of nuclease-free H_2_O. The reaction was incubated for 30 min at 37 °C. DNA was subsequently purified using the MinElute Reaction Cleanup Kit (Qiagen, #28204) according to the manufacturer’s instructions and eluted in 10 µl of elution buffer. Sequencing was performed at the Cologne Center for Genomics on an Illumina platform, using 50 million 150-bp paired-end reads per sample.

#### Data processing and peak calling

Sequence read preprocessing and alignment were performed using the following pipeline. Raw reads were trimmed using Cutadapt (v1.18)^77^ and aligned to the mouse reference genome (mm10) using Bowtie2 (v2.4.5)^78^. Alignments were sorted with SAMtools (v1.14)^79^ and PCR duplicates were removed using Picard (v2.26.10) (https://github.com/broadinstitute/picard). Peak calling on the sorted and filtered BAM files was performed using MACS2 (v2.2.7.1)^80^. To ensure high reproducibility across replicates, consensus peaks for each condition were defined using the Irreproducible Discovery Rate (IDR) framework (v2.0.4.2)^81^. Differentially accessible regions between conditions were identified utilizing the DiffBind package (v3.10) (http://bioconductor.org/packages/release/bioc/html/DiffBind.html).

### Fast ChIP

Chromatin immunoprecipitation was performed using the FastChIP protocol established by Bomsztyk and colleagues^82^. Briefly, iBMDMs were plated at a density of 6–8 × 10^6^ cells in 10 cm dishes. Cells were washed and covered with 5 ml PBS before crosslinking with 1.42% formaldehyde (Carl Roth, #4980) for 15 min at room temperature. The reaction was quenched with 125 mM glycine for 5 min at room temperature, and cells were washed twice with cold PBS, scraped into 1 ml PBS, and pelleted at 2000 g for 5 min at 4 °C. Pellets were resuspended in 1 ml IP buffer (150 mM NaCl, 50 mM Tris-HCl, pH 7.5, 5 mM EDTA, 0.5% NP-40, and 1% Triton X-100, protease inhibitors), and lysed by pipetting. After centrifugation at 12,000 × *g* for 1 min at 4 °C, the pellet was washed once with 1 ml IP buffer and centrifuged again. For sonication, pellets were resuspended in 1 ml IP buffer per 10 million cells, split into 300 µl aliquots, and sheared using a Bioruptor (Diagenode) for five cycles of 30 s on/30 s off, yielding average fragment sizes of 300–600 bp. Lysates were clarified at 12,000 × *g* for 10 min at 4 °C, and chromatin aliquots equivalent to 2 million cells were incubated overnight at 4 °C with a 1:100 dilution of a rabbit anti-NF-κB p65 antibody (D14E12, Cell Signaling Technology, #8242S) or an equivalent amount of a rabbit IgG control (Thermo Fisher Scientific, #02-6102. The next day, samples were centrifuged at 12000 g for 10 min at 4 °C, and the supernatant was then incubated with 20 µl of pre-washed protein G-Dynabeads (Invitrogen, #10446293) for 45 min at 4 °C. Beads were washed five times with 1 ml cold IP buffer and resuspended in 100 µl Proteinase K buffer (100 mM Tris-HCl, pH 7.5, 150 mM NaCl, 12.5 mM EDTA, 2% SDS, and 5 mM DTT). Samples were treated with 1 µl of 20 mg/ml Proteinase K (Carl Roth, #3719) for 30 min at 55 °C, followed by boiling at 95 °C for 10-15 min to inactivate the enzyme and decrosslink the chromatin. DNA was precipitated with 300 µl ethanol and 15 µg GlycoBlue (Thermo Fisher Scientific, #AM9515) at −20 °C for at least 1 h, pelleted at 20000 g for 30 min at 4 °C, washed with 70% ethanol, and resuspended in 50 µl water. qPCR was performed with 2 µl of the eluted DNA with the iQ SYBR Green Supermix (BioRad) using the primers in Table 2.

**Table 2:** qPCR primers used for Fast ChIP

Name	Sequence
S_IL1B_1	TTTGTTCCGCACATCCTGAC
A_IL1B_1	TTCGCAAGTGTGTCATCGTG
S_IL1B_2	AAATGCCTGCCTCCATCTTG
A_IL1B_2	AAAGAGGAAGTCGGCAAAGC
S_Globin	TCCACTGTGACAAGCTGCAT
A_Globin	GCTCCCTAGAATCGCTTCCC

All data were normalized to the respective IgG control samples.

### Analysis of NF-κB binding sites and G4s in the *Il1b* promoter

We analyzed the DNA sequence for NF-κB binding sites using position weight matrices (PWMs) from the HOCOMOCO v11 (https://hocomoco14.autosome.org/) database (RELA, RELB, REL, NFΚB1, NFΚB2) and TFBIND. Both DNA strands were scanned, and for each subsequence, the raw PWM score was normalized to a relative score between 0 and 1 using the theoretical minimum and maximum possible scores for that PWM. Sites with a relative score ≥ 0.80 were retained. To avoid redundancy, predicted sites located within 5 bp of each other were merged by keeping the highest-scoring site in the cluster. G4s were predicted by G4RNA screener^83^ using the algorithms cGcG, G4hunter and G4NN with a window size of 30 and step size of 5. Running scores are reported for each step.

### G4-DNA precipitation (G4DP)

For pulldown of chromatin G4s, a modified variant of the G4DP method established by Feng et al. (2022)^51^ was employed. Briefly, iBMDMs were seeded at 6 to 8 million cells in 10 cm dishes. The following day, cells were washed, overlaid with 5 ml PBS, and crosslinked using 1% formaldehyde for 10 min at room temperature. Crosslinking was quenched with 125 mM glycine for 5 min at room temperature, followed by two cold PBS washes, scraping into 1 ml cold PBS, and pelleting at 2000 g for 5 min at 4 °C. Cell pellets were lysed in 1 ml cold nuclear isolation buffer (10 mM Tris-HCl pH 7.4, 10 mM NaCl, 3 mM MgCl2, 0.1% NP-40, 1 mM DTT, PMSF) by pipetting and 15 min incubation on ice. After centrifugation at 1000 g for 5 min at 4 °C, nuclear pellets were resuspended in 300 µl sonication buffer (10 mM Tris-HCl, pH 7.4, 5 mM EDTA, 150 mM KCl, 0.5% NP-40, 0.5% SDS, 0.5 mM DTT, PMSF). Chromatin was sheared using a Bioruptor for 10 cycles of 30 s on/30 s off to generate fragments of 100-300 bp, clarified at 12000 g for 10 min at 4 °C, and diluted with 1.2 ml incubation buffer to reduce the SDS concentration below 0.1%. The lysate was treated with 15 µl of 10 mg/ml RNase A for 30 min at 37 °C with shaking. 10% of the lysate was reserved as the input sample. For the pulldown, chromatin aliquots equivalent to ~2 million cells were incubated overnight at 4 °C with 100 µM BioCyTASQ (Merck, #SCT246). Streptavidin Dynabeads M280 (Invitrogen, #10202983) were washed with incubation buffer, added to the sample, and rotated for 4 h at 4 °C. To reduce genomic background, beads were washed twice with 1× rCutSmart buffer (NEB, #B6004S), resuspended in 100 µl of the same buffer with 2 U double-strand-specific dsDNase (Sigma Aldrich, #EN771), and incubated for 10 min at 37 °C with shaking, followed by three washes with cold incubation buffer. DNA was eluted twice using 200 µl elution buffer containing 100 mM NaHCO_3_ and 1% SDS at 65 °C for 15 min, pooled, supplemented with 16 µl 5 M NaCl, and decrosslinked for 4 h to overnight at 65 °C. DNA was extracted using phenol-chloroform-isoamyl alcohol, precipitated overnight at −20 °C with 15 µg glycoblue and 1 ml ethanol, washed with 70% ethanol until SDS was dissolved, and eluted in 100 µl water. qPCR was performed with 2 µl of the eluted DNA with the iQ SYBR Green Supermix (BioRad) using the primers in Table 3.

**Table 3:** qPCR primers used for G4DP

Name	Sequence
S_G4_IL1B_1	CCAACTTGTTTTCCCTCCCTTGT
A_G4_IL1B_1	TCTGCCACCCCTTGACTTCC
S_noG4_IL1B_1	AAGTGGGTGCTCTGGTTGCT
A_noG4_IL1B_1	GGGACTCCTACAGATGCAATGGT
S_IFNB_ISRE_qPCR	GCACATCAATGGGCCATAG
A_IFNB_ISRE_qPCR	CCACTCACAACCCTTCCTTC

All data were normalized to the respective input sample and globin levels, as an internal control.

### Generation of nuclear lysates from iBMDMs

Nuclear extracts were prepared utilizing an optimized rapid fractionation protocol^84^. Briefly, 5 × 10^6^ cells were harvested, washed with 1 ml of ice-cold PBS, and pelleted. The cell pellet was resuspended in 5 packed cell volumes of hypo-osmotic lysis buffer (0.3 M sucrose, 2% Tween-40, 10 mM HEPES-KOH pH 7.9, 10 mM KCl, 1.5 mM MgCl_2_, 0.1 mM EDTA, 0.5 mM DTT, protease inhibitors). To release intact nuclei, the suspension was subjected to mechanical disruption via repeated pipetting ( ~ 100 times). The resulting lysate was carefully layered over a 1.5 M sucrose cushion (1.5 M sucrose, 10 mM HEPES-KOH pH 7.9, 10 mM KCl, 1.5 mM MgCl_2_, 0.1 mM EDTA, 0.5 mM DTT, and protease inhibitors) and centrifuged for 10 min to isolate the nuclear fraction. The nuclear pellet was washed with low salt wash buffer (10 mM HEPES-KOH pH 7.9, 10 mM KCl, 1.5 mM MgCl_2_, 0.1 mM EDTA, 0.5 mM DTT, and protease inhibitors) and subsequently extracted in 100 µl of high salt extraction buffer (20 mM HEPES-KOH pH 7.9, 420 mM NaCl, 1.5 mM MgCl_2_, 0.2 mM EDTA, 25% glycerol, 0.5 mM DTT, and protease inhibitors) for 20 min on ice with intermittent gentle vortexing. The extract was cleared via centrifugation, and the supernatant containing high-purity nuclear proteins was collected, aliquoted, and stored at −80 °C.

### Electrophoretic mobility shift assay (EMSA)

#### Oligonucleotide hybridization and G-quadruplex folding

Fluorescently labeled oligonucleotides were designed to contain a central G-quadruplex (G4)-forming motif flanked by duplex-forming genomic sequences. To facilitate structured G4 folding within the flanking double-stranded context and to mimic the transient single-stranded state during transcriptional activation, a specialized denaturation-renaturation sequence was applied. Sense and antisense strands were diluted to a working concentration of 0.6 µM in binding buffer (10 mM Tris–HCl pH 7.4, 1 mM EDTA, 150 mM KCl, and 5% glycerol). The mixtures were denatured, flash-cooled on ice, and subsequently allowed to fold and anneal at 37 °C in the presence or absence of 25 µM PDS. Untreated control duplexes and single-stranded references were prepared under identical conditions. As a positive control, a double-stranded oligo containing an NF-κB consensus sequence (Cy5-GTTGAGGGGACTTTCCCAGGCA) that does not fold into a G4. This sequence was also used as an unlabeled competitor oligo to validate the NF-κB-dependency of shifted bands.

The sequences of the used oligos are shown in Table 4.

**Table 4:** Oligonucleotides used for EMSA

Name	Sequence
S_IL1B_G4 (Cy5)	AAACTGAAGGGTGGGGGAGGGGGGAGAAGCTTGATGGGAATTCTTAGGGGAAGAGGCT
A_IL1B_G4 (Cy3)	AGCCTCTTCCCCTAAGAATTCCCATCAAGCTTCTCCCCCCTCCCCCACCCTTCAGTTT

#### In vitro binding and electrophoretic separation

EMSA binding reactions were performed in a total volume of 18 µl. Each reaction mixture contained 1× binding buffer, 1 µg of poly(dI:dC) (Thermo Fisher Scientific, #20148E), and 1–2 µl of purified nuclear lysate. For competition controls or super-shift experiments, a 100-fold excess of unlabeled consensus oligonucleotide was included during a pre-incubation phase of 5 min at 20 °C. Then, 2 µl of the pre-folded fluorescent target oligonucleotide was added, followed by an additional incubation for 30 min at 20 °C. Protein-DNA complexes were resolved on a 5.5% native TBE polyacrylamide gel. The gel was pre-run on ice for at least 30 min at 120 V in 0.25x TBE running buffer. Samples were separated at 180 V for approximately 40 min until the bromophenol blue migration front reached the lower third of the gel. Fluorescent signals were visualized by direct gel imaging on a BioRad Chemidoc system. Quantification was performed on composite images (Cy3 + Cy5) using Fiji.

### Reporting summary

Further information on research design is available in the Nature Portfolio Reporting Summary linked to this article.

## Supplementary information

Supplementary Information

Description of Additional Supplementary Files

Supplementary Data 1

Reporting Summary

Transparent Peer Review file

## Source data

Source Data

## Data Availability

The high-throughput sequencing datasets generated in this study are available in NCBI’s Sequence Read Archive (SRA) under the accession codes PRJNA1489410 (ATAC-seq) and PRJNA1489405 (3′ RNA-seq). All remaining data supporting the findings in this paper are provided in the main manuscript and its Supplementary files. The cell lines and plasmids generated in this study are available from the corresponding author upon request. Source data are provided with this paper.
