# Supplementary material for: G-quadruplex structures affect the immune response to bacterial infection in mouse macrophages
Source: Nat Commun. 2026 Oct 20;17:10955. doi: 10.1038/s41467-026-78610-w (PMC13683368; doi:10.1038/s41467-026-78610-w)
Supplement: Supplementary file 1 [file 41467_2026_78610_MOESM1_ESM.pdf]

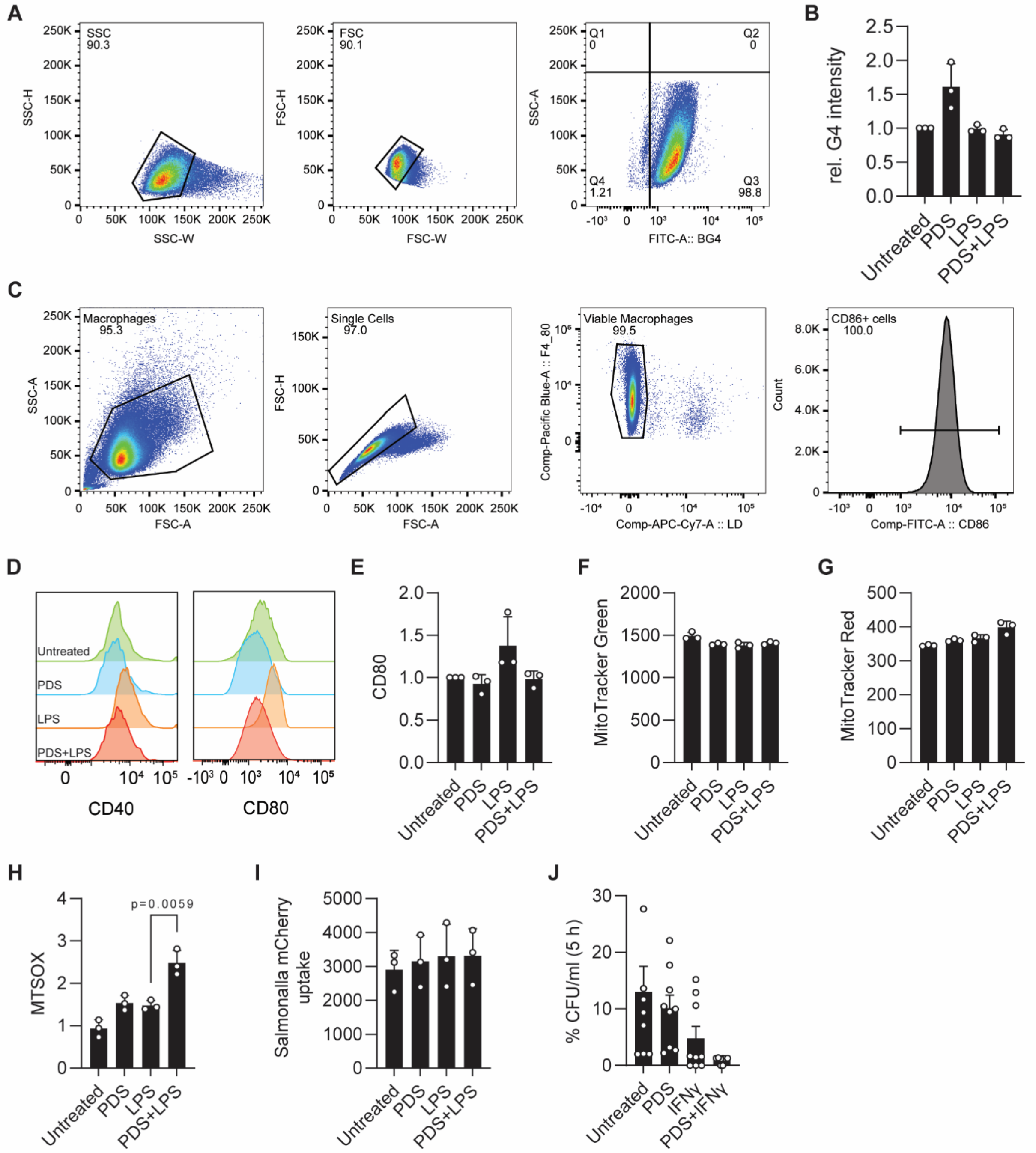

Supplementary Figure 1: Impact of PDS and LPS on macrophages. A. Gating of iBMDMs for BG-Flow analysis. Cells were gated for size (forward scatter, FSC) and granularity (side scatter, SSC), and BG4-dependent FITC signal was detected. **B.** BG-Flow measurement verifying the BG4 IF data in Figure 1F. **C.** Gating strategy for activation markers. From the Macrophage pool, single cells were selected, tested for viability using APC-Cy7 and finally tested for fluorescent signal representing CD40 or CD86. **D.** Representative histograms of FACS measurements of CD40 and CD80 surface expression. **E.** Quantification of B. **F-H.** Quantification of FACS measurement of mitochondrial mass (MitoTracker Green), activity (MitoTracker Red) and superoxide production (MTSox) upon PDS and LPS treatment. **I.** Macrophage bacterial uptake assay using mCherry-tagged *Salmonella*. **J.** Macrophage bacterial killing assay using *Legionella* colony formation as measurement 5h after incubation with macrophages. All experiments were performed in at least three independent biological replicates. Statistical significance (Two-tailed Student's t-test) is represented by the p-value. Source data are provided as a Source Data file.

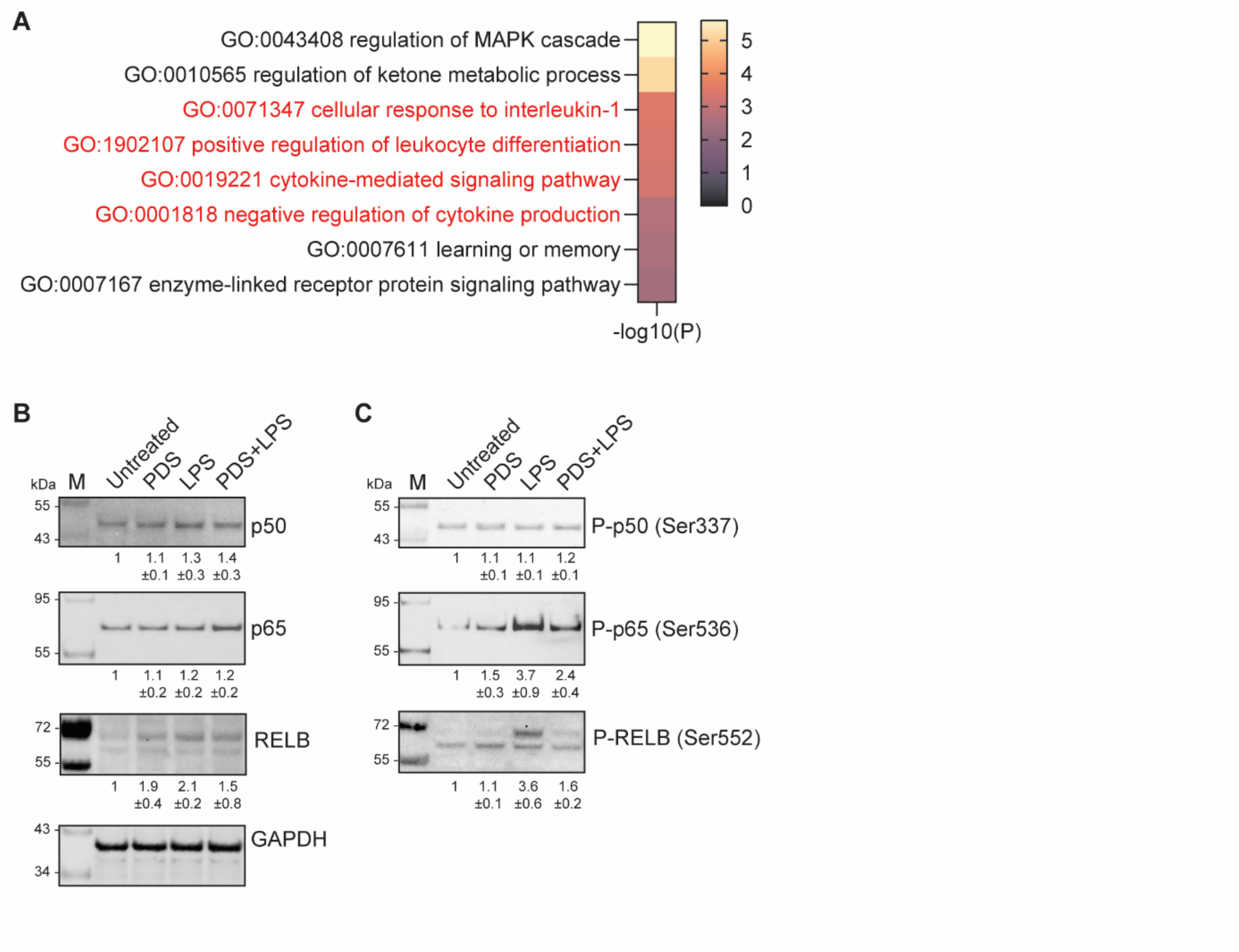

Supplementary Figure 2: Pathway analysis of PDS independent genes and western blots of additional NF-κB subunits. **A.** Metascape pathway enrichment analysis of LPS-induced genes ($log_2FC > 1.0$) lacking PDS suppression ($\Delta log_2FC > -0.3$) based on RNA-seq data (full gene list in Supplementary Table 1). Data are sorted by negative P-value ($-log_{10}(P)$). Pathways relevant for immune regulation are highlighted in red. **B.** Western blot analysis of total expression levels of NF-κB subunits after PDS, LPS and co-treatment. **C.** Phosphorylation status of the subunits shown in B. All bands were normalized to GAPDH. Relative quantification of the bands compared to untreated are shown below the respective western blots. Quantification data are represented as mean ± S.E.M. All experiments were performed in three independent biological replicates. Source data are provided as a Source Data file.

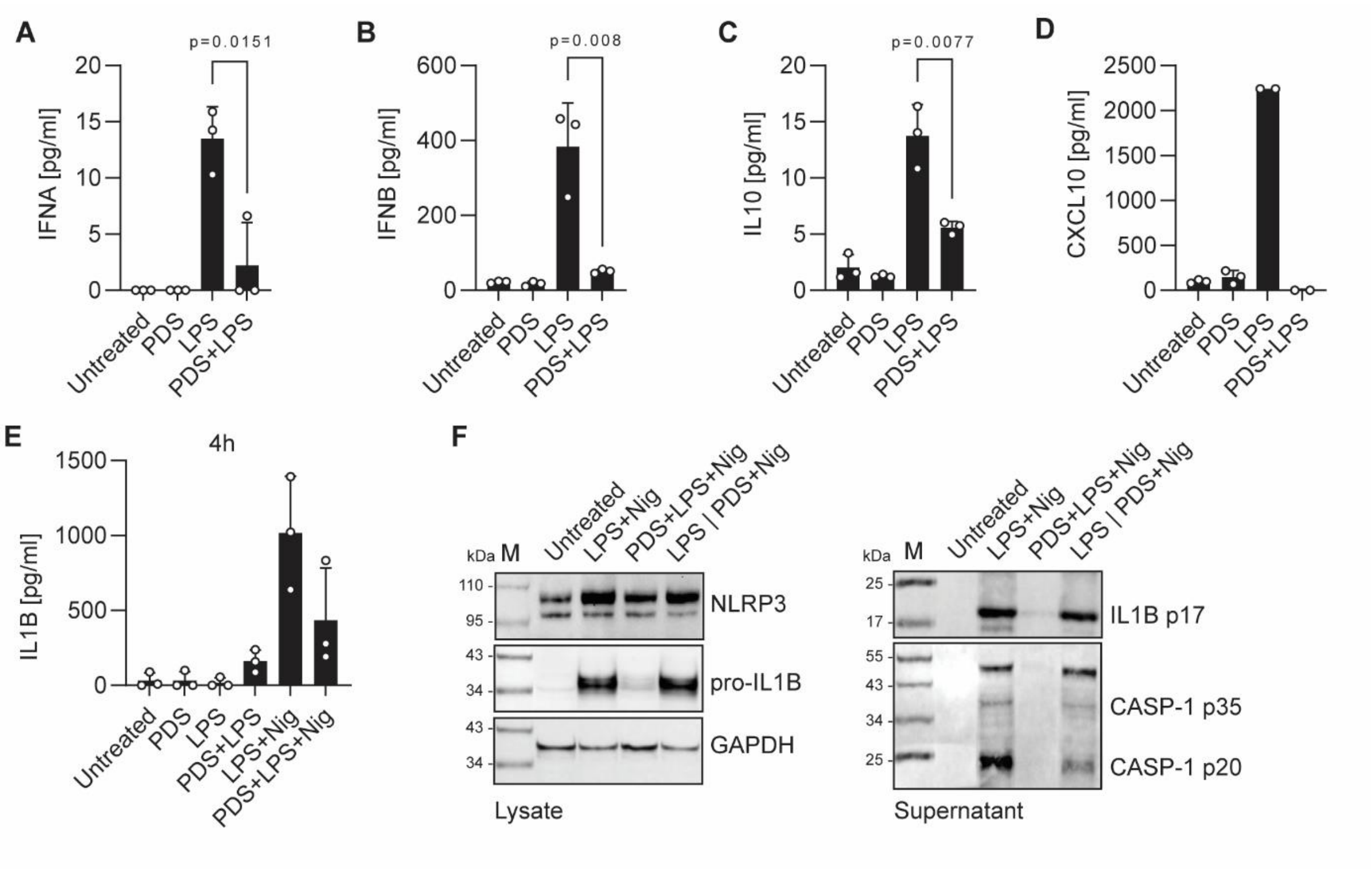

Supplementary Figure 3: Downstream effects of the PDS-dependent transcriptional block of NF-κB. A-D. ProcartaPlex quantification of extracellular cytokine concentrations (pg/ml) from iBMDM supernatants 4 h post-stimulation: **A.** IFNA, **B.** IFNB, **C.** IL10, and **D.** CXCL10. **E.** ProcartaPlex measurement of secreted IL1B as shown in Figure 3H, but 4h post stimulation. **F.** Western blot analysis of central components of inflammasome function under different treatments. "PDS+LPS+Nig" denotes continuous PDS treatment prior to nigericin addition. "LPS | PDS+Nig" denotes PDS addition exclusively during the 1 h nigericin activation phase. Data are represented as mean ± S.D. All experiments were performed in at least three independent biological replicates. Statistical significance (Two-tailed Student's t-test) is represented by the p-value. Source data are provided as a Source Data file.

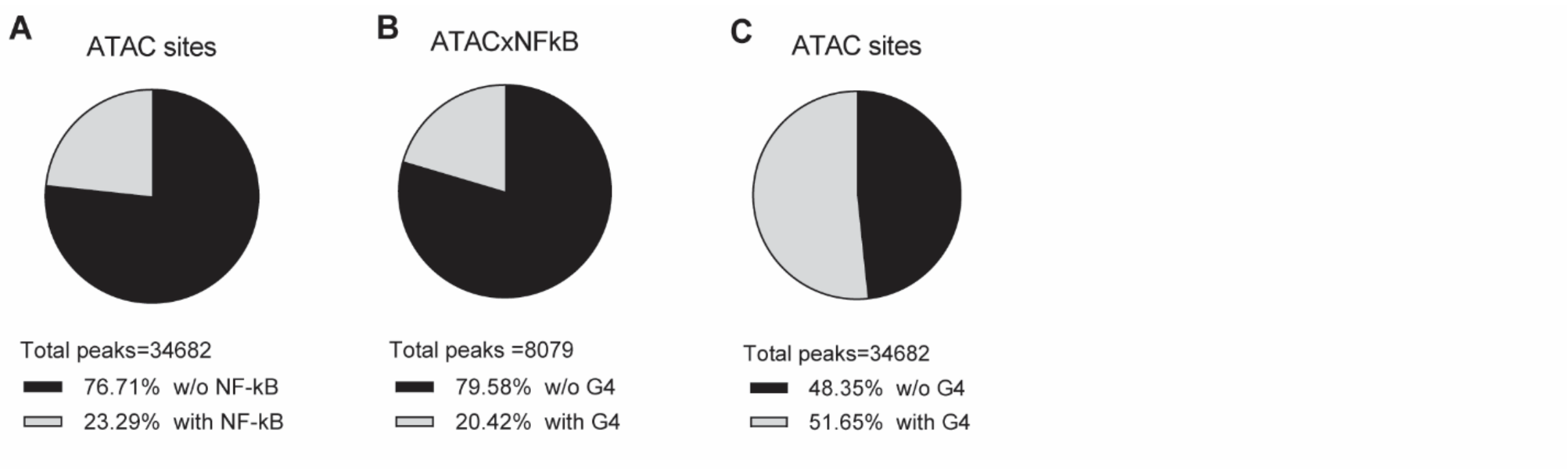

Supplementary Figure 4: G4-dependence of NF-κB binding to DNA. A. Overlap distribution between open chromatin regions identified via ATAC-seq and published NF-κB binding sites in macrophages[49]. **B.** Overlap analysis of ATAC-accessible NF-κB binding regions with experimentally mapped whole-genome G4 structures (G4-seq)[50]. **C.** Global overlap distribution between all ATAC-seq peaks and mapped G4 structures.

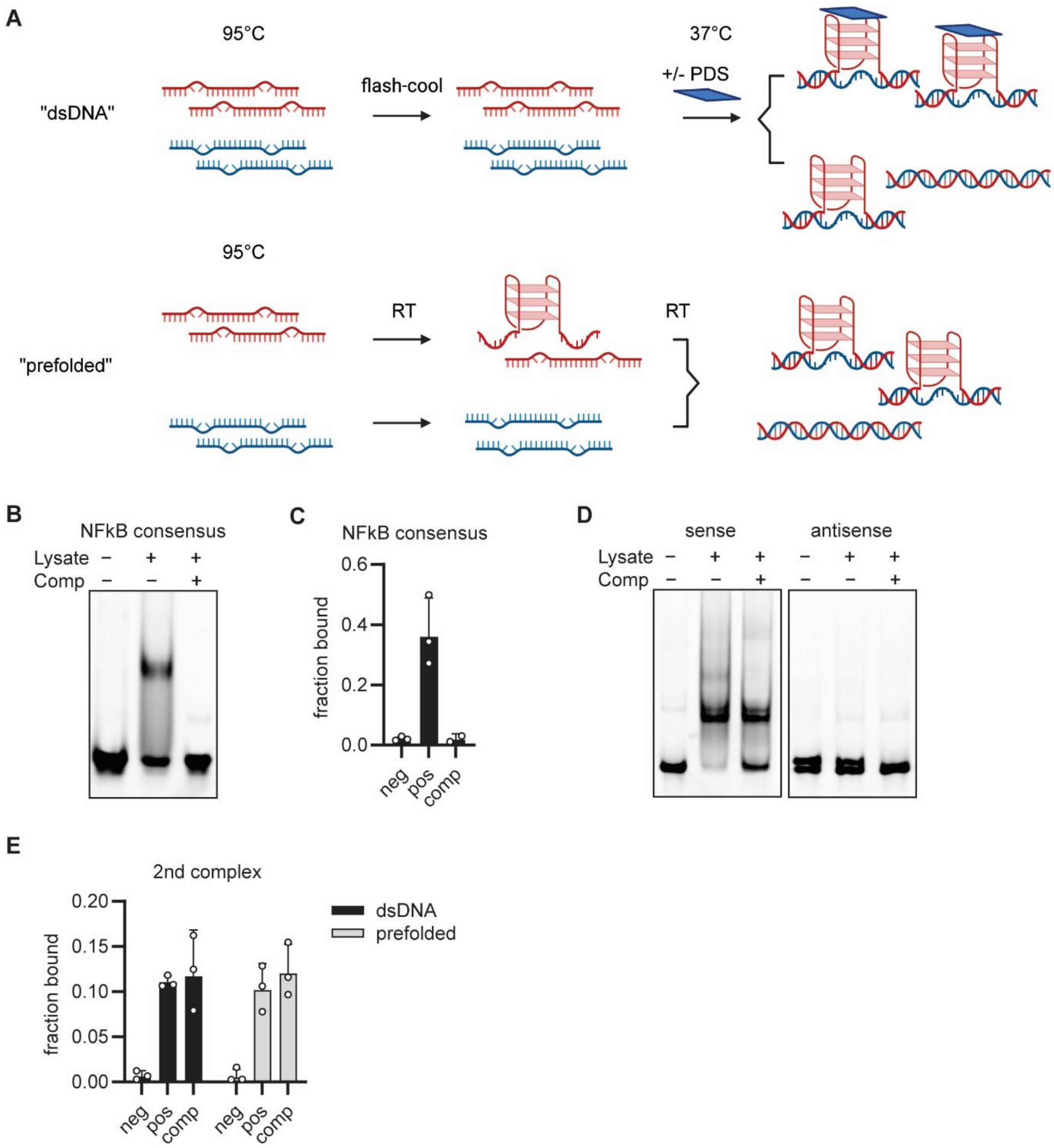

Supplementary Figure 5: Molecular mechanism of G4-dependent NF-κB inhibition. A. Schematic overview of oligonucleotide annealing procedures used for EMSA ("dsDNA", "prefolded", and "+PDS"). Created in BioRender. Paeschke, K. (2026) https://BioRender.com/le77shv. **B.** EMSA specificity control using a Cy5-labeled double-stranded NF-κB consensus probe and iBMDM nuclear lysate in the presence or absence of a 100-fold molar excess of unlabeled consensus competitor ("Comp"). **C.** Quantification of NF-κB binding from B. **D.** Control EMSA reactions using isolated single-stranded sense (left) or antisense (right) target oligonucleotides. **E.** Quantification of binding for the competitor-resistant secondary nucleoprotein complex observed in Figure 5B. Data are represented as mean ± S.D. All gel shift experiments were performed in three independent biological replicates. Source data are provided as a Source Data file.
