# Supplementary material for: G-quadruplex structures affect the immune response to bacterial infection in mouse macrophages
Source: Nat Commun. 2026 Oct 20;17:10955. doi: 10.1038/s41467-026-78610-w (PMC13683368; doi:10.1038/s41467-026-78610-w)
Supplement: Supplementary file 2 [file 41467_2026_78610_MOESM2_ESM.pdf]

Description of Additional Supplementary Files

File Name: Supplementary Data 1

Description: LPS-induced, but not PDS suppressed genes. Genes with a log2FC >1 (untreated vs. LPS) and a Δlog2FC of >-0.3 (LPS vs. PDS+LPS) were selected from the RNASeq dataset.
