# Supplementary material for: G-quadruplex structures affect the immune response to bacterial infection in mouse macrophages
Source: Nat Commun. 2026 Oct 20;17:10955. doi: 10.1038/s41467-026-78610-w (PMC13683368; doi:10.1038/s41467-026-78610-w)
Supplement: Supplementary file 3 [file 41467_2026_78610_MOESM4_ESM.pdf]

nature portfolio

Corresponding author(s): Katrin Paeschke

Last updated by author(s): Aug 23, 2026

Reporting Summary

Nature Portfolio wishes to improve the reproducibility of the work that we publish. This form provides structure for consistency and transparency in reporting. For further information on Nature Portfolio policies, see our Editorial Policies and the Editorial Policy Checklist.

Statistics

For all statistical analyses, confirm that the following items are present in the figure legend, table legend, main text, or Methods section.

n/a	Confirmed	
☐	☒	The exact sample size (*n*) for each experimental group/condition, given as a discrete number and unit of measurement
☐	☒	A statement on whether measurements were taken from distinct samples or whether the same sample was measured repeatedly
☐	☒	The statistical test(s) used AND whether they are one- or two-sided<br>*Only common tests should be described solely by name; describe more complex techniques in the Methods section.*
☐	☒	A description of all covariates tested
☒	☐	A description of any assumptions or corrections, such as tests of normality and adjustment for multiple comparisons
☐	☒	A full description of the statistical parameters including central tendency (e.g. means) or other basic estimates (e.g. regression coefficient) AND variation (e.g. standard deviation) or associated estimates of uncertainty (e.g. confidence intervals)
☐	☒	For null hypothesis testing, the test statistic (e.g. *F*, *t*, *r*) with confidence intervals, effect sizes, degrees of freedom and *P* value noted<br>*Give P values as exact values whenever suitable.*
☒	☐	For Bayesian analysis, information on the choice of priors and Markov chain Monte Carlo settings
☒	☐	For hierarchical and complex designs, identification of the appropriate level for tests and full reporting of outcomes
☒	☐	Estimates of effect sizes (e.g. Cohen's *d*, Pearson's *r*), indicating how they were calculated

Our web collection on statistics for biologists contains articles on many of the points above.

Software and code

Policy information about availability of computer code

Data collection	Flow cytometry data were acquired using BD FACSDiva software. Microplate-based assays (viability, reporter activity, and HTRF) were recorded using Thermo Scientific SkanIt and Molecular Devices SoftMax Pro software. qPCR data were collected via Bio-Rad CFX Manager software. Chemiluminescence and fluorescence images of Western blots, EMSA, and ThT gels were captured using Bio-Rad Image Lab software on a ChemiDoc imaging system. Fluorescence microscopy imaging was performed using ZEN 2.6 (blue edition) software.
Data analysis	Flow cytometry analyses were performed using FlowJo and multiplex cytokine concentrations were calculated via the ProcartaPlex Analysis App. Image processing, Western blot/EMSA densitometry, and speck quantification were executed using ImageJ/Fiji and CellProfiler (v3.1.9). Next-generation sequencing and bioinformatic analyses were conducted using STAR, Cutadapt (v1.18), Bowtie2 (v2.4.5), SAMtools (v1.14), Picard (v2.26.10), MACS2 (v2.2.7.1), IDR (v2.0.4.2), DiffBind (v3.10), HOMER (v4.11), deepTools (v3.5.2), and Biostrings (v2.68.1) within the R statistical environment (including the tidyverse suite). In silico pathway enrichment and sequence/structure motif predictions were performed using GSEA (v4.4.0) Metascape, G4RNA screener, HOCOMOCO (v11), and TFBIND. Microsoft Excel and GraphPad Prism (v11) were used for statistical anlysis and normalization of data. Image processing and figure design were performed using Adobe Photoshop and Illustrator.

For manuscripts utilizing custom algorithms or software that are central to the research but not yet described in published literature, software must be made available to editors and reviewers. We strongly encourage code deposition in a community repository (e.g. GitHub). See the Nature Portfolio guidelines for submitting code & software for further information.

Data

Policy information about availability of data

All manuscripts must include a data availability statement. This statement should provide the following information, where applicable:

- Accession codes, unique identifiers, or web links for publicly available datasets
- A description of any restrictions on data availability
- For clinical datasets or third party data, please ensure that the statement adheres to our policy

Cell lines and plasmids generated in this study are available from the lead contact upon request. All data needed to evaluate the conclusions in the paper are present in the paper and/or the Supplementary Materials or can be obtained from the corresponding author upon reasonable request. The high-throughput sequencing datasets generated in this study are available in the NCBI Sequence Read Archive (SRA) under the accession codes PRJNA1489410 for the ATAC-seq data and PRJNA1489405 for the 3' RNA-seq data. This paper does not include original code. The data for this study have been deposited in the database.

Research involving human participants, their data, or biological material

Policy information about studies with human participants or human data. See also policy information about sex, gender (identity/presentation), and sexual orientation and race, ethnicity and racism.

Reporting on sex and gender	-
Reporting on race, ethnicity, or other socially relevant groupings	-
Population characteristics	-
Recruitment	-
Ethics oversight	-

Note that full information on the approval of the study protocol must also be provided in the manuscript.

Field-specific reporting

Please select the one below that is the best fit for your research. If you are not sure, read the appropriate sections before making your selection.

☒ Life sciences ☐ Behavioural & social sciences ☐ Ecological, evolutionary & environmental sciences

For a reference copy of the document with all sections, see nature.com/documents/nr-reporting-summary-flat.pdf

Life sciences study design

All studies must disclose on these points even when the disclosure is negative.

Sample size	No statistical methods were used to predetermine sample sizes. For all cell-based and in vitro assays, a minimum of n = 3 independent biological replicates was chosen to ensure reproducibility and to allow for robust statistical evaluation.
Data exclusions	No data were excluded.
Replication	All key findings were robustly reproducible across independent biological replicates, yielding similar outcomes.
Randomization	Randomization was not applicable as no animal models or human subjects were utilized in this study.
Blinding	Blinding was not necessary, as all experimental output relied on quantitative data and not subjective phenotypic observation.

Reporting for specific materials, systems and methods

We require information from authors about some types of materials, experimental systems and methods used in many studies. Here, indicate whether each material, system or method listed is relevant to your study. If you are not sure if a list item applies to your research, read the appropriate section before selecting a response.

Materials & experimental systems

n/a	Involved in the study	
☐	☒	Antibodies
☐	☒	Eukaryotic cell lines
☒	☐	Palaeontology and archaeology
☒	☐	Animals and other organisms
☒	☐	Clinical data
☒	☐	Dual use research of concern
☒	☐	Plants

Methods

n/a	Involved in the study	
☒	☐	ChIP-seq
☐	☒	Flow cytometry
☒	☐	MRI-based neuroimaging

Antibodies

Antibodies used

For protein detection, the following primary antibodies were used: anti-Phospho-p65 (Cell Signaling Technology, Cat# 9936), anti-p65 (Santa Cruz Biotechnology, Cat# sc-8008), anti-Phospho-Histone H2A.X (Cell Signaling Technology, Cat# 9718), anti-GAPDH (Santa Cruz Biotechnology, Cat# sc-69778), and anti-Histone H3 (Abcam, Cat# ab1791), IL-1β (Proteintech, Cat# 26048-1-AP), RelB (Proteintech, Cat# 25027-1-AP), NFKB1 p105/p50 (Proteintech, Cat# 14220-1-AP), NLRP3 (Proteintech, Cat# 30109-1-AP), and the DYKDDDDK (FLAG) epitope (Cell Signaling Technology, Cat# 2368), anti-Phospho-RelB (Ser552) (Cell Signaling Technology, clone D41B9, Cat# 5025T), anti-Phospho-p50 (Santa Cruz Biotechnology, clone A-8, Cat# sc-271908), and anti-Caspase-1 (p20) (Adipogen, clone Casper-1, Cat# AG-20B-0042-C100). The G4-specific single-chain antibody BG4 (clone BG4) was produced in-house. Secondary detection was performed using Donkey anti-rabbit IgG IRDye 800CW (LI-COR Biosciences, Cat# 926-32213), Donkey anti-mouse IgG IRDye 800CW (LI-COR Biosciences, Cat# 926-32212), Goat anti-rabbit IgG Cyanine 3 (Life Technologies, Cat# A10520), Anti-rabbit IgG HRP-linked (Cell Signaling Technology, Cat# 7074P2), and Goat Anti-Mouse IgG HRP-linked (Cell Signaling Technology, Cat# 91196).

Validation

Specific validation tests were not performed by us, but all antibodies have been previously published and validation data is available on the manufacturers website.

Eukaryotic cell lines

Policy information about cell lines and Sex and Gender in Research

Cell line source(s)

Immortalized bone marrow-derived macrophages (iBMDMs) were kindly provided by Prof. Dr. Zeinab Abdullah

Authentication

No authentication was performed.

Mycoplasma contamination

All cell lines used were tested Mycoplamsa negative.

Commonly misidentified lines
(See ICLAC register)

The used cells are not in the list of commonly misidentified cell lines.

Plants

Seed stocks

-

Novel plant genotypes

-

Authentication

-

Flow Cytometry

Plots

Confirm that:

☒ The axis labels state the marker and fluorochrome used (e.g. CD4-FITC).

☒ The axis scales are clearly visible. Include numbers along axes only for bottom left plot of group (a 'group' is an analysis of identical markers).

☒ All plots are contour plots with outliers or pseudocolor plots.

☒ A numerical value for number of cells or percentage (with statistics) is provided.

Methodology

Sample preparation	iBMDMs were prepared as described in DeMagis et. al, 2021.
Instrument	BD FACSCanto II Cell Analyzer
Software	FlowJo
Cell population abundance	Population analysis was not performed, a homogenous immortalized macrophage cell line (iBMDMs) was used.
Gating strategy	Initial gating was performed using Forward and Side Scatter to identify the main singlet macrophage population and exclude subcellular debris or cell aggegates. For activation marker analysis, live cells were selected based on APC-Cy7 staining. The resulting population was then utilized to quantify the Mean Fluorescence Intensity (MFI) of target signals.

☒ Tick this box to confirm that a figure exemplifying the gating strategy is provided in the Supplementary Information.
