# Supplementary material for: G-quadruplex structures affect the immune response to bacterial infection in mouse macrophages
Source: Nat Commun. 2026 Oct 20;17:10955. doi: 10.1038/s41467-026-78610-w (PMC13683368; doi:10.1038/s41467-026-78610-w)
Supplement: Supplementary file 4 [file 41467_2026_78610_MOESM5_ESM.pdf]

nature portfolio

Peer Review File

G-quadruplex structures affect the immune response to bacterial infection in mouse macrophages

Corresponding Author: Dr Katrin Paeschke

This file contains all reviewer reports in order by version, followed by all author rebuttals in order by version.

Version 0:

Reviewer comments:

Reviewer #1

(Remarks to the Author)
This manuscript titled "G-quadruplex structures affect the immune response to bacterial infection in mouse macrophages" explores the regulatory role of G-quadruplex (G4) structures in NF-κB-mediated inflammatory responses of mouse macrophages. The research topic is novel, addressing an important gap in the understanding of transcriptional regulation in innate immunity. However, several critical issues regarding data completeness, mechanism validation, experimental controls, and figure annotation need to be addressed to enhance the rigor and clarity of the work.

Major Concerns
1. Experiments show that LPS pretreatment can prevent PDS-induced G4 stabilization, but the specific mechanism is not elucidated. Why does LPS regulate G4 formation and improve the "LPS-G4-NF-κB" regulatory network ?
2. In Figure 2F, the authors only present the expression and phosphorylation changes of p65. Given that the gene set enrichment analysis (GSEA) identified REL, NF-κB1, and RELB as significantly downregulated pathways (Figure 2A), it is essential to examine the expression and activation status of other NF-κB family members (e.g., RELB, p50) under PDS and LPS treatment conditions. This will help confirm whether G4 stabilization specifically targets p65 or exerts a broader regulatory effect on the NF-κB family, which is critical for supporting the conclusion that G4s interfere with NF-κB-dependent transcriptional networks.

3. The authors report that IL1B and IL18 show relatively low induction by LPS and only minor reductions upon PDS + LPS co-treatment. However, they propose that PDS affects both the "priming phase" (reducing pro-IL1B/pro-IL18 expression) and NLRP3-mediated caspase-1 activation. This raises a critical question: why is the reduction in mature IL1B and IL18 secretion not more pronounced if both steps are impaired? The authors need to provide a clear mechanistic explanation for this discrepancy to verify whether both pathways are indeed compromised and why the cumulative effect on mature cytokine secretion is modest.

4. The authors conclude that PDS-induced G4 formation constrains inflammasome-mediated caspase-1 activation by inhibiting Nlrp3 transcription. However, this conclusion lacks direct evidence of caspase-1 activation status. Additionally, the possibility that G4s directly affect other steps of inflammasome assembly (e.g., NLRP3 oligomerization, ASC speck formation independent of Nlrp3 transcription) has not been ruled out. The authors should supplement experiments to detect cleaved caspase-1 levels (via western blot) and perform in vitro inflammasome reconstitution assays using recombinant NLRP3, ASC, and pro-caspase-1 proteins to confirm whether G4 regulation of inflammasome activation is solely dependent on the transcriptional suppression of Nlrp3.

5. The section "G4s overlapping NF-κB binding sites directly inhibit p65 DNA association" relies on ATAC-seq and public G4seq data, which are indirect evidence. To directly demonstrate G4 dynamics in NF-κB target gene promoters under different treatment conditions (untreated, PDS, LPS, PDS + LPS), the authors should perform genome-wide G4 profiling using established techniques such as BG4-ChIP-seq or G4 CUT&TAG. Comparing G4 enrichment in NF-κB promoter regions across treatment groups will provide definitive evidence for the regulation of G4 formation by PDS and LPS, and strengthen the causal link between G4s and NF-κB binding inhibition.

6. The in vitro binding assays (Figure 5) and their annotations are confusing and lack necessary controls, with key issues including unclear definitions of "competitor" (whether it refers to the complementary strand or PDS) and "Lysate" (whether it

contains NF-κB) in the figure legend, ambiguity about whether NF-κB binds to double-stranded DNA (dsDNA) or G4 structures and whether PDS interferes with this binding, and the absence of a non-G4 dsDNA control to confirm the specificity of NF-κB binding to G4-containing sequences; therefore, the authors should redesign these experiments to directly assess NF-κB binding to NF-κB promoter sequences (containing G4 motifs) using purified NF-κB protein instead of cell lysate, include the complementary strand and PDS as competitors, add a non-G4 dsDNA control (with a consensus NF-κB binding site but no G4-forming sequence) to validate the specificity of G4-mediated inhibition, and revise the figure legend to clearly define all reagents, treatment conditions, and experimental controls. To address the confusion caused by Figure 5A, which aims to verify G4 formation, it is recommended that the authors replace the current in-gel Thioflavin T (ThT) staining with circular dichroism (CD) spectroscopy.

7. The current study is exclusively based on immortalized bone marrow-derived macrophages (iBMDMs) and lacks in vivo validation. To confirm the physiological relevance of the findings, the authors should supplement mouse in vivo infection models to evaluate the effects of G4 stabilizers (PDS) on in vivo inflammatory responses, including cytokine production. This will bridge the gap between in vitro cell line data and potential clinical applications.

Minor comments
8. The legends for several supplementary figures (e.g., Supplementary Figure 3 and 4) are overly brief and fail to clearly indicate the treatment conditions for each group. Additionally, detailed parameters for key experimental methods (e.g., EMSA, ATAC-seq) are not specified, which compromises the reproducibility of the work.

9. For all abbreviations appearing for the first time in the manuscript (e.g., G4, NF-κB, LPS, PDS, EMSA, ATAC-seq, ChIP-qPCR), the full name should be provided followed by the abbreviation in parentheses. This is critical for ensuring readability, especially for readers less familiar with specialized terminology in the field.

10. Several typographical and annotation errors were identified, such as incorrect numbering in the legend of Supplementary Figure 1. The authors should carefully proofread the entire manuscript, including text, figure legends, and supplementary materials, to correct all such errors and ensure consistency in formatting and numbering.

Reviewer #2

(Remarks to the Author)
This is a very interesting manuscript whereby the authors present, for the first time, a mechanism by which G4-formation regulates transcription factors binding recruitment that directly modulates inflammation response. In general the manuscript is well written, experiments are well executed and a substantial amount of evidence in support of the proposed mechanism is provided. Based on this, I am overall supportive for publication in Nat Commun. That said, this reviewers also believes that the addition of a couple of key controls would be ideal to fully support the conclusions and strengthened the manuscript. More specifically:

1) One main limitation of the current study is the direct evidence to support G4-formation at the NF-κB binding sites. Especially considering LPS vs LPS+PDS treatments it would be ideal to have G4 ChIP-qCR data to directly measure the level of G4ness of the promoter under the different conditions tested. This would allow the authors to directly verify their mechanism rather than solely rely on prediction. I appreciate that PDS treatment might affect ChIP, but also an untreated vs LPS treated control might be insightful to check if G4s are actively depleted under LPS treatment to promote NF-κB binding?

2) Another key evidence missing in my opinion is the effect of PDS in altering NF-κB binding by EMSA. The authors provide convincing evidence to show that G4-formation impairs NF-κB binding to the template, but showing active displacement by PDS (so exacerbation of the effect) would be even more convincing and more relevant.

3) I know that the authors refer to a different study showing similar conclusions using PhenDC3 in the discussion of their article. However, showing that the phenotype is not PDS related but other G4-ligands can elicit similar effects would be highly beneficial. This does not have to be necessarily PhenDC3 but any other G4 ligand that is not PDS, just to rule out ligand dependent effect over G4-dependency.

Minor comments:

4) In the introduction I would be more careful in stating that PDS strongly down regulates MYC. The literature in this area is still blurry and the seminal papers from Hurley and co-workers cited by the authors have shown that indirect effects could lead to the observed down regulation. A more holistic and updated view of the role of G4-ligands in mediating transcriptional perturbation is advised here.

Reviewer #3

(Remarks to the Author)
In this manuscript, Schult et al. reported the unique secondary structures called G-quadruplexes to affect NF-κB DNA binding and thereby modulate the macrophage transcriptome during inflammation. By investigating the impact of the G4 ligand pyridostatin (PDS) on macrophages stimulated by bacterial lipopolysaccharide (LPS), they established that G4 stabilization significantly decreased initialization of the inflammatory response, without affecting mitochondrial metabolism, pathogen uptake and killing. Instead, NF-κB-dependent activation markers and cytokines were selectively repressed on the

transcriptional level, leading to a profound attenuation of the inflammatory response. Using a combination approach of global chromatin accessibility measurement, NF-κB ChIP and in silico prediction of potential G4s, they demonstrated that G4 stabilization reduced NF-κB promoter occupancy. In vitro binding assays suggested a model in which G4 folding physically hindered NF-κB dimer binding, while motif-enrichment analysis indicated that G4-containing conformations may favor alternative transcription factors. Based on these data they proposed a novel model in which altered G4 levels significantly affected subsets of the inflammatory response in macrophages. Clearly, the authors verify that G4 formation can be a promising strategy to modulate dysregulated cytokine expression and macrophage activation. This manuscript is well designed and their results are interesting and show great potential for further biomedical applications. Therefore, I recommend this manuscript for publication in Nature Communications after minor revision.

1. On page 3 of the paper, “More than 700,000 sequences in human and mouse genomes are proposed to fold into G4s, and many of these sites are located in functional genomic regions11-14. Their general impact on transcription is well documented for central oncogenes such as c-MYC15,16, which is strongly downregulated upon G4 stabilization by ligands like pyridostatin (PDS)17.” Here, the crucial role of G4s in gene regulation and the methods for regulating G4s are elaborated. This section requires special emphasis and recent advances such as DOI: 10.1038/nchem.1126, 10.1038/s41556-024-01448-1, 10.1002/anie.202503488, 10.1038/s41467-022-29135-5, etc., may consider included in the introduction.

2. On page 8 of the paper, “Figure 1D. representative immunofluorescence image of untreated and PDS.” The authors should consider to provide the images of cell nucleus staining and the images showing their co-localization with BG4. This will enable more accurate description of the situation.

3. Regarding “Figure 1G. FACS analysis of surface marker CD40 indicative of macrophage activation under different conditions.” The authors are suggested to provide representative flow cytometry data in the article or in the supporting information.

4. On page 12 of the paper, the authors should quantify the WB data in Figure 2E in order to measure the phosphorylation status of NF-κB p65. In addition, the captions for Figures 2E and 2F were put in the wrong order and should be corrected.

5. Figure 4 C and D predicted overlaps of G4-forming regions (G4Hunter) and NF-κB binding motifs 317 (HOCOMOCO) in the Il1b_1 (C) and Il1b_2 (D) site. A single prediction is not convincing. Please use other software to predict again the overlapping area between the G4 formation region and the NF-κB binding site, and compare the results to jointly support this conclusion.

Reviewer #4

(Remarks to the Author)
Shult and colleagues demonstrate that stabilization of G-quadruplexes (G4s) in macrophages (bone marrow–derived macrophages and RAW cells) affects innate immune activation triggered by LPS. They show that NF-κB–dependent inflammatory gene induction is selectively repressed by treatment with the G4 ligand pyridostatin (PDS). Furthermore, they find that G4 stabilization reduces NF-κB occupancy at the IL-1β promoter. Based on EMSA assays, the authors propose that G4 folding physically interferes with NF-κB binding to its consensus DNA motif. This study suggests that G4 formation may be beneficial for modulating excessive inflammatory responses. However, several major issues need to be addressed, as outlined below.

1. The basis for the selective influence of G4 stabilization on NF-κB signaling remains unclear. The authors report that three of the five most significantly down-regulated pathways following PDS treatment belong to the NF-κB family (Figure 2A); however, the remaining two pathways are not shown and should be indicated.

More importantly, PDS treatment also markedly suppresses the induction of type I interferon and CXCL10, whose expression is primarily regulated by IRF family transcription factors (IRF3 and IRF7). Therefore, it is necessary to determine whether G4 stabilization also affects IRF recruitment to their cognate cis-regulatory elements. This point should be experimentally addressed.

2. Related to comment #1, it remains unclear whether PDS broadly suppresses LPS-inducible gene expression or preferentially affects a subset of genes according to their dependence on NF-κB for activation. In this regard, it would be important to present examples of LPS-inducible genes that are not affected by PDS treatment, if such genes exist (e.g., in Figure 2C).

3. In Page 27, Lines 414–416, the authors state: “Reporter assays confirmed that G4 formation only inhibits the transcriptional activation, but not the initial phosphorylation and translocation of NF-κB.” However, it is unclear which experimental data support this conclusion. The authors should explicitly indicate the corresponding figures or provide the relevant data.

4. In Figure 5, the source and condition of the lysates used for EMSA are not clearly described. It is unclear whether the lysates were prepared from LPS-stimulated cells or unstimulated cells, and whether NF-κB–DNA binding activity was detected only in lysates from LPS-stimulated cells. The authors should clarify the stimulation conditions and explicitly state the source of the lysates used for the EMSA experiments. Additionally, the authors should address whether the signal that is

resistant to NF-κB competition in Figure 5B depends on LPS stimulation or is present under basal conditions.

Minor point
1. Page 19, Line 306 and 307. "Figure 5C and 5D" should be "Figure 4C and 4D".
2. Page 20, In the scheme of IL-1b locus, there are two 129.196kbs. One should be 129.197kb?

Version 1:

Reviewer comments:

Reviewer #1

(Remarks to the Author)
The revised manuscript has been substantially improved through the addition of multiple new experiments, including validation of additional NF-κB subunits, G4 pulldown assays at the Il1b locus, EMSA experiments under dynamic folding conditions. Most of the raised concerns have been addressed in a substantive and satisfactory manner. However, one major concern remains regarding the genome-wide scope of the proposed mechanism. The central claim of this study that G-quadruplex formation broadly modulates NF-κB-dependent transcription during inflammation relies heavily on bioinformatic predictions overlain with publicly available G4-seq datasets, combined with focused experimental validation at a single locus (Il1b). While the newly added G4 pulldown data provide valuable direct evidence for G4 dynamics at the Il1b promoter, this represents only one genomic site.
Genome-wide G4 profiling under the relevant treatment conditions (untreated, PDS, LPS, PDS+LPS) is critical to establish that the observed mechanism is not an idiosyncrasy of the Il1b locus but instead reflects a global regulatory principle. Without transcriptome-scale G4 mapping data, the causal link between genome-wide NF-κB occupancy changes and G4 stabilization remains correlative and inferred rather than directly demonstrated.The authors are strongly encouraged to perform genome-wide G4 profiling under their experimental conditions to substantiate the global nature of the G4–NF-κB regulatory axis.

Reviewer #2

(Remarks to the Author)
The authors have now addressed my criticisms and I am supportive of publication in Nature Comms

Reviewer #3

(Remarks to the Author)
I recommend this revied manuscript for publication as is.

Reviewer #4

(Remarks to the Author)
The authors have produc vely addressed all concerns and comments raised in my review.I have no further comments.

Open Access This Peer Review File is licensed under a Creative Commons Attribution 4.0 International License, which permits use, sharing, adaptation, distribution and reproduction in any medium or format, as long as you give appropriate credit to the original author(s) and the source, provide a link to the Creative Commons license, and indicate if changes were made.
In cases where reviewers are anonymous, credit should be given to 'Anonymous Referee' and the source.
The images or other third party material in this Peer Review File are included in the article's Creative Commons license, unless indicated otherwise in a credit line to the material. If material is not included in the article's Creative Commons license and your intended use is not permitted by statutory regulation or exceeds the permitted use, you will need to obtain permission directly from the copyright holder.
To view a copy of this license, visit https://creativecommons.org/licenses/by/4.0/

We would like to thank the editor and all reviewers for their time and their thoughtful comments on our manuscript. We highly appreciated them and have carefully addressed each point. Below we have highlighted in green our responses.

Reviewer comments:

Reviewer #1 (Remarks to the Author)

This manuscript titled "G-quadruplex structures affect the immune response to bacterial infection in mouse macrophages" explores the regulatory role of G-quadruplex (G4) structures in NF-κB-mediated inflammatory responses of mouse macrophages. The research topic is novel, addressing an important gap in the understanding of transcriptional regulation in innate immunity. However, several critical issues regarding data completeness, mechanism validation, experimental controls, and figure annotation need to be addressed to enhance the rigor and clarity of the work.

We thank the referee for the comments and pointing out that the research topic is novel and will help to address an important gap in transcription regulation and immune response.

Major Concerns

1. Experiments show that LPS pretreatment can prevent PDS-induced G4 stabilization, but the specific mechanism is not elucidated. Why does LPS regulate G4 formation and improve the "LPS-G4-NF-κB" regulatory network？

We appreciate this insightful question regarding the broader picture of our work. Elucidating the global mechanisms of LPS-mediated G4 regulation is challenging due to the complex transcriptional changes induced by TLR4 activation and constitutes an important avenue for future research. In this study, however, we aimed to offer a hypothesis for the underlying mechanisms controlling the LPS-G4-NF-κB axis, using the example of the *Il1b* promoter.

Because G-quadruplexes require a partially single-stranded DNA state to fold, they are formed at sites of active transcription or replication. Our data suggest that, while LPS treatment increases transcriptional activity (and thereby dynamic ssDNA regions), the concurrent binding of transcription factors (e.g. NF-κB) sterically hinders G4 formation, thereby preventing excessive G4 formation even in the presence of PDS. Indeed, G4 pulldown assays suggest that the abrogation of transcription during PDS+LPS cotreatment may lead to a local collapse of the open chromatin architecture, which impairs both, PDS and TF binding. These new data are now shown in Figure 4. (Please refer to the answer to comment 5 for more detailed information.)

This competitive binding model explains how LPS effectively shifts the equilibrium toward active transcription. We have clarified this hypothesis in the revised discussion and also added more mechanistic evidence by adding PDS during *in vitro* folding, as shown in the updated Figure 5/Supplementary Figure 5.

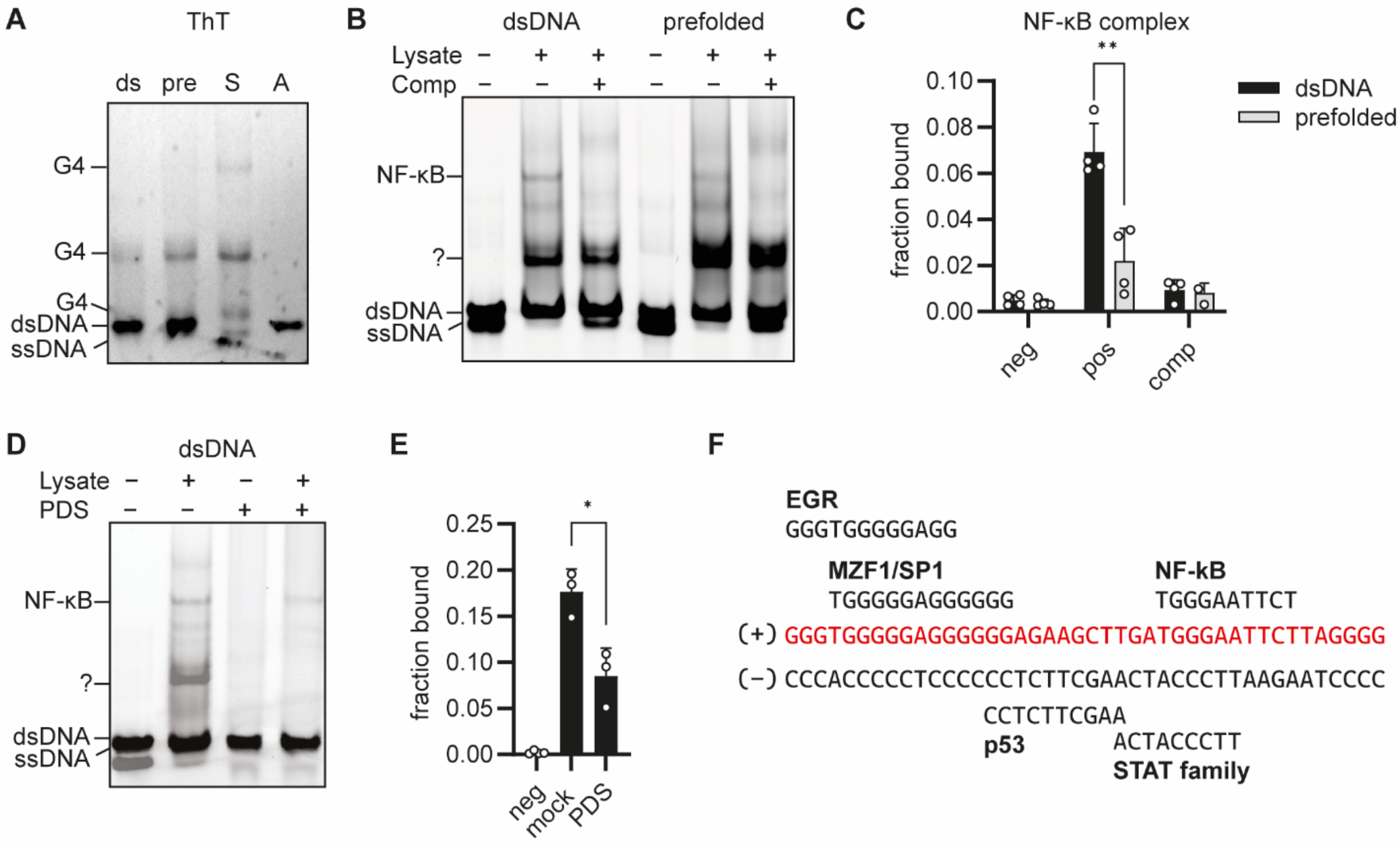

Figure 5: Molecular mechanism of G4-dependent NF-κB inhibition. A. ThT analysis of G4-formation of different oligo compositions. ds: double-stranded, pre: prefolded, S: sense, A: antisense. **B.** Gel shift assay using the G4 sequence including short flanking regions, either folded as a full duplex (dsDNA), or folded separately (prefolded), to induce folding of the G4, and later combination with the negative strand. A visual overview is given in Supplementary Figure 5A. All gel shift experiments were performed in four independent biological replicates. A representative image is shown. **C.** Quantification of NF-κB complex formation in B. Band intensities were normalized to total lane intensity. **D.** Gel shift assay using the IL1B G4 sequence, either annealed in presence or absence of 25 μM PDS to stabilize the folded G4s. All gel shift experiments were performed in three independent biological replicates. A representative image is shown. **E.** Quantification of NF-κB complex formation in D. Band intensities were normalized to total intensity of dsDNA (bands with Cy3 and Cy5 signal). **F.** Predicted TF binding sites in the G4-forming region. Quantification data are represented as mean ± S.D. Statistical significance (Student's t-test) is represented by asterisks: * p<0.05, ** p<0.01, *** p<0.001, **** p<0.0001.

2. In Figure 2F, the authors only present the expression and phosphorylation changes of p65. Given that the gene set enrichment analysis (GSEA) identified REL, NF-κB1, and RELB as significantly downregulated pathways (Figure 2A), it is essential to examine the expression and activation status of other NF-κB family members (e.g., RELB, p50) under PDS and LPS treatment conditions. This will help confirm whether G4 stabilization specifically targets p65 or exerts a broader regulatory effect on the NF-κB family, which is critical for supporting the conclusion that G4s interfere with NF-κB-dependent transcriptional networks.

We agree that the expression and activation status of the other NF-kB subunits can provide valuable insights into the cellular response after LPS/PDS treatment. Thus, we used RELB and NF-kB1 (p105/p50) and quantified total protein and phosphorylation after priming with LPS, PDS cotreatment and after full inflammasome activation with nigericin. There were no apparent differences for p50 and RELB phosphorylation and expression. Thus, we suggest that p65 is the major subunit responsible for the observed effects, although it is likely that it acts as part of different heterodimeric complexes with the other subunits, which may explain the GSEA data. We have here included the Western blots of this analysis, which are now part of Supplementary Figure 2.

Further we state in the results “Western blot analysis of phospho-p65 (Ser536) and phospho-RELB (Ser552) revealed that phosphorylation still occurred under PDS + LPS co-treatment, albeit at reduced levels, suggesting that primary upstream signaling is initiated but quantitatively dampened (Figure 2E). A similar phenotype was observed for RELB, which was also detected in the PDS-dependent GSEA signature (Supplementary Figure 2A, B). In contrast, neither LPS nor PDS had significant impact on phosphorylation status (Ser337) or expression of NFKB1 p50 (Supplementary Figure 2A, B).”

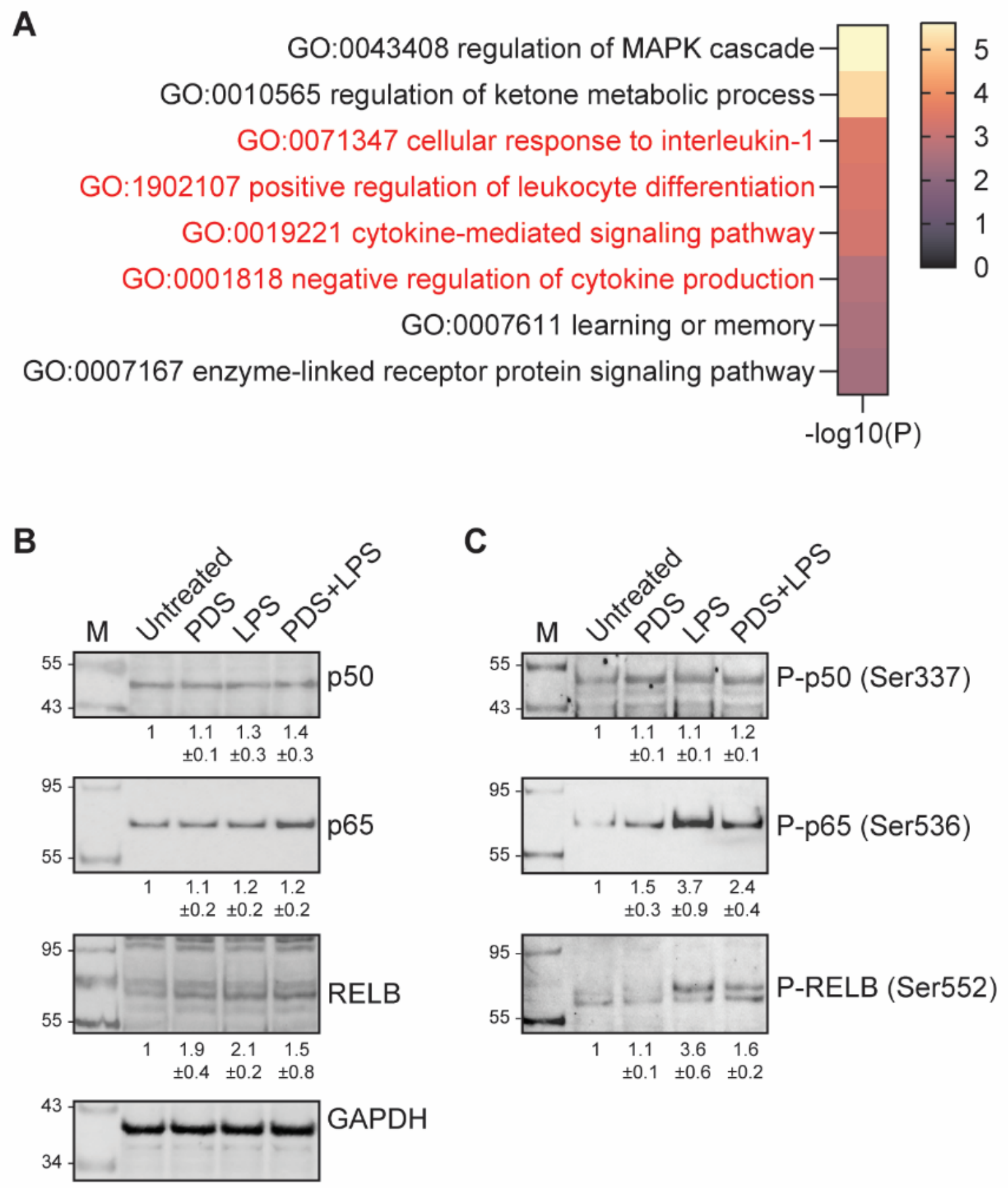

Supplementary Figure 2: Western blot analysis of additional NF-κB subunits. **A.** Pathway enrichment analysis of genes not negatively affected by PDS. Refer to Supplementary Table 1 for a full list of genes. Data are sorted by negative P-value (-log10(P)). Pathways relevant for immune regulation are marked in red. **B.** Western blot analysis of total expression levels of NF-κB subunits after PDS, LPS and co-treatment. **C.** Phosphorylation status of the subunits shown in B. All bands were normalized to GAPDH. Relative quantification of the bands compared to untreated are shown below the respective western blots. Data are represented as mean ± S.E.M. All experiments were performed in three independent biological replicates.

3. The authors report that IL1B and IL18 show relatively low induction by LPS and only minor reductions upon PDS + LPS co-treatment. However, they propose that PDS affects both the "priming phase" (reducing pro-IL1B/pro-IL18 expression) and NLRP3-mediated caspase-1 activation. This raises a critical question: why is the reduction in mature IL1B and IL18 secretion not more pronounced if both steps are impaired? The authors need to provide a clear mechanistic explanation for this discrepancy to verify whether both pathways are indeed compromised and why the cumulative effect on mature cytokine secretion is modest.

We appreciate the reviewer’s critical assessment. We realize that the side-by-side presentation of our 'priming-only' and 'full activation' assays may have caused some confusion regarding the magnitude of the PDS effect.

The minor reductions the reviewer likely refers to were observed in the LPS-only condition (Figure 3D/E). Because LPS alone does not efficiently trigger inflammasome assembly, the baseline secretion of mature IL1B and IL18 in this condition is inherently low, occluding any visible PDS effects. We have included western blot analysis showing negligible IL1B secretion albeit high intracellular levels of pro-IL1B, which are efficiently suppressed by PDS. The observed small increase after LPS/LPS+PDS is therefore most likely due to cellular leakage of pro-IL1B/18, rather than active secretion.

In line with this, we also see that IL1B translation is not fully abrogated and present in the cytosol even after PDS co-treatment, validating also the SEAP secretion data in Figure 2F.

Similar results were obtained for NLRP3, which is induced by LPS, and suppressed by PDS cotreatment, albeit the PDS effect is less pronounced. These results are now included in Supplemenary Figure 3 F. In the results section these experiments are summarized as following: “This observation corroborated the reduced transcription show in the RNA-Seq data and translation (Supplementary Figure 3F) of pro-inflammatory factors and indicates that the deficient priming is responsible for impaired inflammasome assembly. In contrast, addition of PDS only during nigericin treatment did not affect the functionality of the inflammasome, as Caspase-1 and IL1B maturation and secretion remained unaffected (Supplementary Figure 3F).”

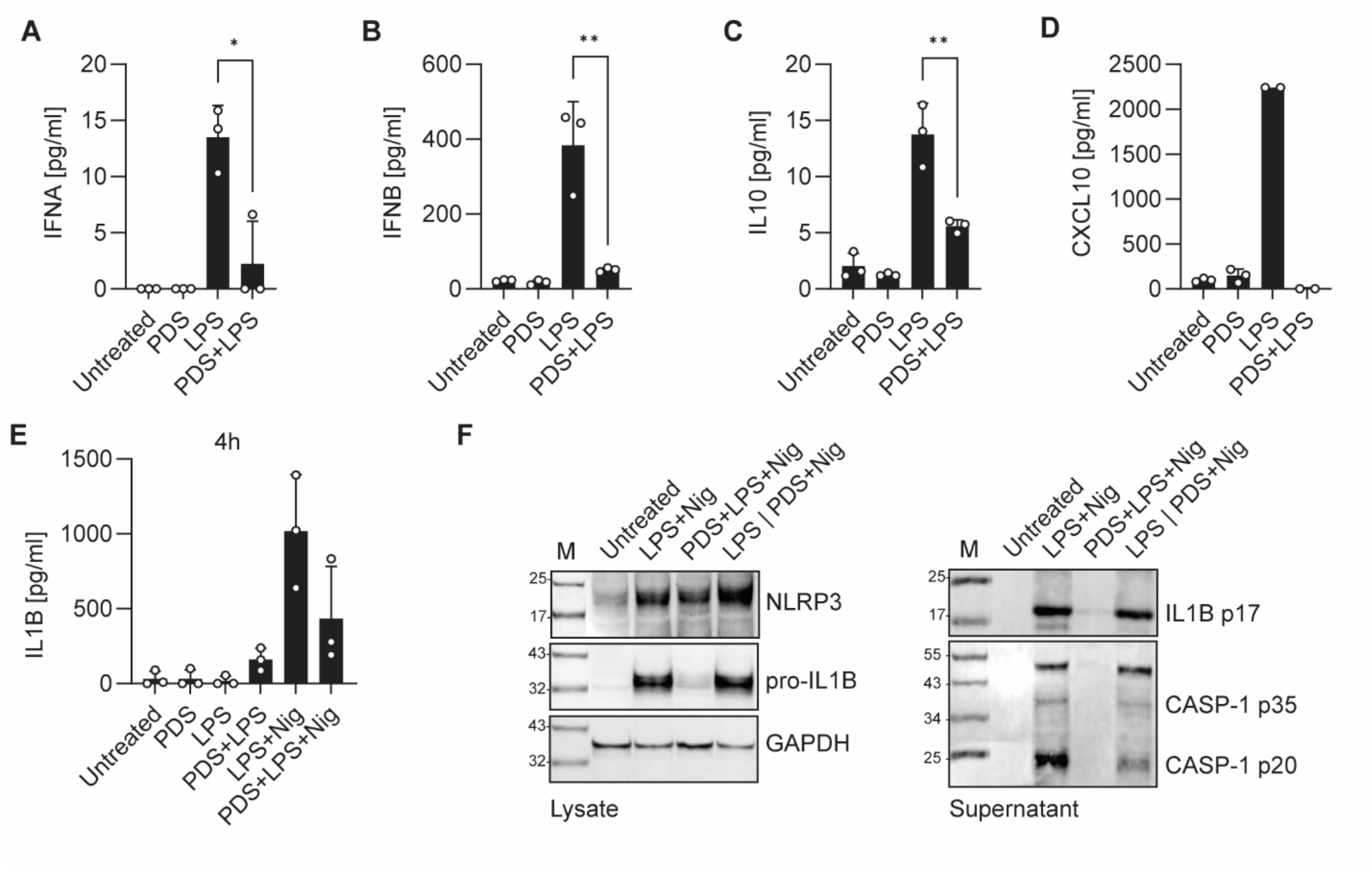

Supplementary Figure 3: Downstream effects of the PDS-dependent transcriptional block of NF-κB. A-D. Secretion of additional important and LPS-dependent cytokines affected by PDS treatment. **E.** As in Figure 3H, but 4h post stimulation. **F.** Western blot analysis of central components of inflammasome function under different treatments.

However, when we apply the standard two-step activation protocol (LPS + Nigericin, Figure 3H), mature cytokine secretion is massively upregulated, and the inhibitory effect of PDS becomes clearly visible (>10-fold). This significant reduction under fully activating conditions thus corroborates our proposed mechanism that PDS impairs priming and activation phase, by blocking the initial transcriptional activation.

4. The authors conclude that PDS-induced G4 formation constrains inflammasome-mediated caspase-1 activation by inhibiting Nlrp3 transcription. However, this conclusion lacks direct evidence of caspase-1 activation status. Additionally, the possibility that G4s directly affect other steps of inflammasome assembly (e.g., NLRP3 oligomerization, ASC speck formation independent of Nlrp3 transcription) has not been ruled out. The authors should supplement experiments to detect cleaved caspase-1 levels (via western blot) and perform in vitro inflammasome reconstitution assays using recombinant NLRP3, ASC, and pro-caspase-1 proteins to confirm whether G4 regulation of inflammasome activation is solely dependent on the transcriptional suppression of Nlrp3.

We thank the reviewer for these constructive suggestions to functionally support our activation hypothesis.

To address the functionality of the inflammasome, we have added a caspase-1/IL1B western blot from the supernatant of treated cells and found that caspase-1 activation and subsequent IL1B maturation is negligible in all conditions without the second signal. Moreover, in these experiments no PDS was added during nigericin activation, showing that transcriptional suppression during priming is indeed sufficient for inflammasome inhibition.

We also tested whether PDS directly affects inflammasome assembly by adding PDS only during nigericin treatment and observing the downstream effects. We found that PDS in the used concentration does not impair Caspase-1 activation and IL1B secretion.

We have added these data to the results in Supplementary Figure 3, which now include the statement: "This observation corroborated the reduced transcription show in the RNA-Seq data and translation (Supplementary Figure 3F) of pro-inflammatory factors and indicates that the deficient priming is responsible for impaired inflammasome assembly. In contrast, addition of PDS only during nigericin treatment did not affect the functionality of the inflammasome, as Caspase-1 and IL1B maturation and secretion remained unaffected (Supplementary Figure 3F).

To confirm the functional consequences of this defect, we measured secretion of mature IL1B at 4 h and 8 h post-stimulation (Figure 3H, Supplementary Figure 3E). As expected, 8 h LPS + nigericin treatment induced a >1000-fold increase in IL1B secretion relative to LPS alone (Figure 3H). In contrast, co-treatment with PDS substantially reduced IL1B release at both time points, with a particularly strong effect after 8 h (Figure 3H, Supplementary Figure 3E)."

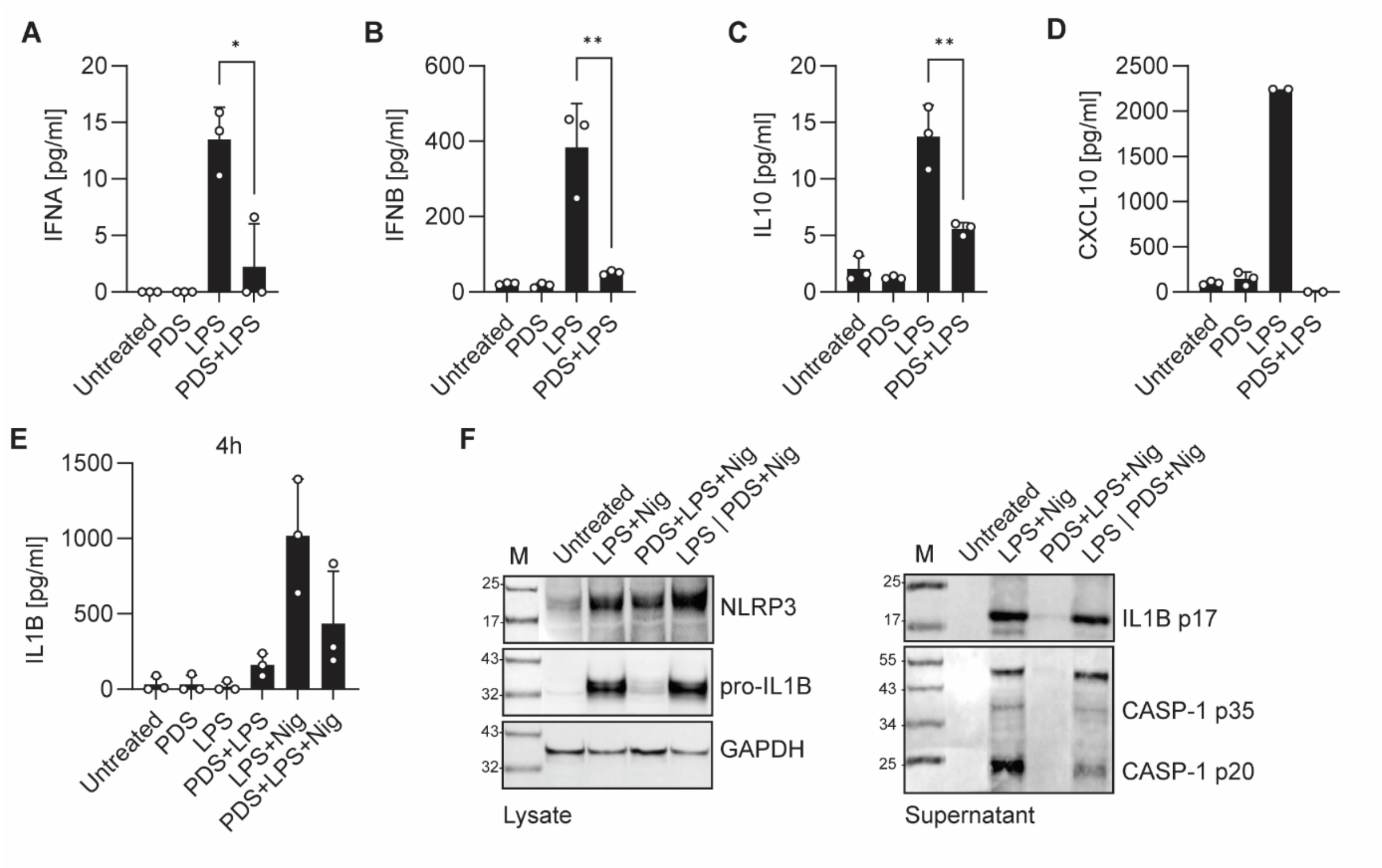

5. The section "G4s overlapping NF-κB binding sites directly inhibit p65 DNA association" relies on ATAC-seq and public G4seq data, which are indirect evidence. To directly demonstrate G4 dynamics in NF-κB target gene promoters under different treatment conditions (untreated, PDS, LPS, PDS + LPS), the authors should perform genome-wide G4 profiling using established techniques such as BG4-ChIP-seq or G4 CUT&TAG. Comparing G4 enrichment in NF-κB promoter regions across treatment groups will provide definitive evidence for the regulation of G4 formation by PDS and LPS, and strengthen the causal link between G4s and NF-κB binding inhibition.

We agree that direct validation of G4 formation at the relevant sites is an important datapoint. However, BG4-based methods often struggle with chemically stabilized G4s as they compete for binding surfaces (Masson et al., 2021) in agreement with our own data (Reviewer Figure R1, unpublished) and BG4 recognition can differ from the subset stabilized by PDS (Ray et al., 2020). Thus, we attempted a modified BioCyTASQ pulldown protocol (Feng et al., 2022), since TASQ-based ligands have already been successfully tested on rG4s stabilized with PDS (Turcotte et al., 2023). Indeed, we could show that PDS treatment led to a robust PDS-dependent signal increase at the putative G4 in IL1B promoter, as shown in the updated Figure 4.

We state now in the result section: "To explore the dynamic relationship between G4 formation and transcription factor occupancy at the *Il1b* locus, we performed a chemical G4 pull-down assay using a modified version of the G4-DNA precipitation (G4DP) protocol established by Feng et al.[48], as classical BG4-ChIP approaches can be limited by steric competition between the antibody and small-molecule ligands. The specificity of the assay was confirmed by a robust, PDS-dependent enrichment of the G4-containing region within the *Il1b* promoter, whereas no increase was observed at non-G4 control regions within the *Il1b* CDS or the *Ifnb* promoter. Interestingly, LPS stimulation alone also significantly increased the G4 pull-down efficiency. This is consistent with the assumption that under basal conditions, the *Il1b* promoter remains transcriptionally dormant, thus rarely adopting a single-stranded conformation. Upon LPS activation, the massive induction of transcription

dynamically generates transient single-stranded DNA intermediates within the transcription bubble, substantially increasing the kinetic frequency of G4 folding. Finally, co-treatment with PDS and LPS reduced the pull-down efficiency back to basal levels. This finding aligns well with our immunofluorescence and FACS-based observations (Figure 1E, Supplementary Figure 1A) and points toward a transcriptional feedback mechanism: due to the early blockade of NF-kB by PDS, the recruitment of RNA polymerases is inhibited leading to the collapse of the transcription bubble and reversion to the dormant state."

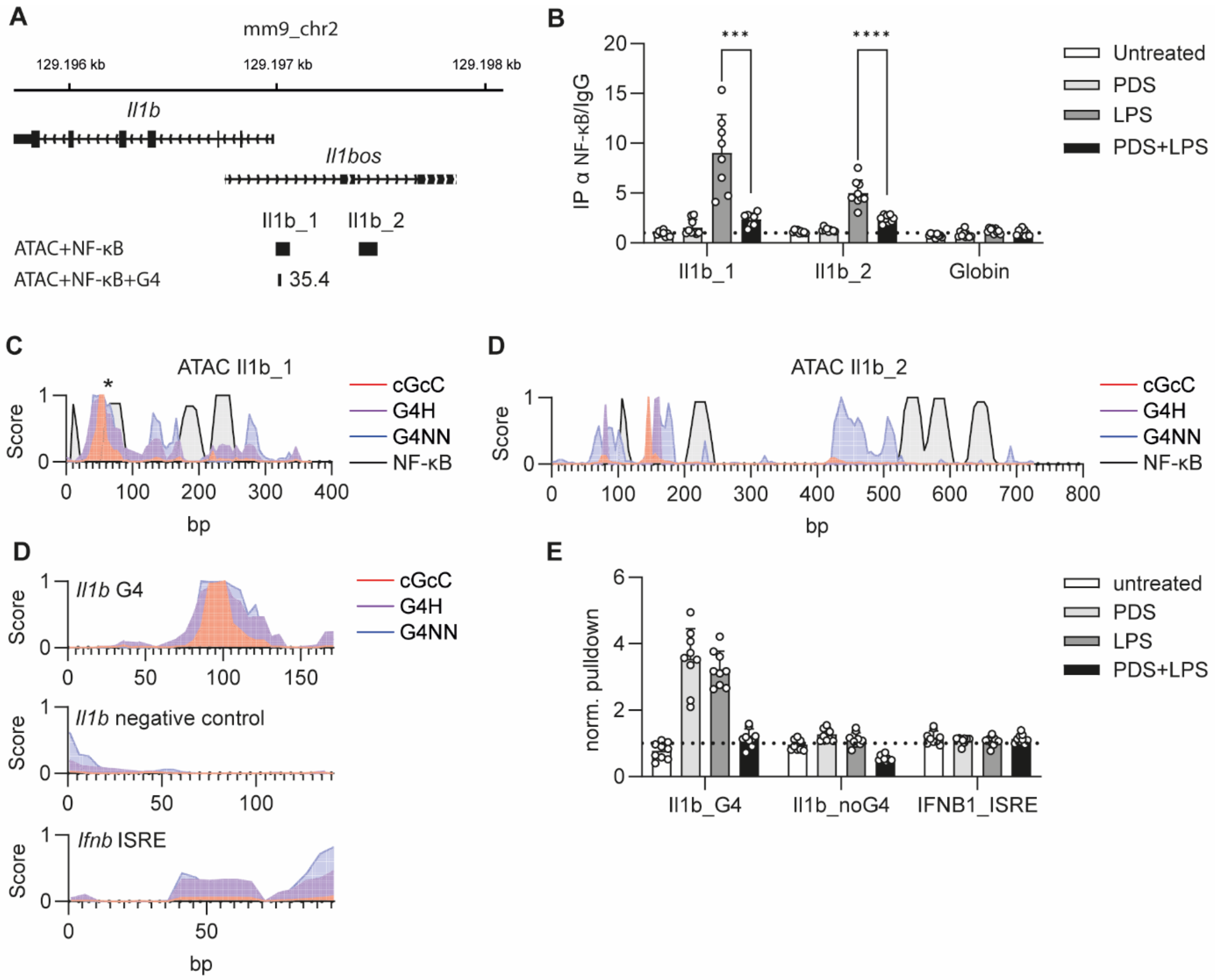

Figure 4: G4-dependence of NF-κB binding to DNA. A. Genomic locus of *Il1b*. ATAC+NF-κB denotes regions of accessible chromatin, overlapping known NF-κB binding sites after LPS treatment (Peterson et al., 2018). ATAC+NF-κB+G4 indicates G4s detected via previous G4seq analyses (Marsico et al., 2019). **B.** ChIP-qPCR assessment of NF-KB association to the selected genomic loci under different treatment conditions. **C, D.** Predicted overlaps of G4-forming regions (G4RNA Screener) and NF-κB binding motifs (HOCOMOCO/TFBIND) in the Il1b_1 (**C**) and Il1b_2 (**D**) site. Data are normalized to an IgG control ChIP performed in parallel. The coding region of the globin gene was used as background control. Data are represented as mean ± S.D. ChIP-qPCR experiments were performed in biological and technical triplicate. All replicate means are shown as symbols. **E.** Prediction of G4 forming regions within the selected targets for G4DP qPCR. For direct comparison, all scores are normalized to the maximal value of the *Il1b* G4 sequence. **F.** G4DP qPCR results for the selected targets, showing the normalized pulldown efficiencies relative to input and the globin CDS. Statistical significance (Student's t-test) is represented by asterisks: * $p<0.05$, ** $p<0.01$, *** $p<0.001$, **** $p<0.0001$.

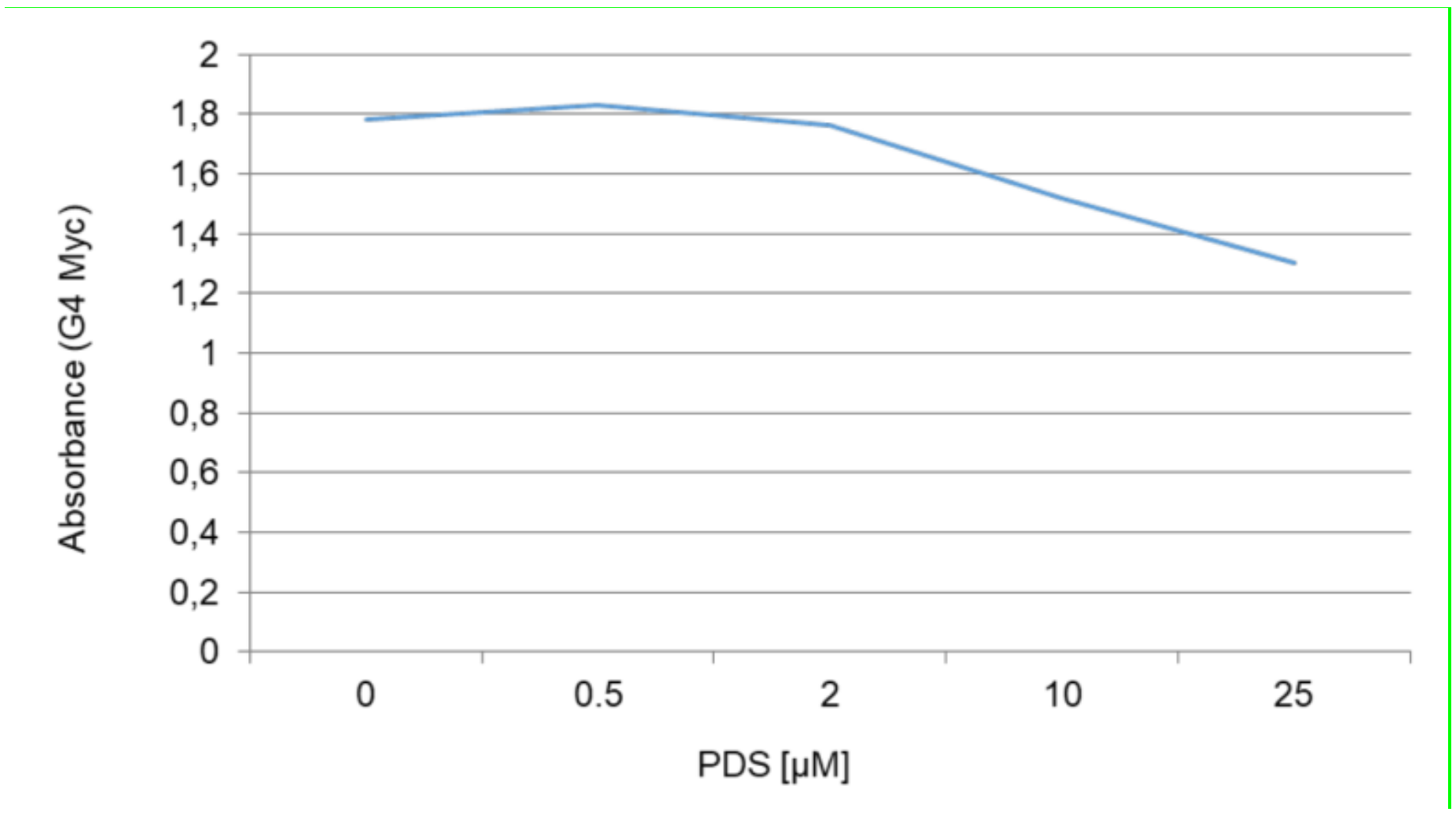

Figure R1: ELISA assay assessing the binding capability of BG4 to the MYC-G4 in response to different concentrations of PDS.

6. The in vitro binding assays (Figure 5) and their annotations are confusing and lack necessary controls, with key issues including unclear definitions of "competitor" (whether it refers to the complementary strand or PDS) and "Lysate" (whether it contains NF-κB) in the figure legend, ambiguity about whether NF-κB binds to double-stranded DNA (dsDNA) or G4 structures and whether PDS interferes with this binding, and the absence of a non-G4 dsDNA control to confirm the specificity of NF-κB binding to G4-containing sequences; therefore, the authors should redesign these experiments to directly assess NF-κB binding to NF-κB promoter sequences (containing G4 motifs) using purified NF-κB protein instead of cell lysate, include the complementary strand and PDS as competitors, add a non-G4 dsDNA control (with a consensus NF-κB binding site but no G4-forming sequence) to validate the specificity of G4-mediated inhibition, and revise the figure legend to clearly define all reagents, treatment conditions, and experimental controls. To address the confusion caused by Figure 5A, which aims to verify G4 formation, it is recommended that the authors replace the current in-gel Thioflavin T (ThT) staining with circular dichroism (CD) spectroscopy.

We have realized that the corresponding method paragraph was missing from the materials and methods part of the submitted version of the manuscript. We apologize for this oversight. We have added the part and revised the corresponding figure legends and text to make this part clearer. We can see the point regarding the ThT staining, however we think it is the most appropriate here, to verify that the G4s are present under the specific experimental conditions used here.

We have also added a visual overview of the folding procedure in Supplementary Figure 5A, to make the method more easily understandable.

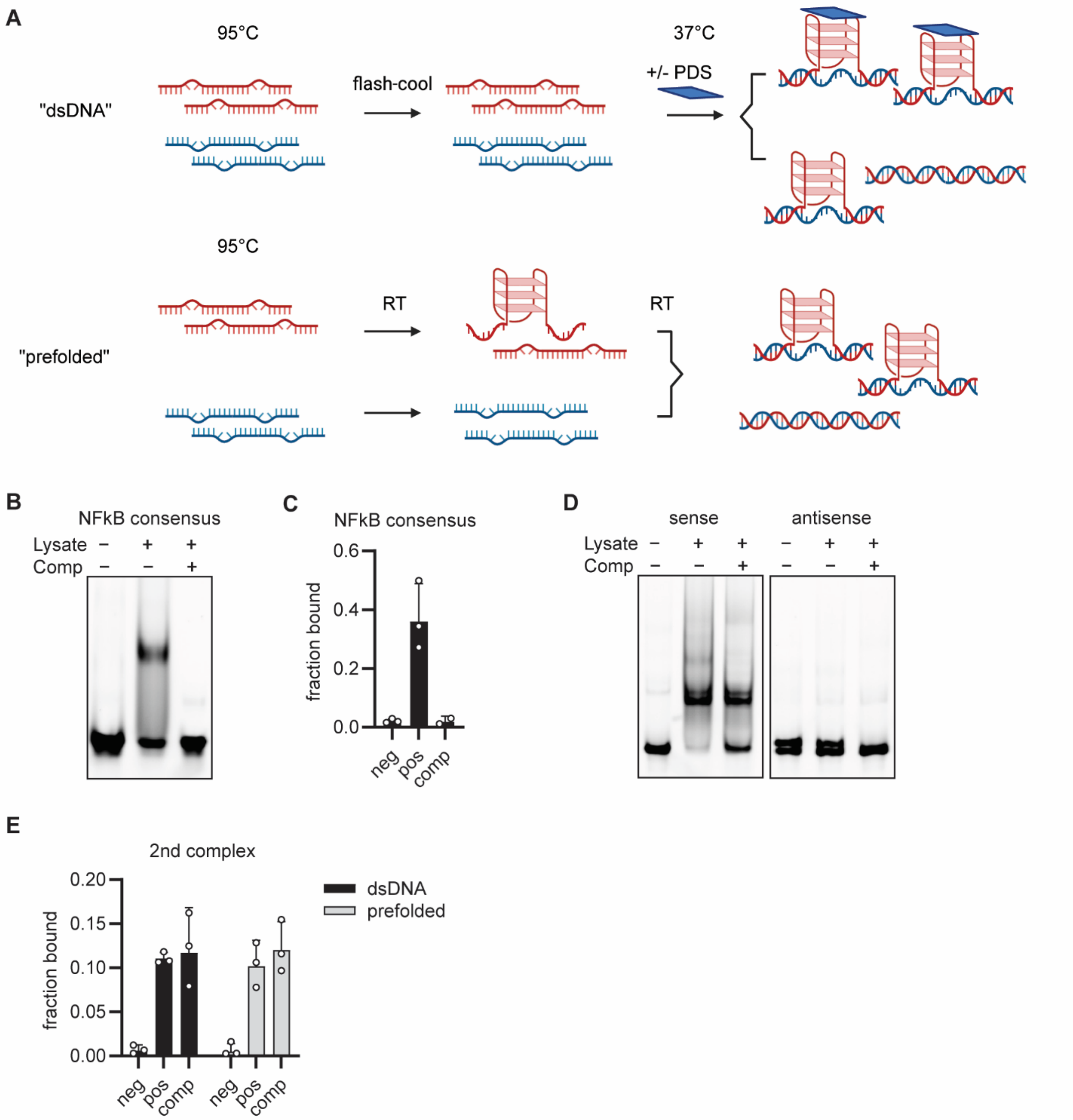

Supplementary Figure 5: Molecular mechanism of G4-dependent NF-κB inhibition. A. Schematic of the folding procedure for the generation of the "dsDNA" and "prefolded" bait for EMSA assays. **B.** Technical validation of shift assays using a double-stranded consensus NF-κB oligo as template and competitor. **C.** Quantification of NF-κB complex formation in B. Band intensities were normalized to total lane intensity. **D.** Control EMSA assay with the single strand oligos. **E.** Quantification of the second, non-NF-κB containing complex shown in Figure 5B.

7. The current study is exclusively based on immortalized bone marrow-derived macrophages (iBMDMs) and lacks in vivo validation. To confirm the physiological relevance of the findings, the authors should supplement mouse in vivo infection models to evaluate the

effects of G4 stabilizers (PDS) on in vivo inflammatory responses, including cytokine production. This will bridge the gap between in vitro cell line data and potential clinical applications.

We would be excited to confirm our results *in vivo*, but the core focus of our work is the intracellular mechanism by which G4s may affect the LPS-induced transcriptional network at the DNA level. The *in vivo* effects of chemical G4-stabilization will, however, be addressed in the corresponding back-to-back publication of Maione et al. using PhenDC3, which had comparable effects in our *in vitro* model.

Minor comments

8. The legends for several supplementary figures (e.g., Supplementary Figure 3 and 4) are overly brief and fail to clearly indicate the treatment conditions for each group. Additionally, detailed parameters for key experimental methods (e.g., EMSA, ATAC-seq) are not specified, which compromises the reproducibility of the work.

We have revised this part, for details please refer to the answer to comment 6.

9. For all abbreviations appearing for the first time in the manuscript (e.g., G4, NF-κB, LPS, PDS, EMSA, ATAC-seq, ChIP-qPCR), the full name should be provided followed by the abbreviation in parentheses. This is critical for ensuring readability, especially for readers less familiar with specialized terminology in the field.

We have revised the manuscript and added introductions to the missing abbreviations.

10. Several typographical and annotation errors were identified, such as incorrect numbering in the legend of Supplementary Figure 1. The authors should carefully proofread the entire manuscript, including text, figure legends, and supplementary materials, to correct all such errors and ensure consistency in formatting and numbering.

We combed the text thoroughly for any remaining errors.

Reviewer #2 (Remarks to the Author)

This is a very interesting manuscript whereby the authors present, for the first time, a mechanism by which G4-formation regulates transcription factors binding recruitment that directly modulates inflammation response. In general the manuscript is well written, experiments are well executed and a substantial amount of evidence in support of the proposed mechanism is provided. Based on this, I am overall supportive for publication in Nat Commun. That said, this reviewers also believes that the addition of a couple of key controls would be ideal to fully support the conclusions and strengthened the manuscript. More specifically:

We thank the referee for supporting this work and provided constructive comments and experiments.

1) One main limitation of the current study is the direct evidence to support G4-formation at the NF-κB binding sites. Especially considering LPS vs LPS+PDS treatments it would be ideal to have G4 ChIP-qCR data to directly measure the level of G4ness of the promoter under the different conditions tested. This would allow the authors to directly verify their mechanism rather than solely rely on prediction. I appreciate that PDS treatment might affect ChIP, but also an untreated vs LPS treated control might be insightful to check if G4s are actively depleted under LPS treatment to promote NF-κB binding?

Please refer to reviewer 1, question 5 for a detailed answer, and see novel pull down data that we included to address this very valuable point. We are very excited that these pull down experiments were working and are fully in agreement with our model and previous data. The data are shown in the updated Figure 4.

2) Another key evidence missing in my opinion is the effect of PDS in altering NF-κB binding by EMSA. The authors provide convincing evidence to show that G4-formation impairs NF-κB binding to the template, but showing active displacement by PDS (so exacerbation of the effect) would be even more convincing and more relevant.

We strongly agree with the reviewer's idea, however, direct displacement (at least under the conditions used in our EMSA) was not efficient. We therefore hypothesized that the major issue is that the DNA in *in vitro* experiments is much less dynamic than the actively transcribed genomic region. To address this comment, we tried to mimic the replication bubbles by melting/flash cooling and refolding +/- PDS. Here we could indeed observe an effect, as PDS stabilized G4s and thus inhibited NF-κB binding.

We included these novel data in Supplementary Figure 5. The experiments are described in the result section: "Importantly, when denatured target oligos were annealed in presence of PDS, favoring G4-containing conformations over dsDNA, the NF-κB-dependent shift was markedly reduced (Figure 5D, E)."

And pertaining to the second complex: "In line with previous observations that G4 ligands can actively compete with G4-binding proteins, we found that this complex is also highly sensitive to PDS treatment (Supplementary Figure 5D)."

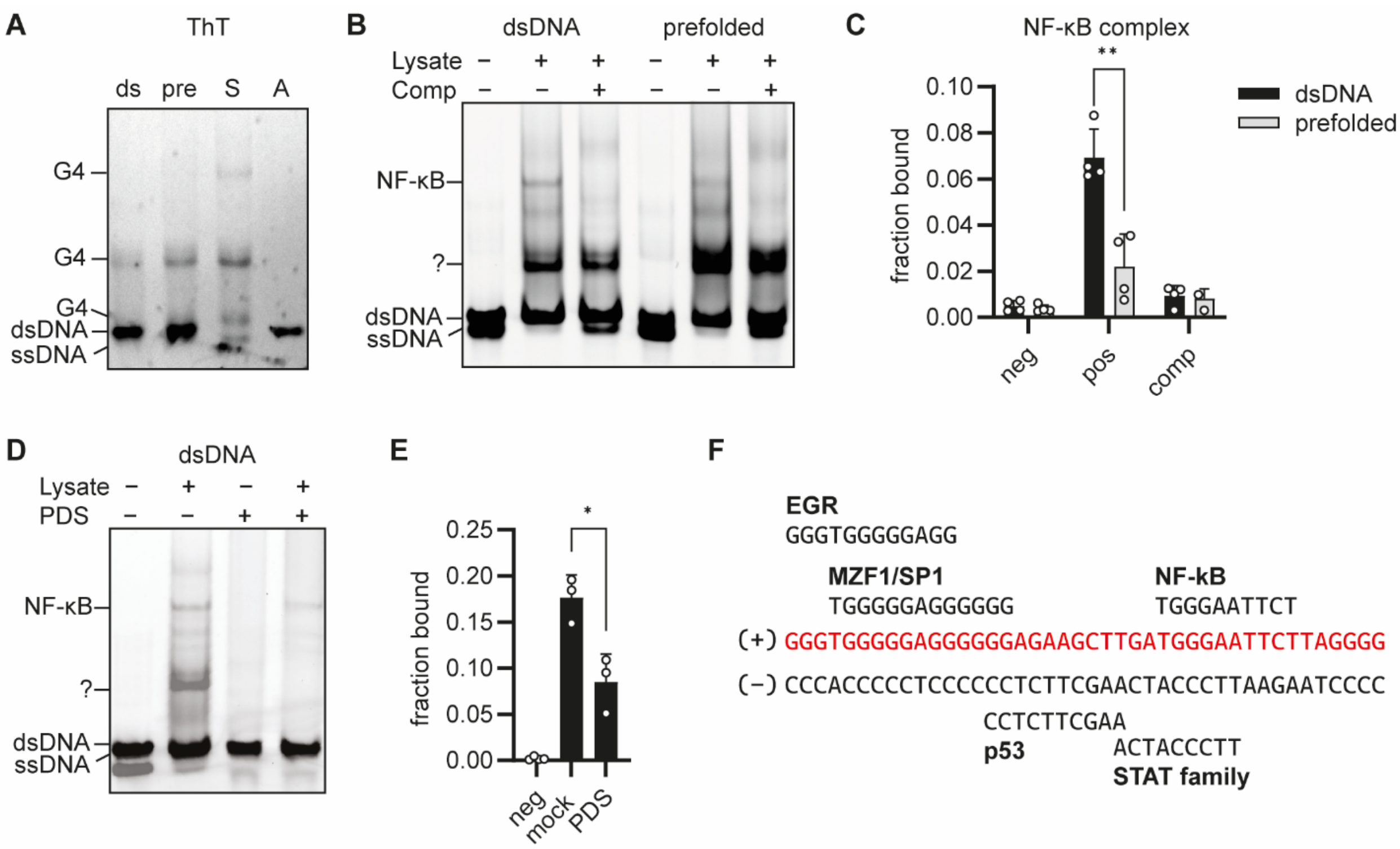

Figure 5: Molecular mechanism of G4-dependent NF-κB inhibition. A. ThT analysis of G4-formation of different oligo compositions. ds: double-stranded, pre: prefolded, S: sense, A: antisense. **B.** Gel shift assay using the G4 sequence including short flanking regions, either folded as a full duplex (dsDNA), or folded separately (prefolded), to induce folding of the G4, and later combination with the negative strand. A visual overview is given in Supplementary Figure 5A. All gel shift experiments were performed in four independent biological replicates. A representative image is shown. **C.** Quantification of NF-κB complex formation in B. Band intensities were normalized to total lane intensity. **D.** Gel shift assay using the IL1B G4 sequence, either annealed in presence or absence of 25 µM PDS to stabilize the folded G4s. All gel shift experiments were performed in three independent biological replicates. A representative image is shown. **E.** Quantification of NF-κB complex formation in D. Band intensities were normalized to total intensity of dsDNA (bands with Cy3 and Cy5 signal). **F.** Predicted TF binding sites in the G4-forming region. Quantification data are represented as mean ± S.D. Statistical significance (Student's t-test) is represented by asterisks: * $p<0.05$, ** $p<0.01$, *** $p<0.001$, **** $p<0.0001$.

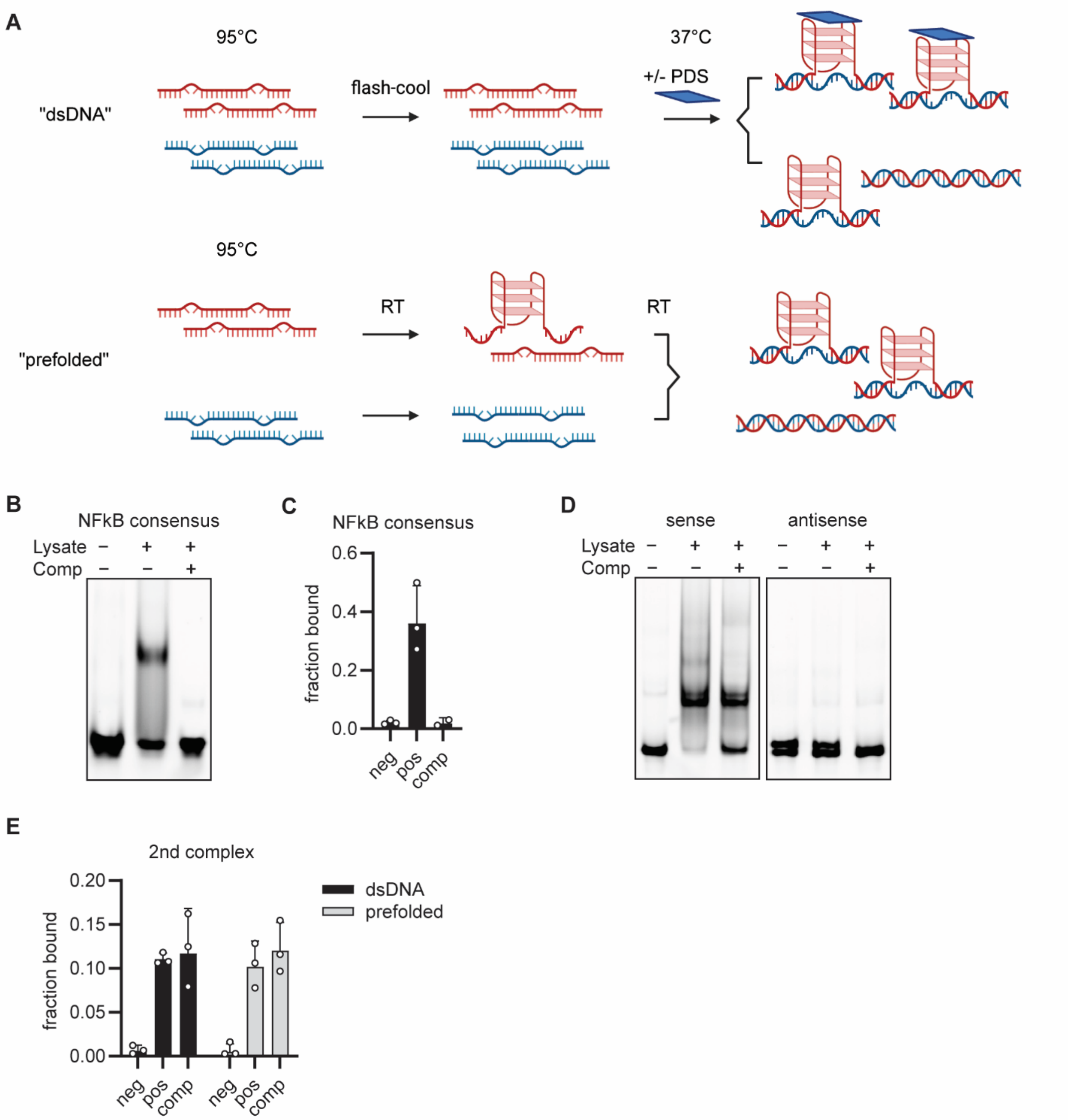

Supplementary Figure 5: Molecular mechanism of G4-dependent NF-κB inhibition. A. Schematic of the folding procedure for the generation of the “dsDNA” and “prefolded” bait for EMSA assays. **B.** Technical validation of shift assays using a double-stranded consensus NF-κB oligo as template and competitor. **C.** Quantification of NF-κB complex formation in B. Band intensities were normalized to total lane intensity. **D.** Control EMSA assay with the single strand oligos. **E.** Quantification of the second, non-NF-κB containing complex shown in Figure 5B.

3) I know that the authors refer to a different study showing similar conclusions using PhenDC3 in the discussion of their article. However, showing that the phenotype is not PDS related but other G4-ligands can elicit similar effects would be highly beneficial. This does not

have to be necessarily PhenDC3 but any other G4 ligand that is not PDS, just to rule out ligand dependent effect over G4-dependency.

Of course, the referee is correct. However, it is also known that different G4 ligands act at different G4 targets. Regardless we treated cells with the well-known G4 ligand, CX 5461. We have repeated the ChIP with NFKB p65 after CX5461+LPS treatment and determined a very similar reduction in binding after G4 stabilization by CX4561 and the data are shown here as Reviewer Figure R2.

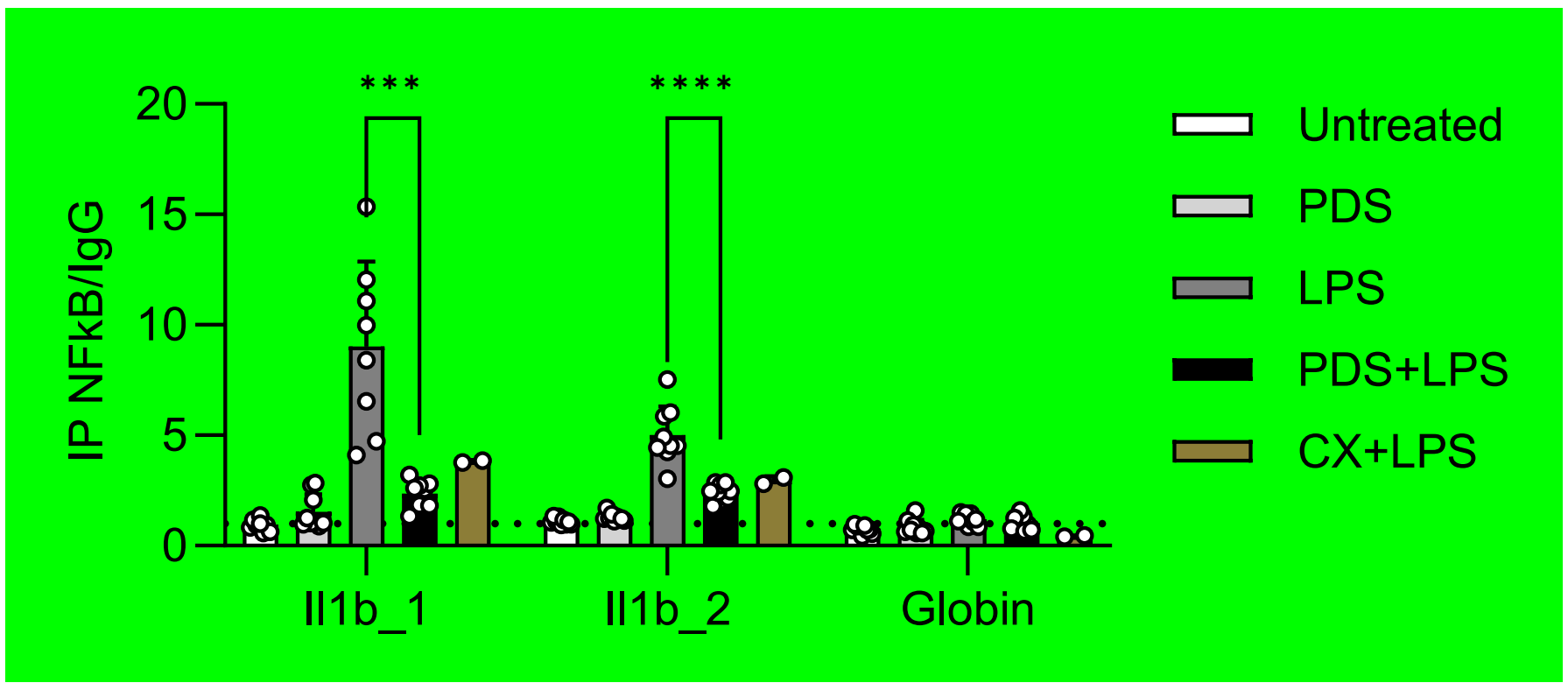

Figure R2: ChIP-qPCR of different NF-kB p65 targets and globin as a negative control. CX5461 was used in a concentration of 1µM for 4h in addition to LPS treatment.

Minor comments:

4) In the introduction I would be more careful in stating that PDS strongly down regulates MYC. The literature in this area is still blurry and the seminal papers from Hurley and co-workers cited by the authors have shown that indirect effects could lead to the observed down regulation. A more holistic and updated view of the role of G4-ligands in mediating transcriptional perturbation is advised here.

We fully agree that our original phrasing was too definitive and oversimplified the complex mechanistic effects of PDS treatment. Following the reviewer's excellent suggestion, we have revised the introduction to reflect a more differentiated view. We now explicitly acknowledge that G4-ligand-mediated transcriptional perturbations (such as the downregulation of *c-MYC*) are highly complex and likely driven by a combination of direct stabilization and secondary, indirect cellular effects, as demonstrated by the seminal work in the field

Reviewer #3 (Remarks to the Author)

In this manuscript, Schult et al. reported the unique secondary structures called G-quadruplexes to affect NF-κB DNA binding and thereby modulate the macrophage transcriptome during inflammation. By investigating the impact of the G4 ligand pyridostatin (PDS) on macrophages stimulated by bacterial lipopolysaccharide (LPS), they established that G4 stabilization significantly decreased initialization of the inflammatory response, without affecting mitochondrial metabolism, pathogen uptake and killing. Instead, NF-κB-dependent activation markers and cytokines were selectively repressed on the transcriptional level, leading to a profound attenuation of the inflammatory response. Using a combination approach of global chromatin accessibility measurement, NF-κB ChIP and in silico prediction of potential G4s, they demonstrated that G4 stabilization reduced NF-κB promoter occupancy. In vitro binding assays suggested a model in which G4 folding physically hindered NF-κB dimer binding, while motif-enrichment analysis indicated that G4-containing conformations may favor alternative transcription factors. Based on these data they proposed a novel model in which altered G4 levels significantly affected subsets of the inflammatory response in macrophages. Clearly, the authors verify that G4 formation can be a promising strategy to modulate dysregulated cytokine expression and macrophage activation. This manuscript is well designed and their results are interesting and show great potential for further biomedical applications. Therefore, I recommend this manuscript for publication in Nature Communications after minor revision.

We thank this reviewer for the helpful and very positive comments and for pointed out how well designed our work and results are. We appreciate the constructive comments and suggestions.

1. On page 3 of the paper, “More than 700,000 sequences in human and mouse genomes are proposed to fold into G4s, and many of these sites are located in functional genomic regions11-14. Their general impact on transcription is well documented for central oncogenes such as c-MYC15,16, which is strongly downregulated upon G4 stabilization by ligands like pyridostatin (PDS)17.” Here, the crucial role of G4s in gene regulation and the methods for regulating G4s are elaborated. This section requires special emphasis and recent advances such as DOI: 10.1038/nchem.1126, 10.1038/s41556-024-01448-1, 10.1002/anie.202503488, 10.1038/s41467-022-29135-5, etc., may consider included in the introduction.

We thank the reviewer for pointing out the need to emphasize the broader significance of G4s in global gene regulation and the rapidly evolving methods to target them. We have expanded this section in the Introduction to underscore the crucial role of G4s in various physiological and pathological contexts. Furthermore, we have explicitly integrated the suggested highly relevant publications, which excellently highlight recent advances from single-molecule analysis to targeted dCas9-based G4 regulation (Page 3, Line 68ff).

2. On page 8 of the paper, “Figure 1D. representative immunofluorescence image of untreated and PDS.” The authors should consider to provide the images of cell nucleus staining and the images showing their co-localization with BG4. This will enable more accurate description of the situation.

We thank the referee, we have updated the images in Figure 1 which now include DAPI staining.

3. Regarding “Figure 1G. FACS analysis of surface marker CD40 indicative of macrophage activation under different conditions.” The authors are suggested to provide representative flow cytometry data in the article or in the supporting information.

In response to this suggestion, we have included the histogram plots to Supplementary Figure 1.

4. On page 12 of the paper, the authors should quantify the WB data in Figure 2E in order to measure the phosphorylation status of NF-κB p65. In addition, the captions for Figures 2E and 2F were put in the wrong order and should be corrected.

We agree, that sometimes bands on a Western blot are easily misleading. We added quantifications for p65 and other subunits as reference to the supplementary. They can be found in Supplementary Figure 2. Quantifications were done from at least three independent experiments.

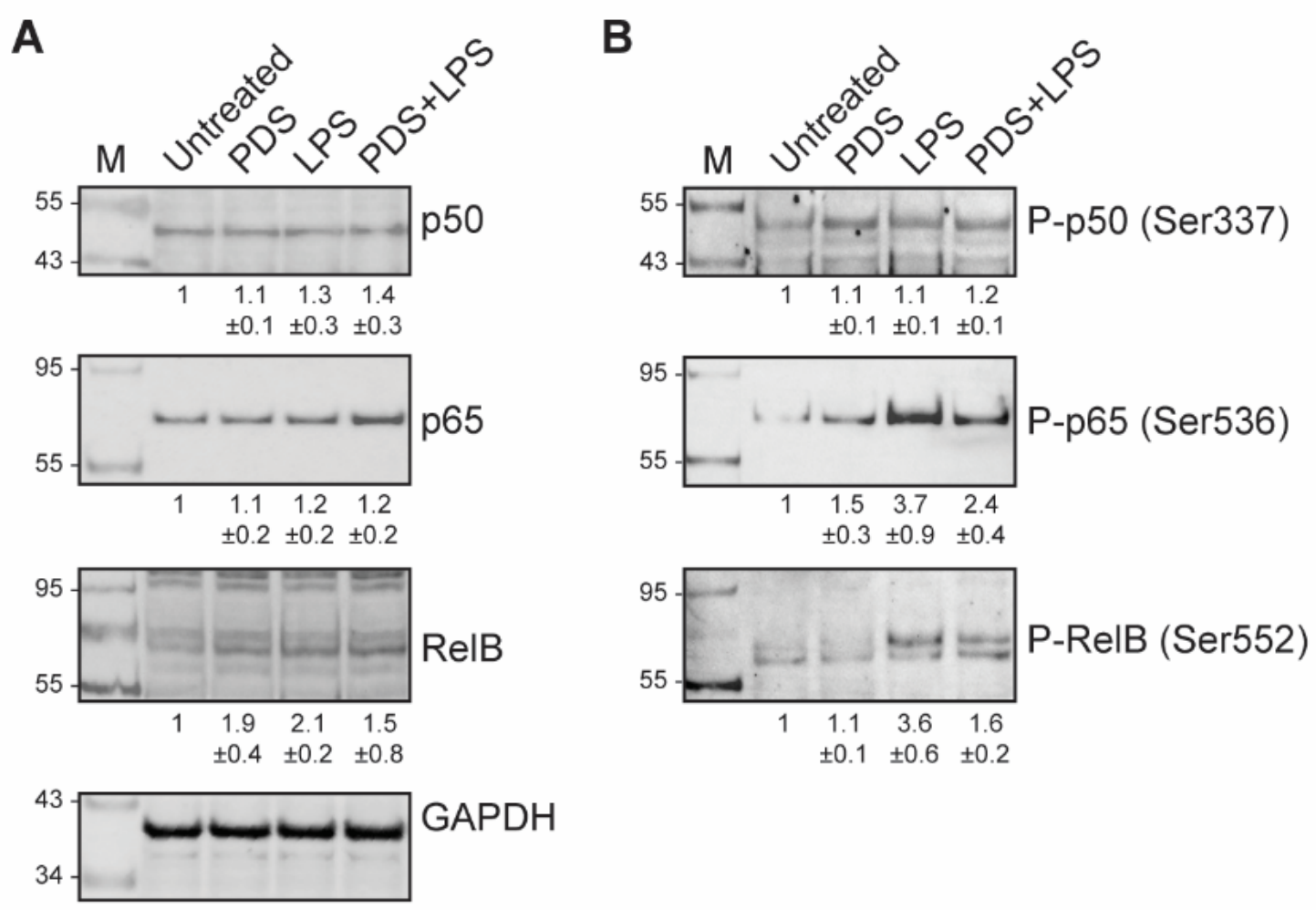

We have corrected the order in the figure legend.

5. Figure 4 C and D predicted overlaps of G4-forming regions (G4Hunter) and NF-κB binding motifs 317 (HOCOMOCO) in the Il1b_1 (C) and Il1b_2 (D) site. A single prediction is not convincing. Please use other software to predict again the overlapping area between the G4 formation region and the NF-κB binding site, and compare the results to jointly support this conclusion.

We appreciate the suggestion and agree that single prediction algorithms may introduce unwanted bias. We have therefore performed the suggested reanalysis using G4RNA screener, which incorporates 3 different G4-prediction engines, including G4 Hunter. For NF-KB we also added an second prediction algorithm (TF BIND), included in the updated Figure

4. Overall, the data of the other algorithms are similar to the G4 Hunter prediction, with one notable exception in the central region of Il1b_2 (G4NN). Importantly, all models predict the strong overlap of the Marsico G4 and the NF-KB binding site in the Il1b_1 region (~50-100bp).

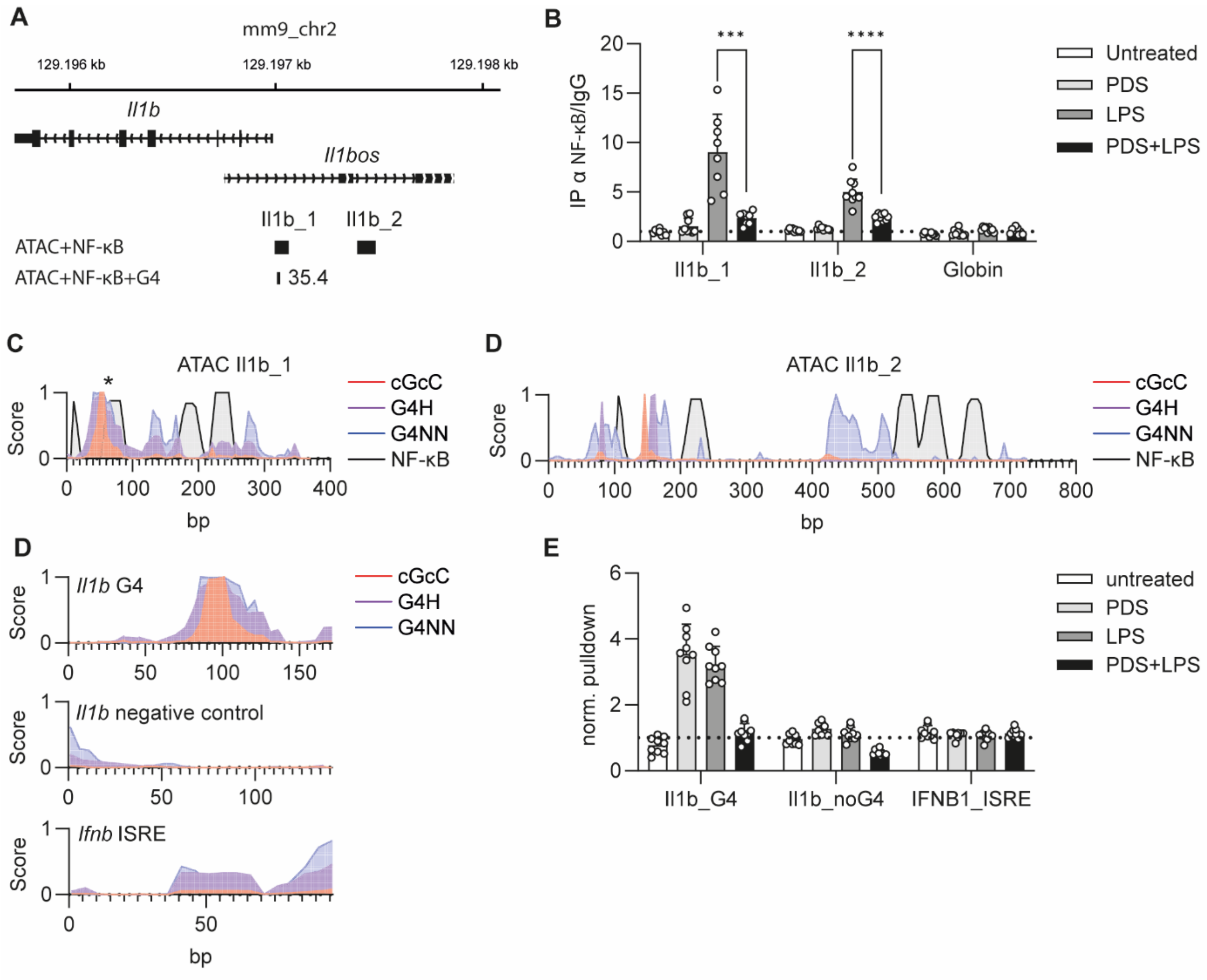

Reviewer #4 (Remarks to the Author):

Shult and colleagues demonstrate that stabilization of G-quadruplexes (G4s) in macrophages (bone marrow–derived macrophages and RAW cells) affects innate immune activation triggered by LPS. They show that NF-κB–dependent inflammatory gene induction is selectively repressed by treatment with the G4 ligand pyridostatin (PDS). Furthermore, they find that G4 stabilization reduces NF-κB occupancy at the IL-1β promoter. Based on EMSA assays, the authors propose that G4 folding physically interferes with NF-κB binding to its consensus DNA motif. This study suggests that G4 formation may be beneficial for modulating excessive inflammatory responses. However, several major issues need to be addressed, as outlined below.

We thank the referee for reading critical our work and for providing very valuable feedback. The suggested new experiments are very useful, and we hope that the newly added Figures and experiments will support our work and will clarify the previous issues.

1. The basis for the selective influence of G4 stabilization on NF-κB signaling remains unclear. The authors report that three of the five most significantly down-regulated pathways following PDS treatment belong to the NF-κB family (Figure 2A); however, the remaining two pathways are not shown and should be indicated.

We thank the reviewer for the thorough assessment and have added mentions of the two other TFs (MLLT3, CRELD1) in the corresponding section of the text. It now reads: "Gene set enrichment analysis (GSEA) of differentially expressed genes revealed that, besides MLLT3 and CRELD1, three of the five most significantly downregulated pathways corresponded to the NF-κB family (REL, NF-KB 1, and RELB), suggesting that G4 stabilization specifically targets NF-κB signaling (Figure 2A)."

More importantly, PDS treatment also markedly suppresses the induction of type I interferon and CXCL10, whose expression is primarily regulated by IRF family transcription factors (IRF3 and IRF7). Therefore, it is necessary to determine whether G4 stabilization also affects IRF recruitment to their cognate cis-regulatory elements. This point should be experimentally addressed.

We appreciate the comment and agree that this is indeed an interesting phenotype that we haven't followed up on. In order to test the hypothesis that IRF3/7 may be similarly regulated by PDS-mediated G4 stabilization we first assessed whether IRF3 is upregulated/activated and activated in iBMDMs after treatments. However, we found no significant regulation after 4h of LPS and/or PDS, as shown in Reviewer Figure R3. We note, however, that IRF3 activation happens on the minutes timescale and may no longer be detectable at the 4h time point.

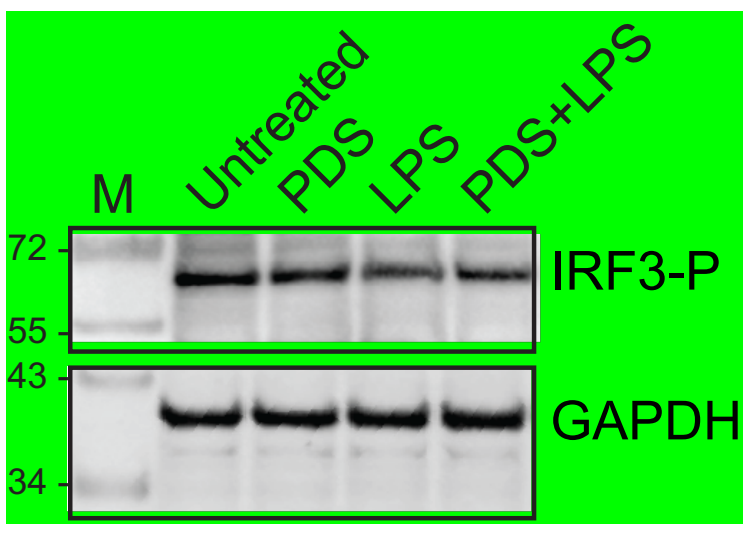

Figure R3: Western blot of phospho-IRF3 upon different treatments in iBMDM.

Moreover, Marsico et al. found no G4 at the *Ifnb1/Cxcl10* ISRE using G4-seq, which we confirmed for *Ifnb1* using G4DP (Figure 4, Reviewer Figure R4). Therefore, G4s do likely not play a role in the IRF3-mediated regulation of these genes under these conditions. Finally, NF-kB is also involved in regulation of these genes (https://www.bu.edu/nf-kb/gene-resources/target-genes/), which makes it likely that these regulatory effects are at least in part due to the observed general perturbation of NF-kB activation.

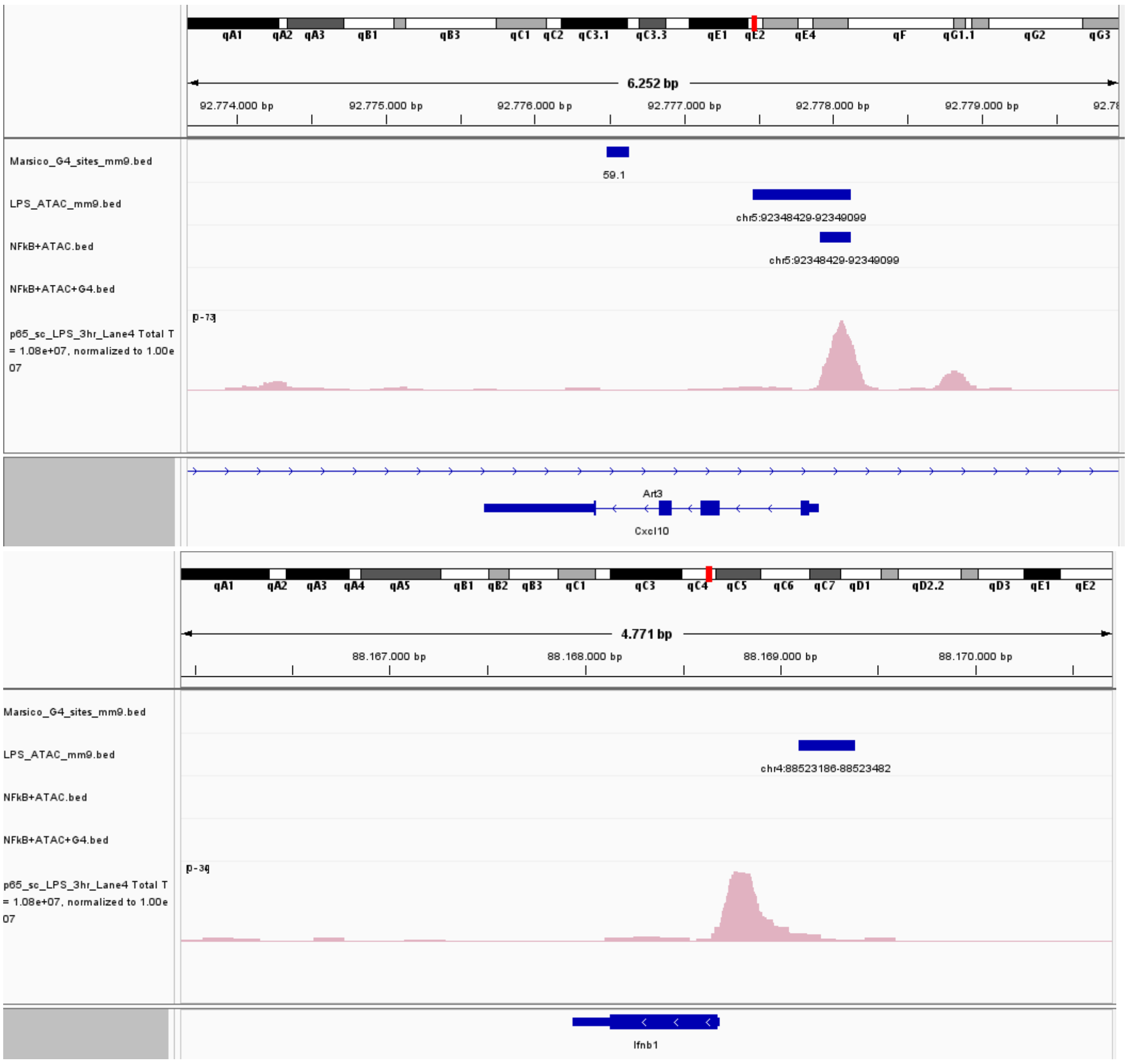

Figure R4: G4s, ATAC Peaks, and NF-kB binding sites in promoter region of Ifnb1 and Cxcl10.

2. Related to comment #1, it remains unclear whether PDS broadly suppresses LPS-inducible gene expression or preferentially affects a subset of genes according to their dependence on NF-κB for activation. In this regard, it would be important to present examples of LPS-inducible genes that are not affected by PDS treatment, if such genes exist (e.g., in Figure 2C).

We are thankful for this insightful comment. Indeed, by filtering our RNASeq data, we found that more than 50 LPS regulated genes (Log2FC>1 vs untreated) were unaffected or even upregulated by PDS co-treatment (ΔLog2FC >-0.3). Interestingly, NF-kB-dependent genes such as Nfkbid were found to be unaffected by PDS treatment. Such proteins are vital to counteract overshooting inflammatory responses, strengthening our hypothesis of a selectively anti-inflammatory G4 function in immune cell programming. Accordingly, the G4-seq data does not show overlapping peaks at the corresponding NF-kB promoter binding sites. Gene ontology analyses of these genes showed a clear enrichment of immunologic pathways, most notably negative regulation of cytokine production. Albeit the low total number of genes, cell-type signature profiling (PaGenBase) also identified a significant enrichment for genes characteristic of unstimulated macrophages (PGB:00277).

Finally, genes strongly induced by LPS, such as endothelin 1 (Edn1), which are not dependent on NF-kB show completely unaltered expression upon PDS co-treatment.

Thus, our data strongly suggest that proinflammatory subsets of the NF-kB responsive transcriptome are selectively regulated by chemical G4-stabilization. We have added overview data of the regulation to the supplement (Supplementary Table 1, Supplementary Figure 2A).

ID	LPS [logFC2]	PDS+LPS [logFC2]	ΔlogFC2
Flt1	1,430	1,161	-0,270
Grap	1,315	1,061	-0,254
Trim30a	1,307	1,066	-0,241
Mcts2	1,131	0,903	-0,228
St18	1,277	1,058	-0,218
Slc11a2	1,477	1,297	-0,180
Rhbdf1	1,720	1,547	-0,173
Hp	1,450	1,283	-0,168
Adgrl2	1,070	0,916	-0,154
Gm42820	1,555	1,458	-0,097
Rel	1,085	1,013	-0,072
Gm42819	1,009	0,937	-0,072
Gm5251	1,427	1,394	-0,033
Csf1	2,340	2,353	0,013
Tent5a	1,580	1,612	0,033
Slc2a6	1,593	1,630	0,037
Uggt2	1,239	1,279	0,040
Edn1	5,287	5,334	0,046
Oasl1	1,575	1,669	0,094
Il1rn	2,670	2,771	0,102
Gm38157	1,507	1,642	0,134
Gm43420	1,124	1,303	0,179
Spink5	2,367	2,596	0,229
Nfkbid	1,584	1,829	0,246
Gm26497	1,324	1,572	0,247
Fam20c	1,579	1,871	0,292
Gm22748	1,591	1,936	0,345
mt-Nd6	1,448	1,838	0,390
4930430E12Rik	1,101	1,544	0,443

Gm37084	1,946	2,416	0,470
Btg3	1,337	1,884	0,547
Gm2223	1,036	1,616	0,580
Fam83e	1,986	2,578	0,592
Cd83	1,997	2,684	0,687
Gm38009	1,097	1,800	0,703
Cd74	1,352	2,071	0,720
Plk2	1,686	2,417	0,732
1700031P21Rik	1,400	2,134	0,734
Gadd45b	1,648	2,397	0,749
Gm7809	1,532	2,312	0,780
Arid5a	1,608	2,405	0,798
Gm26130	1,311	2,173	0,862
Fabp3	1,039	2,058	1,018
Gm15247	1,176	2,314	1,138
Eid3	1,168	2,348	1,180
Atf3	1,413	2,909	1,496
mt-Tv	1,688	3,225	1,537
Adgb	1,380	3,027	1,647
mt-Tr	1,419	3,181	1,762
Egr1	1,888	3,804	1,916
Gdf15	1,363	3,602	2,239
Gm43304	1,409	4,599	3,191

Two examples from the list are shown here:

Nfkbid (NF-kB dependent, anti-inflammatory, no G4 at binding site)

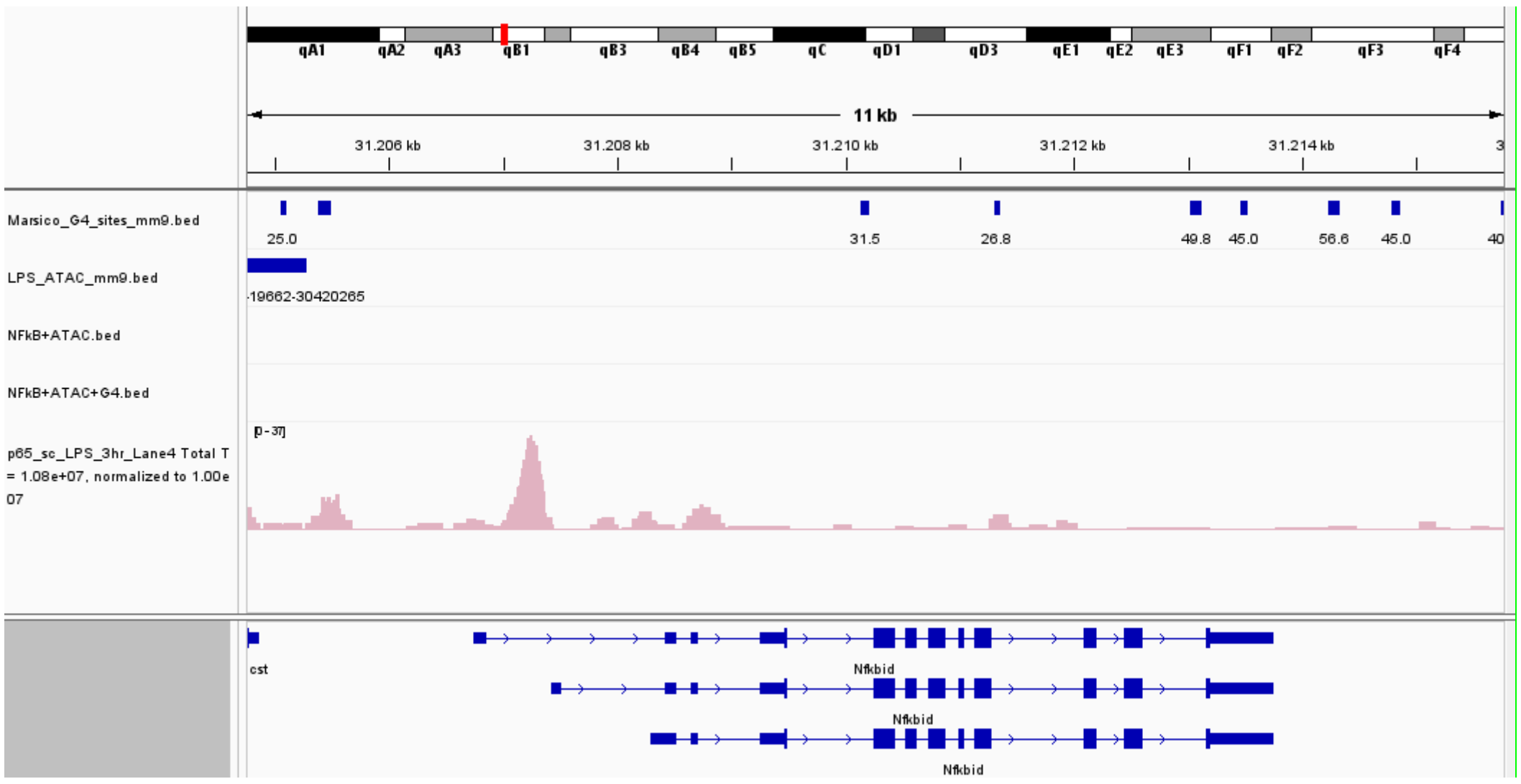

Edn1 (no NF-kB dependence)

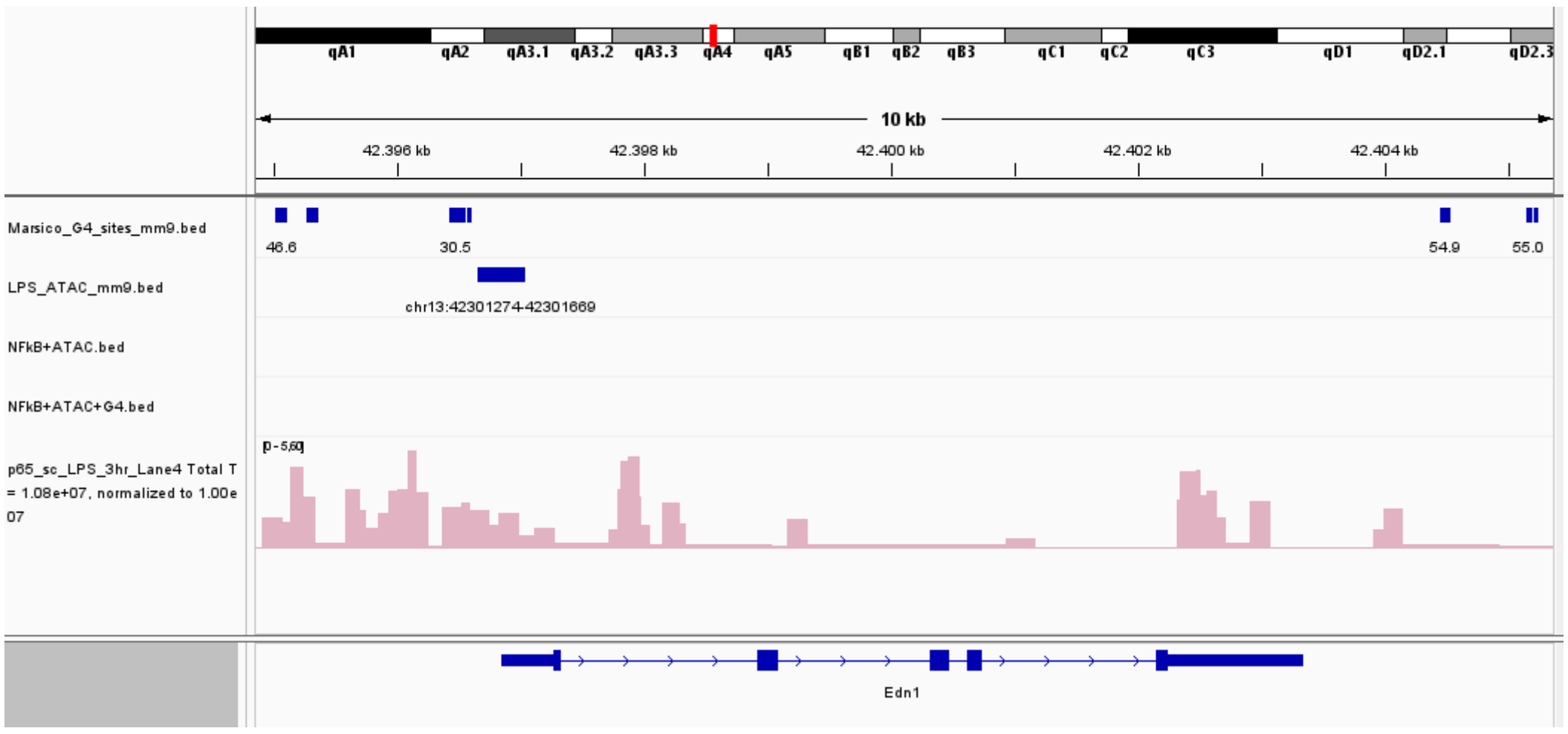

3. In Page 27, Lines 414–416, the authors state: “Reporter assays confirmed that G4 formation only inhibits the transcriptional activation, but not the initial phosphorylation and translocation of NF-κB.” However, it is unclear which experimental data support this conclusion. The authors should explicitly indicate the corresponding figures or provide the relevant data.

We have added a reference to Figure 2, in which the partial phosphorylation is shown.

4. In Figure 5, the source and condition of the lysates used for EMSA are not clearly described. It is unclear whether the lysates were prepared from LPS-stimulated cells or unstimulated cells, and whether NF-κB–DNA binding activity was detected only in lysates from LPS-stimulated cells. The authors should clarify the stimulation conditions and explicitly state the source of the lysates used for the EMSA experiments. Additionally, the authors should address whether the signal that is resistant to NF-κB competition in Figure 5B depends on LPS stimulation or is present under basal conditions.

We thank the reviewer for pointing out this omission. We have updated the manuscript and the legend of Figure 5 to explicitly state the source and stimulation conditions of the lysates.

To clarify: the secondary signal that is resistant to NF-κB competition is present under basal conditions and its intensity does not change upon LPS stimulation. This independence from LPS supports our hypothesis that this band is formed by constitutively active transcription factors, such as SP1.

Regarding NF-κB activity, we also observed binding in both unstimulated and LPS-stimulated lysates. The binding in unstimulated iBMDMs is a known biological feature representing constitutively nuclear p50 homodimers, which act as placeholders or basal repressors on proinflammatory promoters prior to activation.

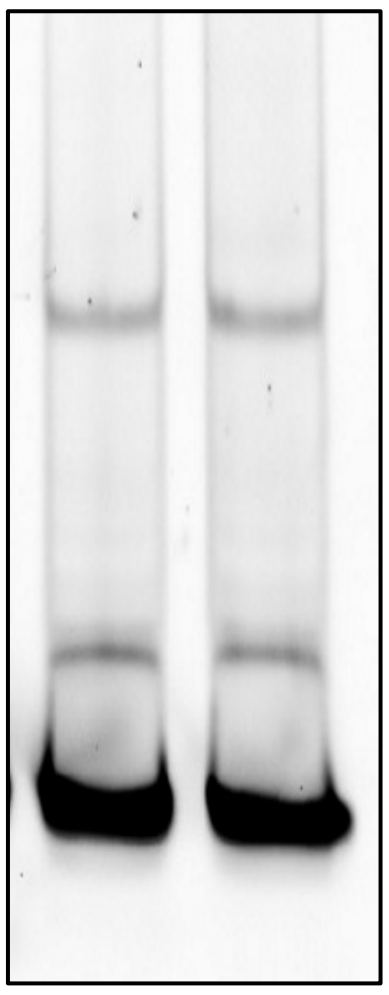

Figure R5: EMSA in lysates from LPS treated vs. untreated (unt) iBMDMs.

Minor point
1. Page 19, Line 306 and 307. “Figure 5C and 5D” should be “Figure 4C and 4D”.
2. Page 20, In the scheme of IL-1b locus, there are two 129.196kbs. One should be 129.197kb?

We have corrected these typos.

Feng, Y., He, Z., Luo, Z., Sperti, F.R., Valverde, I.E., Zhang, W., Monchaud, D., 2022. Chemo- *versus* Immuno-Precipitation of G-Quadruplex-DNA (G4-DNA): A Direct Comparison of the Efficiency of the Antibody BG4 *versus* the Small-Molecule Ligands TASQs for G4 Affinity Capture. SSRN Electron. J. https://doi.org/10.2139/ssrn.4292605

Lago, S., Nadai, M., Cernilogar, F.M., Kazerani, M., Moreno, H.D., Schotta, G., Richter, S.N., 2021. Promoter G-quadruplexes and transcription factors cooperate to shape the cell type-specific transcriptome. Nat. Commun. 12, 3885. https://doi.org/10.1038/s41467-021-24198-2

Li, S., Wang, R., Zhang, J., Cao, C., Bai, J., Cai, Z., Ye, R., Chen, J., Liu, W., Lu, C., Li, R., Xue, Y., 2025. ssG4-seq for global profiling of strand-specific G-quadruplex structures in mammalian genomes. Nat. Commun. 17, 195. https://doi.org/10.1038/s41467-025-66895-2

Marsico, G., Chambers, V.S., Sahakyan, A.B., McCauley, P., Boutell, J.M., Antonio, M.D., Balasubramanian, S., 2019. Whole genome experimental maps of DNA G-quadruplexes in multiple species. Nucleic Acids Res 47, 3862–3874. https://doi.org/10.1093/nar/gkz179

Masson, T., Guetta, C.L., Laigre, E., Cucchiarini, A., Duchambon, P., Teulade-Fichou, M.-P., Verga, D., 2021. BrdU immuno-tagged G-quadruplex ligands: a new ligand-guided immunofluorescence approach for tracking G-quadruplexes in cells. Nucleic Acids Res. 49, 12644–12660. https://doi.org/10.1093/nar/gkab1166

Peterson, J.M., Wang, D.J., Shettigar, V., Roof, S.R., Canan, B.D., Bakkar, N., Shintaku, J., Gu, J.-M., Little, S.C., Ratnam, N.M., Londhe, P., Lu, L., Gaw, C.E., Petrosino, J.M., Liyanarachchi, S., Wang, H., Janssen, P.M.L., Davis, J.P., Ziolo, M.T., Sharma, S.M., Guttridge, D.C., 2018. NF-κB inhibition rescues cardiac function by remodeling calcium genes in a Duchenne muscular dystrophy model. Nat. Commun. 9, 3431. https://doi.org/10.1038/s41467-018-05910-1

Ray, S., Tillo, D., Boer, R.E., Assad, N., Barshai, M., Wu, G., Orenstein, Y., Yang, D., Schneekloth, J.S., Vinson, C., 2020. Custom DNA Microarrays Reveal Diverse Binding Preferences of Proteins and Small Molecules to Thousands of G-Quadruplexes. ACS Chem. Biol. 15, 925–935. https://doi.org/10.1021/acschembio.9b00934

Turcotte, M.-A., Bolduc, F., Vannutelli, A., Mitteaux, J., Monchaud, D., Perreault, J.-P., 2023. Development of a highly optimized procedure for the discovery of RNA G-quadruplexes by combining several strategies. Biochimie 214, 24–32. https://doi.org/10.1016/j.biochi.2023.07.014

Reviewer #1 (Remarks to the Author):

The revised manuscript has been substantially improved through the addition of multiple new experiments, including validation of additional NF-κB subunits, G4 pulldown assays at the Il1b locus, EMSA experiments under dynamic folding conditions. Most of the raised concerns have been addressed in a substantive and satisfactory manner. However, one major concern remains regarding the genome-wide scope of the proposed mechanism. The central claim of this study that G-quadruplex formation broadly modulates NF-κB-dependent transcription during inflammation relies heavily on bioinformatic predictions overlain with publicly available G4-seq datasets, combined with focused experimental validation at a single locus (Il1b). While the newly added G4 pulldown data provide valuable direct evidence for G4 dynamics at the Il1b promoter, this represents only one genomic site.
Genome-wide G4 profiling under the relevant treatment conditions (untreated, PDS, LPS, PDS+LPS) is critical to establish that the observed mechanism is not an idiosyncrasy of the Il1b locus but instead reflects a global regulatory principle. Without transcriptome-scale G4 mapping data, the causal link between genome-wide NF-κB occupancy changes and G4 stabilization remains correlative and inferred rather than directly demonstrated. The authors are strongly encouraged to perform genome-wide G4 profiling under their experimental conditions to substantiate the global nature of the G4–NF-κB regulatory axis.

We acknowledge the Reviewer's concern and have indicated in the discussion that additional work is needed to show that the effects can be generalized. Therefore, we added the following sentence: "Future global G4 profiling will be critical to determine if this mechanism represents a genome-wide regulatory principle."

Reviewer #2 (Remarks to the Author):

The authors have now addressed my criticisms and I am supportive of publication in Nature Comms

Reviewer #3 (Remarks to the Author):

I recommend this revied manuscript for publication as is.

Reviewer #4 (Remarks to the Author):

The authors have productively addressed all concerns and comments raised in my review. I have no further comments.
